# The Original Form of C_4_-Photosynthetic Phospho*enol*pyruvate Carboxylase Is Retained in Pooids but Lost in Rice

**DOI:** 10.3389/fpls.2022.905894

**Published:** 2022-07-25

**Authors:** Naoki Yamamoto, Wurina Tong, Bingbing Lv, Zhengsong Peng, Zaijun Yang

**Affiliations:** ^1^Key Laboratory of Southwest China Wildlife Resources Conservation (Ministry of Education), College of Life Science, China West Normal University, Nanchong, China; ^2^College of Environmental Science and Engineering, China West Normal University, Nanchong, China; ^3^School of Agricultural Science, Xichang College, Xichang, China

**Keywords:** abiotic stress, gene function and evolution, grass genome evolution, nitrate response, phospho*enol*pyruvate carboxylase, positive selection, ppc1b, Pooideae

## Abstract

Poaceae is the most prominent monocot family that contains the primary cereal crops wheat, rice, and maize. These cereal species exhibit physiological diversity, such as different photosynthetic systems and environmental stress tolerance. Phospho*enol*pyruvate carboxylase (PEPC) in Poaceae is encoded by a small multigene family and plays a central role in C_4_-photosynthesis and dicarboxylic acid metabolism. Here, to better understand the molecular basis of the cereal species diversity, we analyzed the *PEPC* gene family in wheat together with other grass species. We could designate seven plant-type and one bacterial-type grass *PEPC groups*, ppc1a, ppc1b, ppc2a, ppc2b, ppc3, ppc4, ppcC_4_, and ppc-b, respectively, among which ppc1b is an uncharacterized type of *PEPC*. Evolutionary inference revealed that these *PEPCs* were derived from five types of ancient *PEPCs* (*ppc1*, *ppc2*, *ppc3*, *ppc4*, and *ppc-b*) in three chromosomal blocks of the ancestral Poaceae genome. C_4_-photosynthetic *PEPC* (*ppcC_4_*) had evolved from *ppc1b*, which seemed to be arisen by a chromosomal duplication event. We observed that *ppc1b* was lost in many *Oryza* species but preserved in Pooideae after natural selection. *In silico* analysis of cereal RNA-Seq data highlighted the preferential expression of *ppc1b* in upper ground organs, selective up-regulation of *ppc1b* under osmotic stress conditions, and nitrogen response of *ppc1b*. Characterization of wheat *ppc1b* showed high levels of gene expression in young leaves, transcriptional responses under nitrogen and abiotic stress, and the presence of a Dof1 binding site, similar to *ppcC_4_* in maize. Our results indicate the evolving status of Poaceae PEPCs and suggest the functional association of *ppc1*-derivatives with adaptation to environmental changes.

## Introduction

Poaceae (the grass family) is the largest group for monocot plant species and contains several primary cereal crops, such as wheat, rice, maize, and sorghum. These cereals are categorized into three subfamilies, *Pooideae*, *Ehrhartoideae*, and *Panicoideae*. *Pooideae* includes important cereals, wheat, barley, and oats and is represented by a model grass *Brachypodium distachyon* (The International Brachypodium Initiative, 2010). *Ehrhartoideae* contains rice, and *Panicoideae* does maize and sorghum (Prasad et al., 2011). These three taxonomic groups were established with complex genomic events (Salse et al., 2008; Jiao et al., 2014). Grass species exhibit various physiological properties in photosynthetic systems and abiotic stress tolerance (Pardo and VanBuren, 2021). Understanding of the molecular basis of the diversity of grass species is necessary to develop useful cultivated cereal plants further.

One of the carbon-fixation enzymes, phospho*enol*pyruvate carboxylase (PEPC), is indispensable to plant individuals. PEPC catalyzes the irreversible β-carboxylation of phospho*enol*pyruvate (PEP) by incorporating HCO_3_^−^ to yield oxaloacetate (O’Leary et al., 2011). This enzyme is well recognized as the central photosynthetic enzyme in C_4_ and Crassulacean acid metabolism (CAM) plants while it works on the anaplerotic provision of carbon skeletons to the citrate acid cycle in bacteria (Kai et al., 2003; von Caemmerer and Furbank, 2016). Plant PEPCs play roles in nitrogen metabolism, fatty acid biosynthesis, and respiration (O’Leary et al., 2011; Shi et al., 2015; Yamamoto et al., 2015, 2020). In addition, PEPC might act on re-fixing CO_2_ released by respiration and is likely to act on abiotic stress adaptation (Sánchez et al., 2006; O’Leary et al., 2011; Kandoi et al., 2016).

The plant genomes maintain four to 10 *PEPC* isogenes (Sánchez et al., 2006; Masumoto et al., 2010; Wang et al., 2016; Waseem and Ahmad, 2019; Zhao et al., 2019). In plants, two primary PEPC classes are defined: plant-type and bacterial-type PEPC (O’Leary et al., 2011). Plant-type PEPCs are categorized into photosynthetic types comprising C_4_-photosynthetic PEPC, CAM-type PEPC, and non-photosynthetic type ones. Most non-photosynthetic PEPCs are cytosolic isoforms, but a chloroplast-targeted isoform was found in grass species rice (Masumoto et al., 2010). Cytosolic PEPCs are predominant in plant species with multiple isoforms, from which gene expression patterns differ (Caburatan and Park, 2021). In rice, four cytosolic PEPCs, one chloroplast PEPC, and one bacterial-type PEPC are known, but biological roles of these PEPCs seem to be not identical (Masumoto et al., 2010; Yamamoto et al., 2014a, 2015). The chloroplast-targeted PEPC Osppc4 supports ammonium assimilation in rice (Masumoto et al., 2010). Previous phylogenetic studies approached the evolutionary history of photosynthetic PEPCs in monocot species (Christin et al., 2007; Christin and Besnard, 2009; Deng et al., 2016). However, the origin and the evolutionary processes of grass PEPCs remain obscure. This fact is due to the complexity of the grass genome evolution, technical issues in the phylogenetic analyses, and unknown *PEPC* isogene compositions in *Pooideae* and *Panicoideae*.

**Figure 1:**
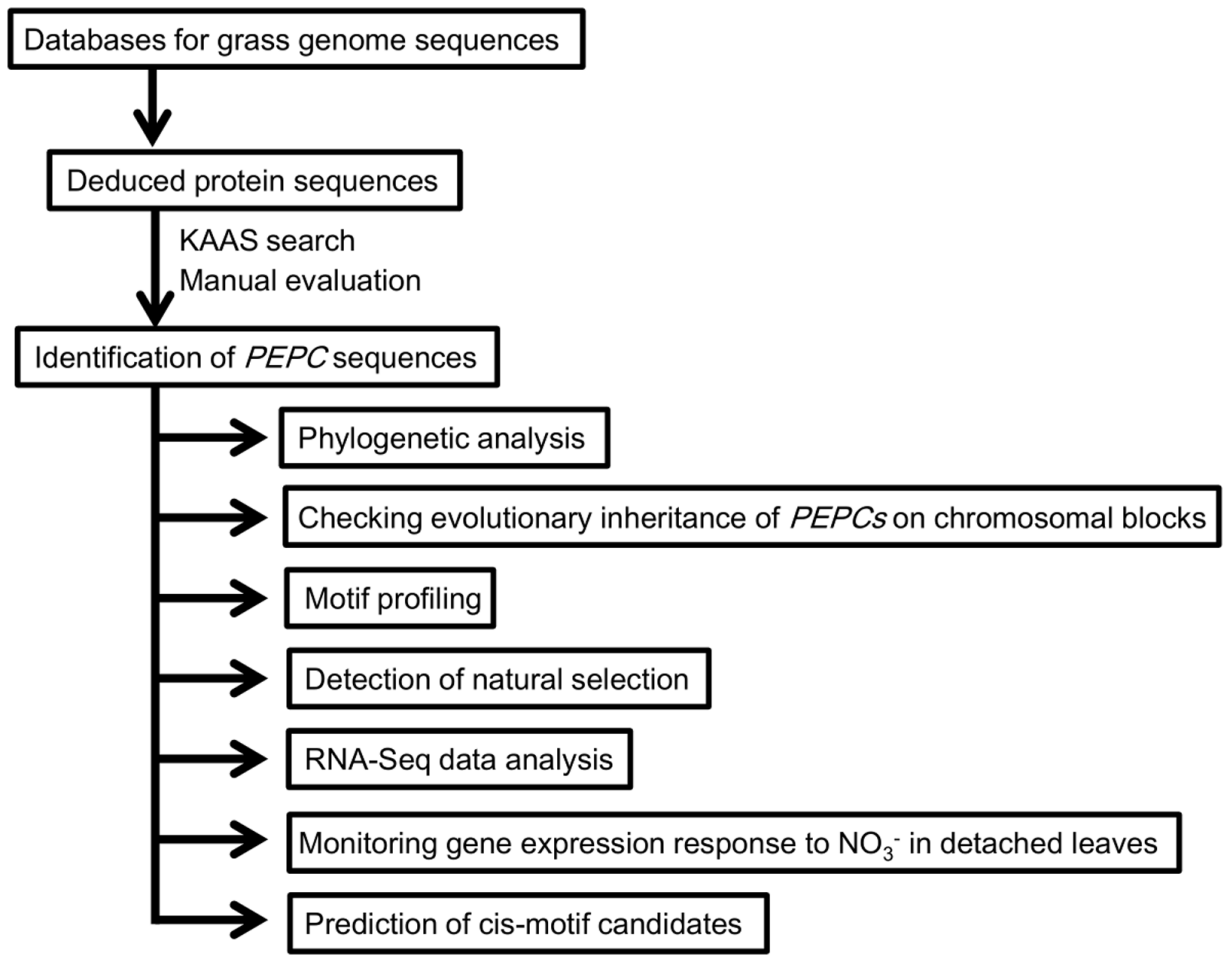
Schematic representation of cross-species genome-wide analysis of grass Phospho*enol*pyruvate carboxylases (PEPCs).

In the present study, we conducted a cross-species genome-wide analysis of PEPCs in wheat and other grass species (the analytical scheme is shown in Figure 1). Our analyses identified wheat *PEPCs*, clarified the evolutionary history of grass PEPCs driven by chromosomal-level duplications, and revealed gene expression patterns of model grass PEPCs associated with environmental changes. We discovered a PEPC isoform group “ppc1b,” which is the natural origin of C_4_-photosynthetic PEPCs and originated from one of the ancient grass PEPC *ppc1*. RNA-Seq data analyses indicated abiotic stress responses of *ppc1b* in wheat, *T. turgidum*, *Brachypodium*, and barley. Verification of nitrogen-dependent response of PEPC in wheat revealed the selective response of *ppc1b*. We found evolutionary conservation of a Dof1 transcription factor binding site in the *ppc1b* promoter region. Overall, we represent the evolutionary history of grass PEPCs and designate the molecular groups of grass PEPCs for framing grass PEPC research. Finally, we discuss the biological significance of the molecular plasticity of ppc1b.

## Materials and Methods

### Mining of PEPC Protein Sequences

The predicted protein sequences in *Triticum aestivum* L. (cultivar Chinese Spring) were collected from Ensembl Plant (Bolser et al., 2017, release 46) and queried against the KEGG metabolic pathway database^1^ by the assignment method of KAAS searches as a bi-directional best hit (Moriya et al., 2007). Proteins with the KEGG Orthology identifier K01595 (phospho*enol*pyruvate carboxylase) were tentatively designated as PEPC and subjected to manual check to retain only reliable PEPC sequences. The wheat PEPC loci were named according to the homologies to the potential counterpart isoforms in rice. Predicted protein sequences in other 24 monocot species, *Aegilops tauschii*, *B. distachyon*, *Eragrostis tef*, *Hordeum vulgare* cultivar Morex and Golden Promise, *Leesa perrieri*, *Musa acuminata*, *Oryza barthii*, *O. brachyantha*, *O. glaberrima*, *O. glumaepatula*, *O. longistaminata*, *O. rufipogon*, *O. meridionalis*, *O. nivara*, *O. punctata*, and *O. sativa* indica varieties 93–11 and R498, *O. sativa* japonica variety kitaake, *Panicum hallii*, *Setaria italica*, *S. viridis*, *Sorghum bicolor*, *T. dicoccoides*, *T. spelta*, *T. turgidum*, and *Zea mays*, were also collected from Ensembl Plant. Predicted sequences for *O. meyeriana* var.granulata were from GenBank (accession: SPHZ00000000.2). Predicted protein sequences for *Phyllostachys edulis* were from BambooGBD (Zhao et al., 2018). All the protein sequences of *O. officinalis* (Shenton et al., 2020) were also collected. PEPC proteins for these protein data sets were also searched as mentioned above (Supplementary Table S1).

### Analysis of mRNA Sequencing Data

Public RNA sequencing (RNA-Seq) profiles in wheat were downloaded from the U.S. National Center for Biotechnology Information Sequence Read Archive^2^ for monitoring the organ-specific gene expression patterns of *PEPC* isogenes in the reference cultivar Chinese Spring. The analyzed profiles for spatial gene expression patterns included 16 organs, including root, leaf, and stem (Supplementary Table S2). RNA-Seq profiles in grass species under abiotic stress conditions were also analyzed (Supplementary Table S2). These sequencing reads were trimmed by Trimmomatic version 0.39 (Bolger et al., 2014), and obtained high-quality reads were mapped on the reference genome of wheat using DART version 1.3.6 (Lin and Hsu, 2018) with the default condition. Generated bam files were sorted by SAMtools version 1.9 (Li et al., 2009) and processed by featureCounts version 2.0.1 (Liao et al., 2014) to obtain read counts per gene. The obtained count data were normalized by TCC version 1.30.0 with the iDEGES/edgeR method (Sun et al., 2013) and converted into transcript-level or gene-level (TPM) values. Averaged TPM values were applied in case replicated samples were available. HeatMapper (Verhaak et al., 2006) was used to visualize of the spatial gene expression patterns of wheat *PEPC* isogenes. Principal component analysis (PCA) of the gene expression levels for PEPC was conducted using the R statistical software^3^ with the multivariate exploratory data analysis package FactoMineR version 2.4 (Lê et al., 2008).

### Plant Materials

Seeds of the common wheat variety Chinese spring were soaked with 0.01% KMnO_4_ solution for 30 min to sterilize their surface. After washing with purified water several times, the seeds were incubated in water at 4°C for 3 days. Then, the seeds were placed on wet filter papers under a dark condition. Germinating seeds were transplanted into a mixture of vermiculite and perlite (ratio 2:1) on plastic plant growth trays and placed in a laboratory space under natural light with a Murashige and Skoog-based medium, which lacks nitrogen nutrition (Supplementary Table S3). After 2 weeks, the bases of the main leaf blades were cut using a razor, and the detached leaves were incubated with 40 mM KNO_3_ solution or a mock solution of 20 mM K_2_SO_4_ (added SO_4_^2−^ instead of NO_3_^−^ with the same strength of K^+^ ion) under a fluorescent light (approx. 15,000 lux) at 22°C with humidity of 60–70% (Supplementary Figure S1). The detached leaves at 0, 3, 6, 12, and 24 h after the treatment were harvested and quickly frozen in liquid nitrogen to be stored at −80°C until use.

### Measurement of PEPC Activity and Protein Expression

Soluble proteins including PEPC were extracted from powdered leaf tissues in a buffer [100 mM Tris–HCl (pH 7.8); 1 mM EDTA; 1 mM 2-mercaptoethanol; 10% (w/v) glycerol] with Complete Protease Inhibitor Cocktail (F. Hoffmann-La Roche Ltd., Basel, Switzerland) at 4°C by using a motor and pestle. The homogenate was centrifuged at 13,000 *g* for 20 min, and the resultant supernatant was used for measuring PEPC activity by coupling with the malate dehydrogenase reaction according to the procedures of Yamamoto et al. (2014a). This PEPC assay was carried out in 2 mL of a solution containing 50 mM Tricine-KOH (pH 8.3), 5 mM MgSO_4_, 0.15 mM NADH, 5 mM KHCO_3_, 5 mM PEP (cyclohexylammonium salt), 4 mM DTT, and 3 U of pig heart malate dehydrogenase at 25°C. Three biological replicates were used for PEPC assays with two technical replicates. According to the manufacturer’s protocol, we determined soluble protein content by using Bradford Protein Assay Kit (TIANGEN BIOTECH CO., LTD, Beijing, China). Chromogenic Western blot analysis of PEPCs was carried out using a polyclonal antibody for maize leaf PEPC (Abcam plc, Cambridge, United Kingdom). The NBT/BCIP reaction scheme detected PEPC proteins.

### Wheat RNA Preparation and cDNA Synthesis

Frozen leaves were powdered using a motor and pestle with liquid nitrogen. Approximately, 50 mg of the powdered sample was used for total RNA extraction with LABGENE plant RNA Isolation Kit (LABGENE Biotechnology Co., Ltd., Chengdu, China) according to the manufacturer’s protocol. The extracted total RNAs were analyzed by NanoDrop2000c Spectrophotometer (Thermo Fisher Scientific, Waltham, MA, United States) and gel electrophoresis to verify the quantity and the integrity. Approximately, 1 μg of total RNA fraction with no less than 1.9 of A260/A280 and 1.8 of A260/A230 was applied for first-strand cDNA synthesis using PrimeScript RT Reagent Kit with gDNA Eraser (Takara Bio Inc., Kusatsu, Japan).

### cDNA Cloning and Prokaryotic Expression of Wheat PEPC

Phospho*enol*pyruvate carboxylase (PEPC) cDNAs were amplified from first-stranded cDNAs synthesized from wheat leaf RNAs using gene-specific primers using KOD plus (Toyobo Co. LTD., Osaka, Japan). The amplified fragments were cloned into pMD19-T, and the insert sequences were determined by primer walking. The cDNA sequences of full-length wheat PEPC cDNAs were deposited to Genbank [accession number: ON055387-ON055389]. these will be released after acceptance of this manuscript.

The 678 bp cDNA fragment of *Tappc1bD*, which corresponded to its consensus N-terminal region of Tappc1b, was subcloned into the *Eco*RI and *Hind*III site of pET28a^(+)^, and the constructed recombinant expression construct was transformed into Escherichia coli BL21 (DE3). Three independent *Escherichia coli* transformants were applied to the functional expression of the recombinant PEPC fragment assays. In the assays, transformed *E. coli* colonies were inoculated on LB medium and shaken at 180 rpm for 12 h at 30°C. Then 1 mL of the bacterial culture was added to 30 mL of fresh LB medium and shaken at 180 rpm at 37°C until OD = 600 reached 0.5. After adding IPTG at the final concentration of 0.4 mM, the bacterial cultures were shaken at 180 rpm at 25°C for 5 h. *E. coli* cells were collected by centrifugation and frozen at –80°C until soluble protein extraction. PEPC activities were measured using “Phospho*enol*pyruvate Carboxylase Activity Assay Kit, Ultraviolet Colorimetric Method” (Sangon Biotech Co., Ltd., Shanghai, China).

### Quantitative RT-PCR

The valid concentrations of cDNAs were estimated based on Ct value differences in a preliminary qRT-PCR assay for an *actin* gene (GenBank ID: AB1811991; Wei et al., 2015) and determined dilution factors of the original first-strand cDNA fractions to adjust the internal actin concentrations among samples. Then, 0.5 μL of the diluted cDNA fractions were assayed in qPCR using TB Green Premix Ex Taq II (Takara Bio Inc.) and gene-specific primers (Supplementary Table S4). Biologically triplicate assays were carried out with at least two technical replicates. Relative gene expression levels to *actin* were calculated by the 2^-ΔΔ*C*T^ method (Livak and Schmittgen, 2001).

### Comparison of Promoter Sequences of PEPC

Three-kilo base upstream regions of the transcribed regions for *PEPC* isogenes were retrieved. Potential cis-regulatory motifs were searched using Multiple Em for Motif Elicitation (MEME; Bailey et al., 2015). The search was performed by the discriminative mode, which allowed for mining motifs seen in *Tappc1b*, *ppcC_4_* isogenes, and *Tappc4* but not found in the other eight *PEPC* isogenes in wheat.

### Construction and Analyses of Phylogenetic Trees

Phospho*enol*pyruvate carboxylase protein sequences of the grass species with *Oryza sativa* cultivar Nipponbare were aligned by MUSCLE (Edgar, 2004) implemented in MEGA 7 with gap open penalties of −2.9, hydrophobicity multiplier of 1.2, eight-time iterations of the UPGMA method (Tamura et al., 2013). The aligned sequences were used for reconstructing a phylogenetic tree by the maximum likelihood (ML) method with “WAG with Freqs. (+F) model” and the conditions of partial deletion of gaps at 95%. We added several PEPC sequences of non-plant species, including algae (*Chlamydomonas reinhardtii*, *Ostreococcus tauri*, and *Anabaena* sp.), bacterial species (*Synechocystis* sp., *Marchantia polymorpha*, and *Escherichia coli*), and an Archaea species, *Ferroglobus placidus* to be out grouped. Five hundred bootstrap trials tested the reliability of branches in the tree.

Using TreeSAAP version 3.2 (Woolley et al., 2003), potential positive selection sites were searched with the default setting parameters. Branch-level and branch-site level natural selection was detected using EasyCodeML version 1.0 (Gao et al., 2019) with the default condition using an ML phylogeny of plant-type PEPCs in *A. tauschii*, *B. distachyon*, *O. nivara*, *O. sativa*, *O. rufipogon*, *S. italica*, *S. bicolor*, *Z. mays*, and wheat.

### Statistical Analysis

Student’s *t*-test was applied for numerical data by using Microsoft Excel. Statistical significance was determined with value of *p* < 0.05 or 0.01.

## Results

### Grass PEPCs Belong to Eight Molecular Groups

Our initial question was which molecular types of PEPCs are encoded in the wheat genome. The automated prediction allowed identifying 16 *PEPC* isogenes, comprising 14 plant-type PEPC and two bacterial-type PEPC: these were categorized into six isogene types: *Tappc1a*, *Tappc1b*, *Tappc2*, *Tappc3*, *Tappc4*, and *Tappc-b* (Table 1). These PEPCs were distributed in 11 wheat chromosomes: 3A, 3B, 3D, 5A, 5B, 5D, 6A, 6B, 6D, 7A, and 7D. Except for the bacterial-type PEPC *Tappc-b* and one of the plant-type *PEPC* isogene groups *Tappc1b*, of which orthologue in rice was not found, *PEPC* isogenes were conserved across the A, B, and D genomes. We found additional *PEPC* isogenes *Tappc1bB* on 7B chromosome and *Tappc-bA* on 3A chromosome by TBLASTN searches, although the predicted protein sequences were not assigned to PEPC with our above-mentioning criteria. The presence of *Tappc1b* in the A, B, and D genomes were validated by RT-PCR using gene-specific UTR primers. The amplified cDNA fragments were cloned to be sequenced by primer walking. The transcribed sequences were perfectly matched with the reference genome sequences with confirmation of the exon-intron structure of *Tappc1b* in the GT-AG rule. Prokaryotic expression of a Tappc1b N-terminal polypeptide indicated that *Tappc1b* isogenes encode PEPC proteins (Supplementary Figure S2).

**Table 1:** PEPC isogenes in the wheat genome.

Gene identifier	Chromosome	Gene name	Type of PEPC	Length of protein sequence	Orthologous isogene in rice
*TraesCS6A02G195600*	6A	*Tappc1aA*	Plant-type	968	*Osppc1*
*TraesCS6B02G223100*	6B	*Tappc1aB*	Plant-type	968	*Osppc1*
*TraesCS6D02G183200*	6D	*Tappc1aD*	Plant-type	968	*Osppc1*
*TraesCS7A02G345400*	7A	*Tappc1bA*	Plant-type	967	*–*
*TraesCS7B02G237900* ^*^	7B	*Tappc1bB*	Plant-type	967	*–*
*TraesCS7D02G333900*	7D	*Tappc1bD*	Plant-type	967	*–*
*TraesCS5A02G181800*	5A	*Tappc2A*	Plant-type	972	*Osppc2*
*TraesCS5B02G179800*	5B	*Tappc2B*	Plant-type	972	*Osppc2*
*TraesCS5D02G186200*	5D	*Tappc2D*	Plant-type	972	*Osppc2*
*TraesCS3A02G306700*	3A	*Tappc3A*	Plant-type	966	*Osppc3*
*TraesCS3B02G329800*	3B	*Tappc3B*	Plant-type	966	*Osppc3*
*TraesCS3D02G295200*	3D	*Tappc3D*	Plant-type	966	*Osppc3*
*TraesCS3A02G134200*	3A	*Tappc4A*	Plant-type	1,003	*Osppc4*
*TraesCS3B02G168000*	3B	*Tappc4B*	Plant-type	1,002	*Osppc4*
*TraesCS3D02G150500*	3D	*Tappc4D*	Plant-type	1,003	*Osppc4*
	3A	*Tappc-bA*	Bacterial-type		*Osppc-b*
*TraesCS3B02G008500*	3B	*Tappc-bB*	Bacterial-type	1,046	*Osppc-b*
*TraesCS3D02G005000*	3D	*Tappc-bD*	Bacterial-type	1,048	*Osppc-b*

* These genes were mined manually using TBLASTN searches.

To be consistent with our knowledge, we found typical conserved PEPC motifs such as the phosphorylation site of plant-type PEPC, catalytic bases, glucose-6-phosphate binding site, hydrophobic pockets, PEP binding site, tetramer formation, Mg^2+^ binding site, HCO_3_^−^ binding site, and PEP and Asp binding site (Supplementary Figure S3). Tappc4 in A, B, and D genomes contained plastid transit peptides on the N termini, found in the rice ortholog Osppc4 (Masumoto et al., 2010). Although the total branch lengths of plant-type PEPCs exhibited no obvious difference, it is clear that ppc2 and ppc3 are likely to be more evolved from the common ancestor of the PEPCs analyzed (Supplementary Figure S4).

**Figure 2:**
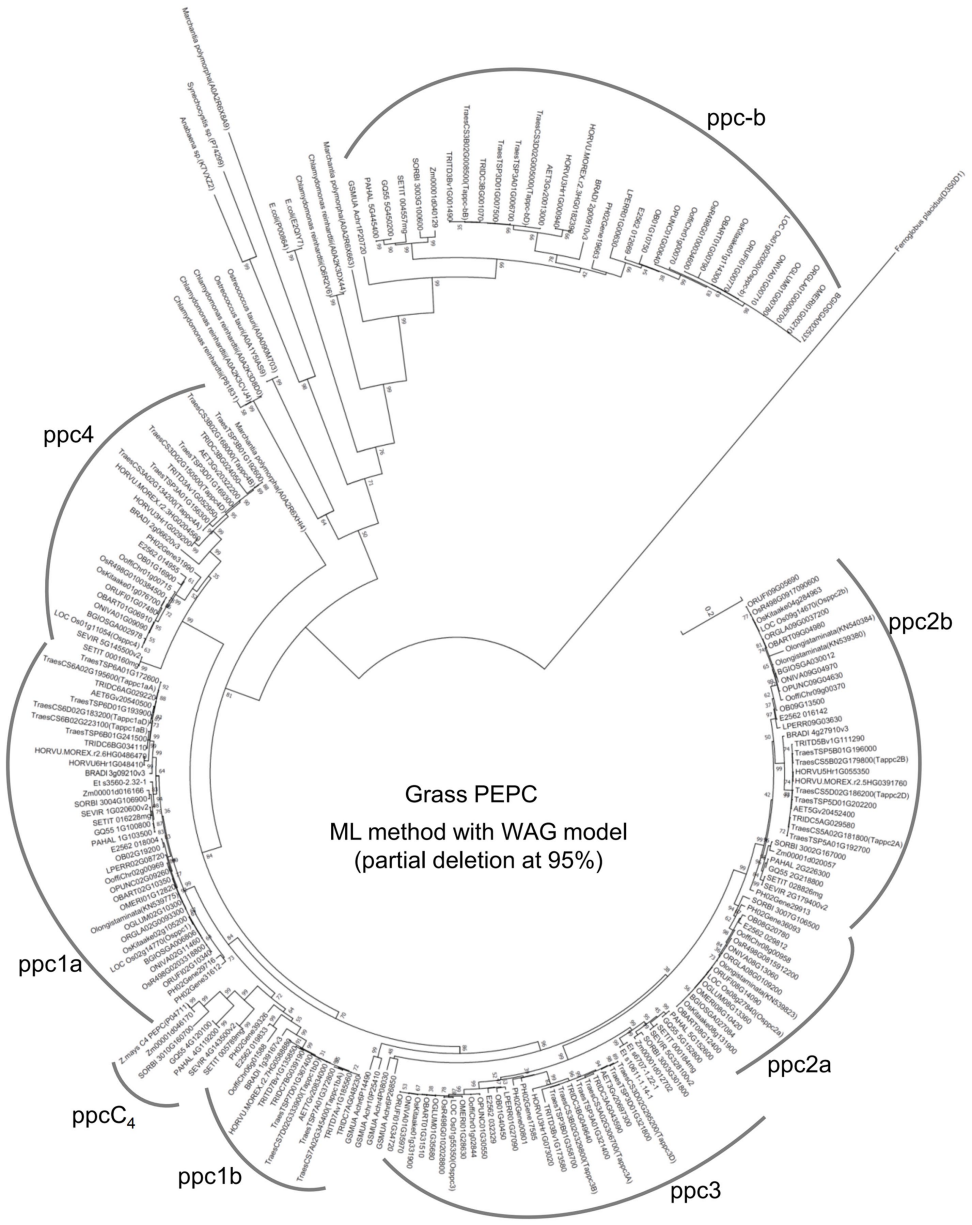
An maximum likelihood (ML) phylogenetic tree dissects grass PEPC proteins. Out-grouped PEPCs were shown as follows; *Chlamydomonas reinhardtii* (P81831, A0A2K3CVJ4, A0A2K3D8D0, Q6R2V6, and A0A2K3DX44), *Ostreococcus tauri* (A0A1Y5IAS9, A0A090M703), *Anabaena* sp. (K7VXZ2), *Synechocystis* sp. (P74299), *Marchantia polymorpha* (A0A2R6X663), *Escherichia coli* (P00864, E2QIY7), and *Ferroglobus placidus* (D3S0D1). The log likelihood for this tree was “−37030.92.”

To assess the evolutionary relationship of the wheat *PEPC* isogenes and other grass *PEPCs*, we performed a phylogenetic analysis of PEPC protein sequences of 28 monocot species. The phylogenetic tree with the maximum likelihood (ML) method dissected the PEPC groups in grass species (Figure 2); in addition to C_4_-photosynthetic PEPC group “ppcC_4_,” seven lineages (groups) of “ppc-b,” “ppc4,” “ppc1a,” “ppc1b,” “ppc3,” “ppc2a,” and “ppc2b” were designated. The single lineage ppc-b is for the bacterial-type PEPC. The chloroplast-type PEPC group ppc4 is located in the first branch among plant-type PEPCs. The second branch among plant-type PEPCs comprised ppc1a, ppc1b, and ppcC_4_, which must have a common ancestral PEPC. The ppc1b group exhibited faster evolving than ppc1a after diversifying these two close lineages. Notably, ppc1b was possibly the origin of ppcC_4_, of which evolutionary speed seemed accelerated after the diversification from ppc1b. The same phylogenic relationship of ppc1b and ppcC_4_ was observed by the ML method with an alternative condition and neighbor-joining phylogenetic trees under eight different parameter settings (Supplementary Figure S5). The third branch among plant-type PEPCs is ppc3, which showed evolutionary conservation between wheat and *Oryza* species. The last branch is ppc2, which comprises ppc2a and ppc2b, where different gene duplication between *Pooideae* and *Oryza* species was observed; *ppc2a* was uniquely present in the *Oryza* genomes.

### Genomic Evidence for the Evolutionary Retention and Changes of Grass PEPCs

**Figure 3:**
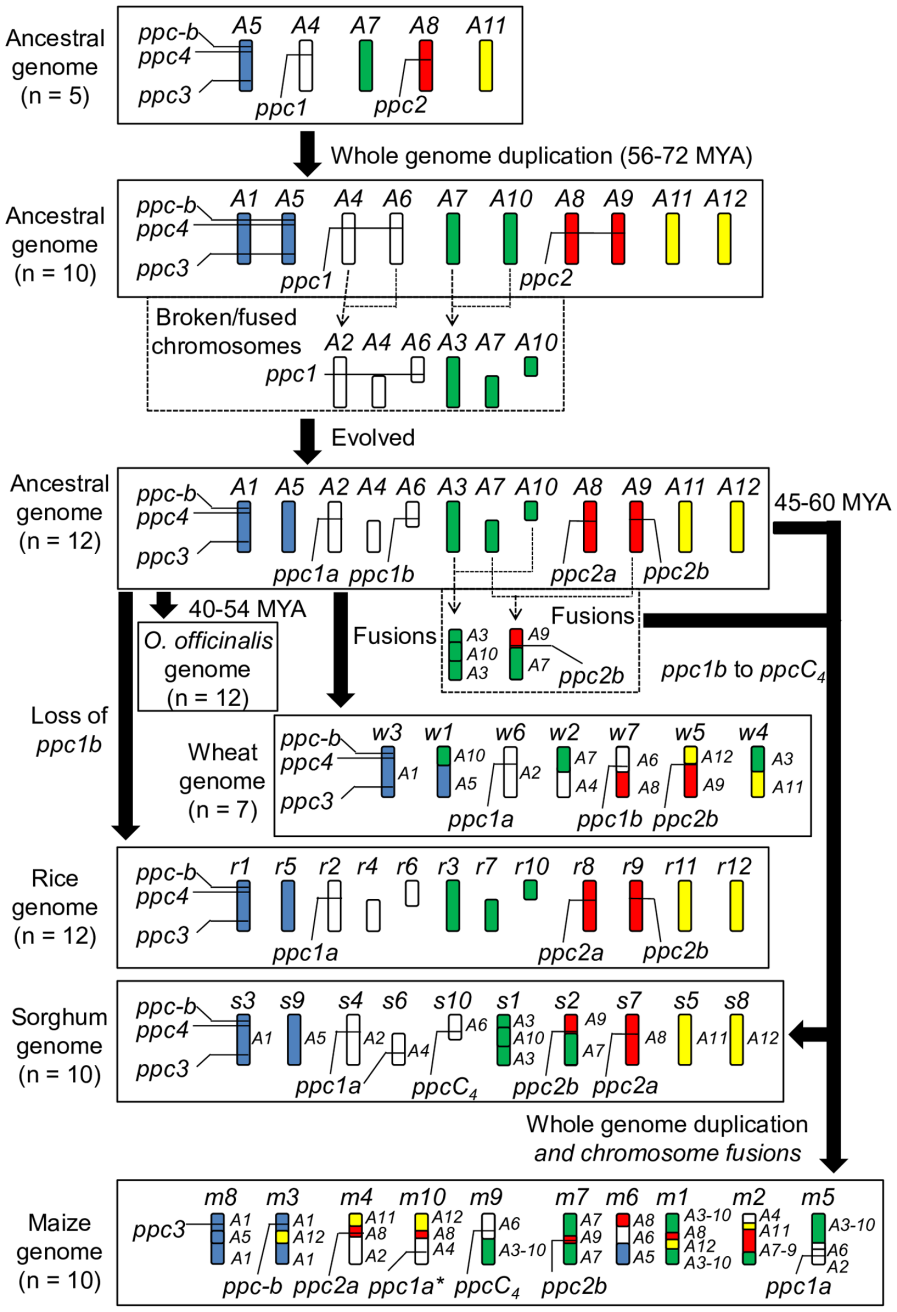
Inferred molecular inheritance of *PEPC* isogenes in the ancestral grass genome model. The molecular clock information was referred from a study by The International Brachypodium Initiative (2010). The present status of the rice genome, the *O. officinalis* genome, the wheat genome, the sorghum genome, and the maize genome were also shown. The asterisk represents “pseudogene.”

To seek genome-level evidence for the molecular evolution of grass *PEPCs*, we referred to the structural genome evolution model of Salse et al. (2008). It allowed inference of all the evolutionary origins and processes of PEPCs in rice, a wild rice *O. officinalis*, wheat, sorghum, and maize (Figure 3). Originally there were five ancestral genome blocks A4, A5, A7, A8, and A11, and A4 encodes *ppc1*, A5 encodes *ppc3*, *ppc4*, and *ppc-b*, and A8 encodes *ppc2*. Theoretically, the whole-genome duplication at around ~90 million years ago (MYA) increased the copies of each *PEPC* isogene group, resulting in a paralogous pair of *ppc1* (*ppc1a* and *ppc1b* on A4 and A6, respectively) and *ppc2* (*ppc2a* and *ppc2b* on A8 and A9, respectively). Following chromosomal breakages and fusions established a set of 12 ancestral grass chromosomes: A1–A12, among which A1 retains *ppc3*, *ppc4*, and *ppc-b*, A8 and A9 retain *ppc2a* and *ppc2b*, respectively, and A2 and A6 retain *ppc1a* and *ppc1b*, respectively. In this step, we assumed that the duplicated isogenes for ppc3, ppc4, and ppc-b were lost due to the redundancy. The 12 ancestral grass chromosomes with the chromosomal localizations of these *PEPC* isogenes are identical to *O. officinalis*. During the evolution of *Oryza* species, *ppc1b* was lost, and the *PEPC* gene composition of the cultivated rice was established; it was supported by the formation of *ppc1b* pseudogene on the corresponding locus in the BB genome-type wild rice *O. punctata* (Supplementary Figure S6). All the *PEPC* isogenes on the ancestral grass chromosome were inherited into the wheat A, B, and D genomes after multiple chromosomal recombination events. The genomic localization of these *PEPC* isogenes was typical among the wheat species (Supplementary Figure S7). These *PEPC* isogenes were retained on the genome of the Pooid grass model *B. distachyon* on the expected chromosomal segments even though its genome was nested (The International Brachypodium Initiative, 2010; Supplementary Figure S8). Regarding the C_4_-photosynthetic grass species sorghum and maize, the *PEPC* isogene composition of the common ancestral chromosomes might differ from the C_3_-photosynthetic grass species; namely, *ppc4* was absent from A1, A4 maintains a copy of *ppc1a* or a pseudogene of *ppc1a*, and *ppc1b* on A6 evolved into *ppcC_4_*. These differences may have occurred in the recent 45–60 million years.

We noted that an alternative wild rice species *O. meyeriana* maintains *ppc1b*. To check the molecular evolution of *ppc1b*, we searched it for 15 *Oryza* species and the outgroup *L. perrieri* at the genome level using TBLASTN. It appeared that *ppc1b* sequence was absent from the genome assemblies of all the AA genome species analyzed (Supplementary Figure S9). The genomes having ppc1b were CC-type, GG-type, and KKLL-type ones. These results indicate that *ppc1b* was maintained until the recent evolution of *Oryza* genomes but lost in multiple sub-lineages independently. According to the example in *O. punctata* (Supplementary Figure S6), gene loss of *ppc1b* is likely to occur not due to chromosomal-level events but to locus-level events. We also searched *ppc1b* from other related species in *Panicoideae* to observe the absence of *ppc1b* (Supplementary Table S5).

**Figure 4:**
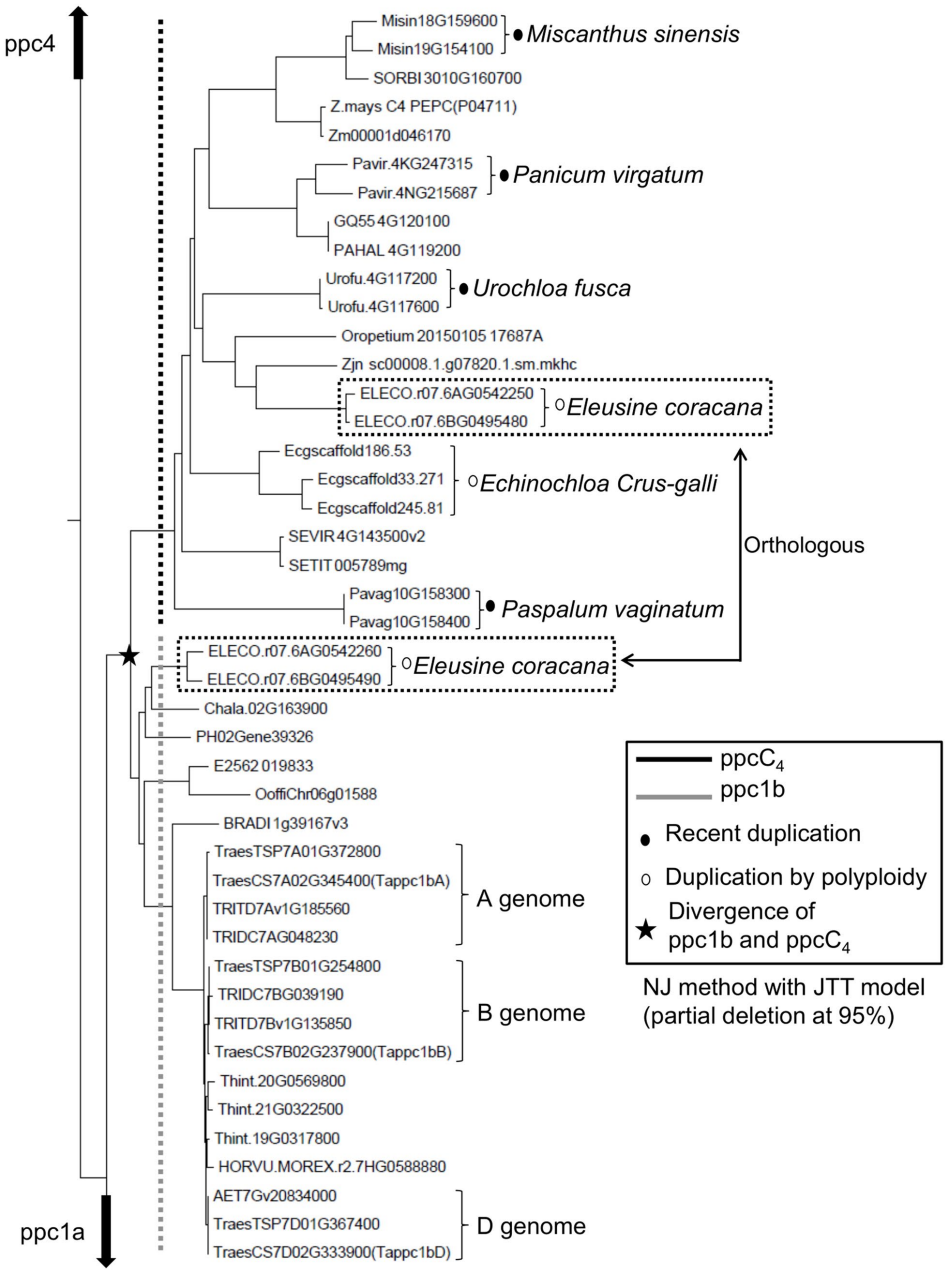
A partial NJ tree represents the orthologous relationship between ppc1b and ppcC_4_.

**Figure 5:**
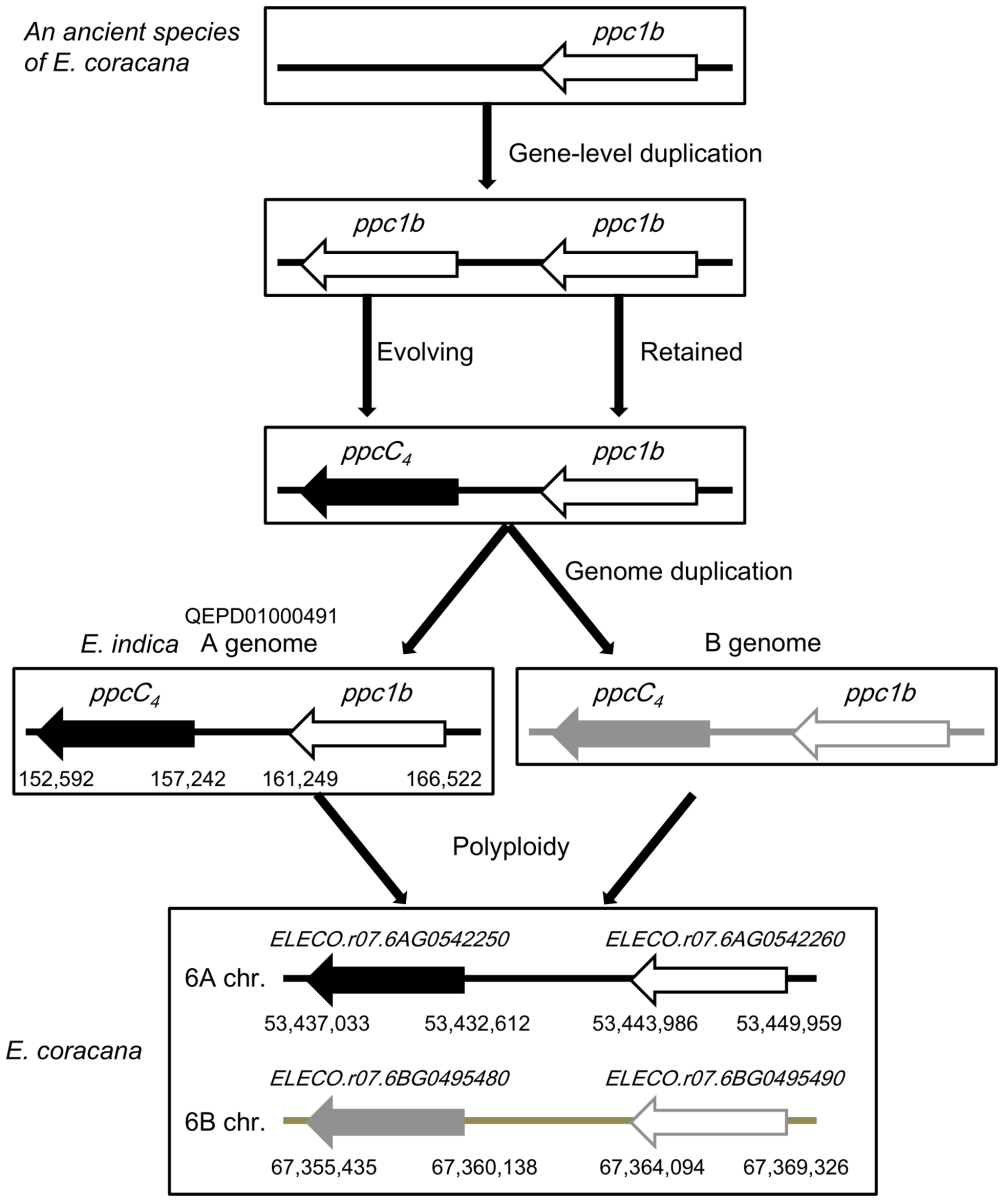
An evolutionary model for *ppc1b* and *ppcC_4_* in *Eleusine coracana*.

To evaluate the phylogenetic relationship and molecular evolutionary status of ppc1b and ppcC_4_, we constructed a phylogenetic tree using additional genome data sets (Supplementary Table S5). Same with the phylogeny mentioned above, it represented that ppcC_4_ formed only one lineage and looked derived from the common ancestor with ppc1b (Figure 4). Here, we found additional evidence that ppc1b is the molecular origin of ppcC_4_. Namely, the C_4_ cereal species *Eleusine coracana* (finger millet), which is considered to be abiotic stress tolerant (Gupta et al., 2017), has both *ppc1b* and *ppcC_4_* on the two types of allotetraploid genomes. Notably, the *ppc1b* in *E. coracana* is located next to the *ppcC_4_* isogene on each type of sub-genome. In addition, the A-genome donor *E. indica* maintained both *ppcC_4_* and *ppc1b* in the corresponding locus. These results indicate that these two *PEPC isogenes* were duplicated in the ancestral locus of a C_3_-photosynthetic species, and either of the gene copies evolved into *ppcC_4_* (Figure 5). More importantly, we observed three types of recent duplication of *ppcC_4_* in panicoid C_4_ grasses. Gene duplication of *ppcC_4_* by multiplication looked occurred in *Panicum virgatum* and *Echinochloa crus-galli*. Gene duplication of *ppcC_4_* at the locus level was seen in *Paspalum vaginatum* and *Urochloa fusca*. *Miscanthus sinensis* caused recent duplication of *ppcC_4_* in a different chromosome. These recent duplications of *ppcC_4_* suggest the physiological benefits of C_4_-photosynthetic PEPC.

### Predicted Origins of the Ancient Grass PEPCs

**Figure 6:**
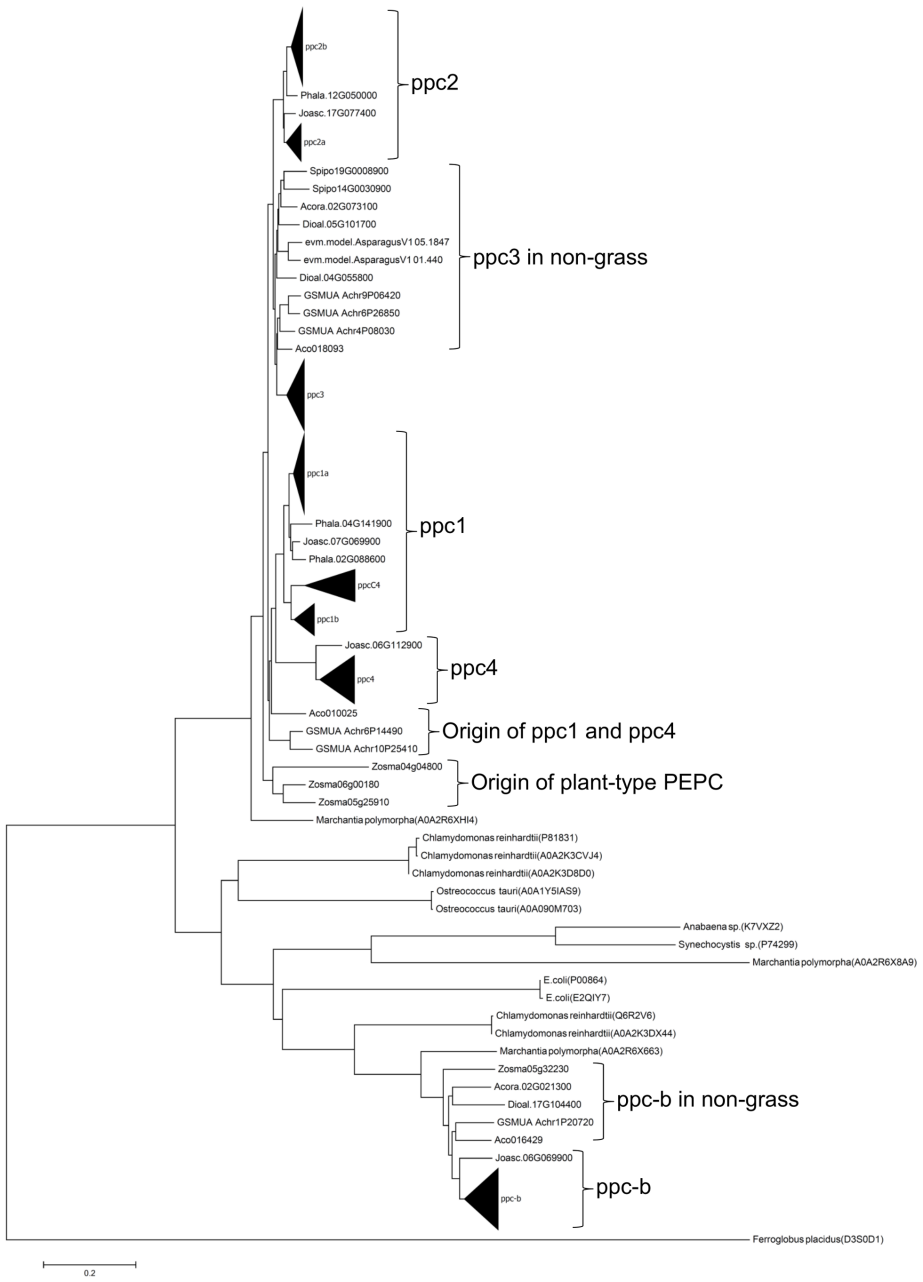
A NJ phylogenetic tree with JTT model for monocot PEPCs. Key PEPC protein identifiers are follows: Joasc.17G077400, Joasc.09G001100, Joasc.06G112900, and Joasc.07G069900 (*Joinvillea ascendens*), Phala.12G050000, Phala.04G0141900, and Phala.02G088600 (*Pharus latifolius*), Spipo19G0008900 and Spipo14G0030900 (*Spirodela polyrhiza*), Acora.02G073100 and Acora.02G021300 (*Acorus americanus*), Aco018093, Aco010025, and Aco016429 (*Ananas comosus*), Dioal.05G101700, Dioal.04G055800, and Dioal.17G104400 (*Dioscorea alata*), evm.model.AsparagusV1 05.1847 and evm.model.AsparagusV1 01.440 (*Asparagus officinalis*), GSMUA Achr9P06420, GSMUA Achr6P26850, GSMUA Achr4P08030, GSMUA Achr6P14490, GSMUA Achr10P25410, and GSMUA Achr1P20720 (*Musa acuminata*), Zosma04g04800, Zosma06g00180, Zosma05g25910, and Zosma05g32230 (*Zostera marina*). Black triangles represent sub-lineages of PEPC types. The bar shows the branch length of 0.2.

The origins of the ancient grass PEPCs were predicted based on a phylogenetic tree of monocot PEPCs (Figure 6). The ppc-b group in non-grass monocot species formed one lineage with grass species. Hence ppc-b in grass species would have come from the common ancestral monocot species. The ppc1 group and ppc4 group seemed to originate from a common ancestral lineage, which corresponded to a cytosolic *Acorus americanus* PEPC (Aco010025), implying that the chloroplast-targeting of ppc4 was given after the duplication of ppc1 and ppc4. Since *Joinvillea ascendens* has the orthologues of ppc1 (Joasc.07G069900) and ppc4 (Joasc.06G112900), these two PEPC lineages were formed in the early stage of grass evolution perhaps. The ppc3 group formed one lineage, suggesting that ppc3 originated from one ancestral lineage corresponding to *A. americanus* PEPC (Aco018093). The origin of the ppc2a and ppc2b group was unclear because no orthologue in non-grass monocot species was found in our analysis. The marine monocot species *Zostera marina* has three PEPC isoforms (Zosma04g04800, Zosma05g25910, and Zosma06g00180), which are located on the upstream branch of the five plant-type PEPCs in land monocot plants, implying that the formation of plant-type grass PEPCs was dependent on the atmospheric environment during evolution.

### Structural Differences of Grass PEPC Groups

Fixed amino acid substitutions would play essential roles in the biochemical properties of PEPCs. Muramatsu et al. (2015) reported that biochemical properties of plant-type PEPCs in rice differed from each other. We searched conserved amino acid sequences in each PEPC group but differed from any other PEPC group across the grass PEPCs. A total of 45 fixed amino acid substitutions were found in either ppc4, ppcC_4_, or ppc-b (Supplementary Figure S10). The bacterial PEPCs have 41 distinct amino acid positions, and ppc4 and ppcC_4_ have three and two unique amino acid substitutions, respectively. The unique substitutions for ppc4 are R_122_K, E_185_Q, and Q_222_L, whose biochemical functions are unknown (Supplementary Figure S11). The distinct amino acid substitutions for ppcC_4_ were A_531_P and A_780_S (Supplementary Figure S11). The former amino acid substitution was reported as a positive selection site in C_4_-photosynthetic PEPCs (Christin et al., 2007), and the latter amino acid substitution in ppcC_4_ is well-known as the leading cause of the advanced biochemical property of C_4_-photosynthetic PEPCs (Bläsing et al., 2000). For other plant-type PEPCs, we found no unique amino acid substitutions for each group. These results implicate that unfixed amino acid substitution sites in particular PEPC groups involve the diverged biochemical properties of non-photosynthetic plant-type PEPCs. We could find 34 fixed substitutions between ppc2b and ppc4. Regarding ppc1a and ppc2b, 18 fixed substitution sites were found. Seven amino acid substitutions (V_42_L, S_100_K, G_155_K, Q_364_K, R_495_N, M_717_L, and S_753_L) largely dissected two evolutionary lineages between ppc4/ppc1a/ppc1b and ppc3/ppc2a/ppc2b (Supplementary Figure S11). S_100_K, Q_364_K, and R_495_N, which need more than one nucleotide substitution and bring changes of amino acid characteristics, may be a hint of the evolution of wheat PEPCs in biochemical properties. The functionality of S_100_K in allosteric regulation of maize C_4_-photosynthetic PEPC was reported by González-Segura et al. (2018), while other determinants of the C_4_-photosynthetic PEPC characteristics in the range of the amino acids 296–437, which corresponds to 302–443 in maize C_4_-photosynthetic PEPC (Engelmann et al., 2002) remain to be unknown. The substitution Q_364_K, located on the plant-specific sequence, is a potential candidate site.

### Positive Selection of ppc1b

**Figure 7:**
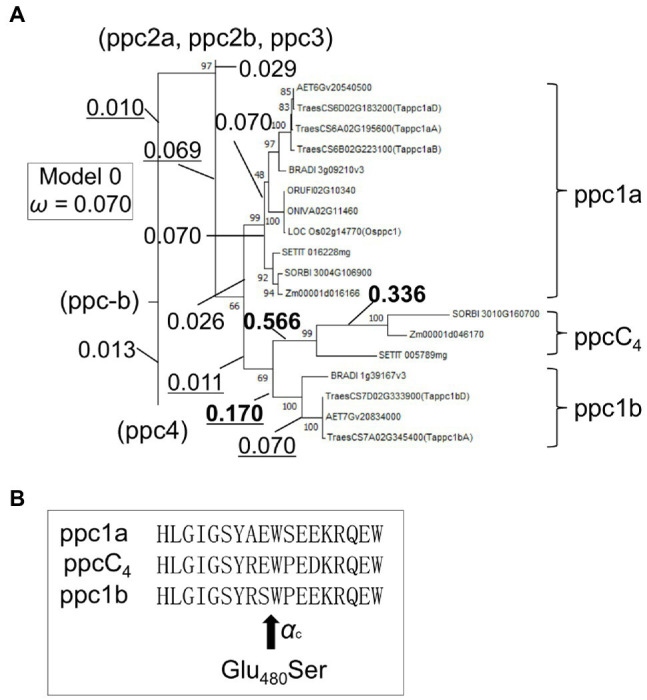
Detected positive selection in the ppc1b and ppcC_4_ lineages. **(A)** A partial phylogenetic tree showing *ω* values of branches. The *ω* values of the ppc1b lineage were underlined. Bold *ω* values represent statistical significance. **(B)** The positive selection site in the ppc1b branch. The amino acid positions are maize C_4_ PEPC coordinate’s. *α*_c_ represents the physicochemical amino acid property related to the substitution.

The accelerated molecular evolution of ppc1b after diversifying from the ppc1a lineage implicates the occurrence of natural selection. We could mine 11 potential positive selection sites in plant-type wheat PEPCs (Supplementary Table S6). These substitutions were associated with seven classes of physicochemical amino acid properties such as “Polarity” (*p*), “Equilibrium constant (ionization of COOH)” (*pK’*), and “Power to be at the C-terminal, α-helix” (*α*_c_), used by the tool TreeSAAP. Then, using the methods implemented in CodeML, we detected positive selection in the ppc1b branch after divergence from ppcC_4_ (Figure 7A). Branch-site model search revealed one positive selection site (Glu_480_Ser) for the ppc1b branch with statistical significance (5% level; Figure 7B). This substitution corresponded to the physicochemical property of *α*_c_, implicating biochemical relevance. Branch-site model search for ppcC_4_ detected three positive selection sites (A_243_Y), (R_560_P), and (H_679_F) after the divergence from the ppc1b lineage with statistical significance, being consistent with our knowledge that the present forms of C_4_-photosynthetic PEPC were made by adaptive evolution. Two of these sites (A_243_Y) and (H_679_F) were not found by Christin et al. (2007).

### Gene Expression Characteristics of PEPC Isogenes in Wheat and Other Grass Models

The expression levels for the wheat *PEPC* isogenes in public RNA-Seq profiles showed divergent spatial expression patterns (Supplementary Figures S12, S13). *Tappc1a* isogenes exhibited preferential expressions in roots and reproductive organs. *Tappc1b* isogenes represented high-level expressions in the leaf sample at a seedling stage. *Tappc2* isogenes showed ubiquitous expression patterns, suggesting their functions are housekeeping. *Tappc3* isogenes exhibited preferential expressions in reproductive organs, including ovaries. *Tappc4* showed preferential expressions in leaf samples. Gene expressions of *Tappc-b* isogenes were undetectable in many samples, while low-level expressions were observed in reproductive organs such as anther, ovary, and spike. Overall, the orthologous *PEPC* isogenes were generally expressed in the same manner (Supplementary Figures S12, S13), suggesting that the gene regulation of the orthologous *PEPC* isogenes in the three types of genomes were conserved among each other.

**Figure 8:**
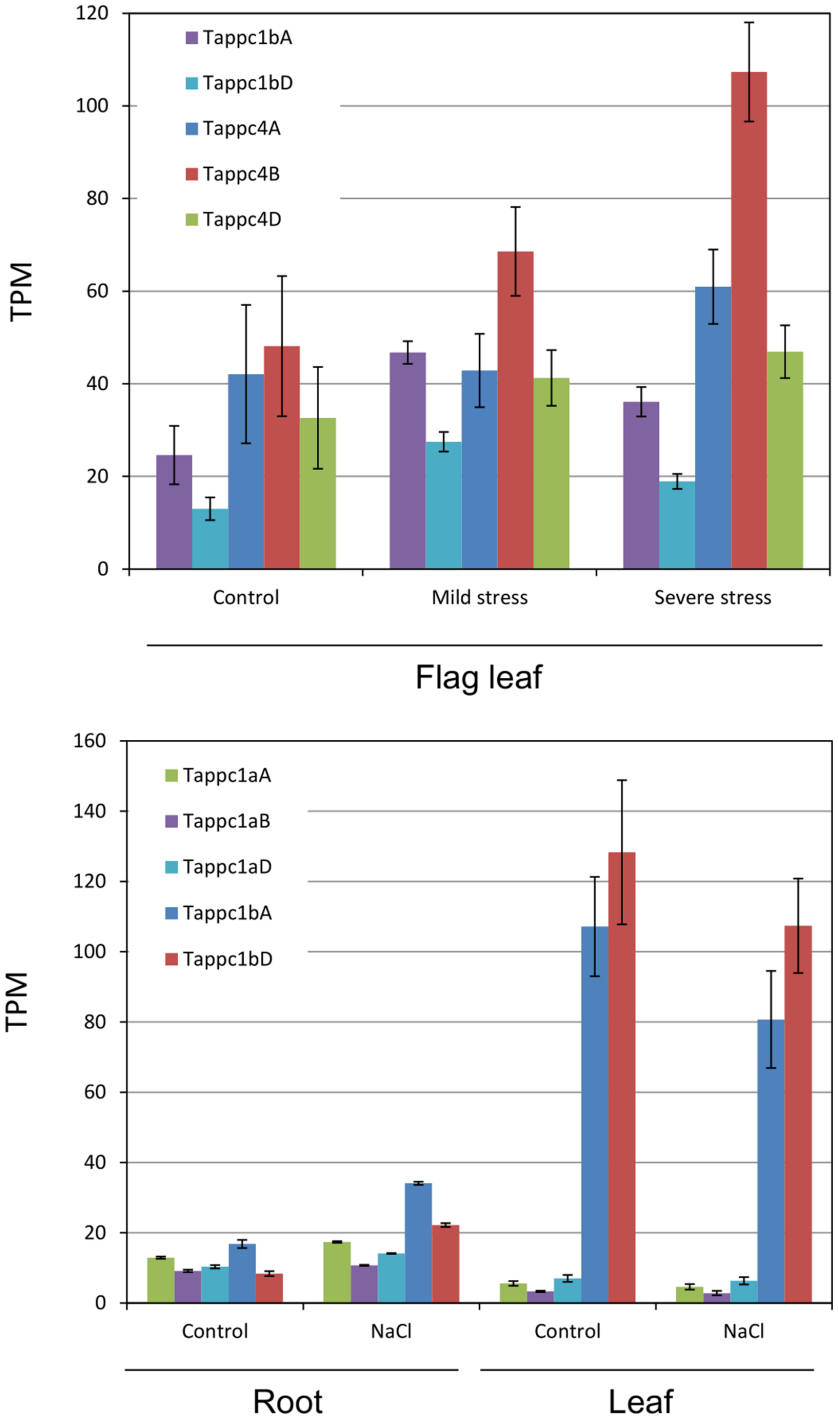
Abiotic stress responses of *Tappc1a*, *Tappc1b*, and *Tappc4* isogenes. Asterisks represent statistical significance at 5% level in Student’s *t*-test. **(A)** Drought stress responses in flag leaves, **(B)** salt stress (100 mM NaCl) responses in roots and leaves.

By compilation of public RNA sequencing profiles under three types of abiotic stress and nitrogen stress, we observed varied responses of wheat *PEPC* isogenes. Under drought stress conditions, *Tappc1b* and *Tappc4* isogenes showed upregulation in flag leaves, while *Tappc2* isogenes showed down-regulation (Figure 8; Supplementary Table S7). Other *PEPC* isogenes showed no noticeable transcriptional changes under the conditions. Under a salt stress condition, *Tappc1a* isogenes and *Tappc1b* isogenes exhibited upregulation in roots but downregulation in leaves. The salt stress decreased the gene expression levels of *Tappc2* isogenes and *Tappc4* isogenes. These results suggest that *Tappc1b* isogenes is responsible for osmotic stress adaptation. By contrast, under a heat stress condition, transcriptions of *Tappc1a*, *Tappc1b*, *Tappc2*, and *Tappc4* isogenes were downregulated. Since the gene expression levels of *Tappc3* and *Tappc-b* isogenes were low, we could not monitor the effects of the abiotic stress. Regarding nitrogen stress, we observed that nitrogen availability differently affected gene expression levels of *PEPC* isogenes in wheat organs (Supplementary Tables S8, S9). Gene expression levels of *Tappc1b* isogenes showed apparent increases in shoots under sufficient nitrogen conditions while decreases in roots. These results indicate that Tappc1b is under organ type-dependent different gene regulations.

To know details of the transcriptional regulation of *PEPC* isogenes, public RNA-Seq profiles in other grass model plants were analyzed. *Brachypodium* data indicated light-dependent and salt stress-induced gene regulation of *ppc1b* (Supplementary Tables S10, S11). Barley data in the aerial part represented potential nitrate-dependent transcriptional enhancement of ppc1b dependent on nitrate supplement (Supplementary Table S12). RNA-Seq profiles in *T. turgidum* indicated selective transcriptional enhancement of *ppc1b* isogenes in leaf-stem samples and spikelets (Supplementary Table S13). For the C_4_-photosynthetic cereals, sorghum and maize, no clear expression changes of *PEPC* isogenes were observed under nitrogen stress conditions (Supplementary Tables S12–S15). The leaf RNA-Seq profiles in *E. coracana*, of which genome maintains *ppcC4* and *ppc1b* both, represented distinct differences at the gene expression level between *ppcC4* and *ppc1b* (Supplementary Table S16), confirming their molecular types and physiological roles are different. Overall, the gene expression patterns of *ppc1b* differed from other types of *PEPC* isogenes.

### Selective Transcriptional Regulation of *Tappc1b*, *Tappc2*, and *Tappc4* in Response to NO_3_^−^

**Figure 9:**
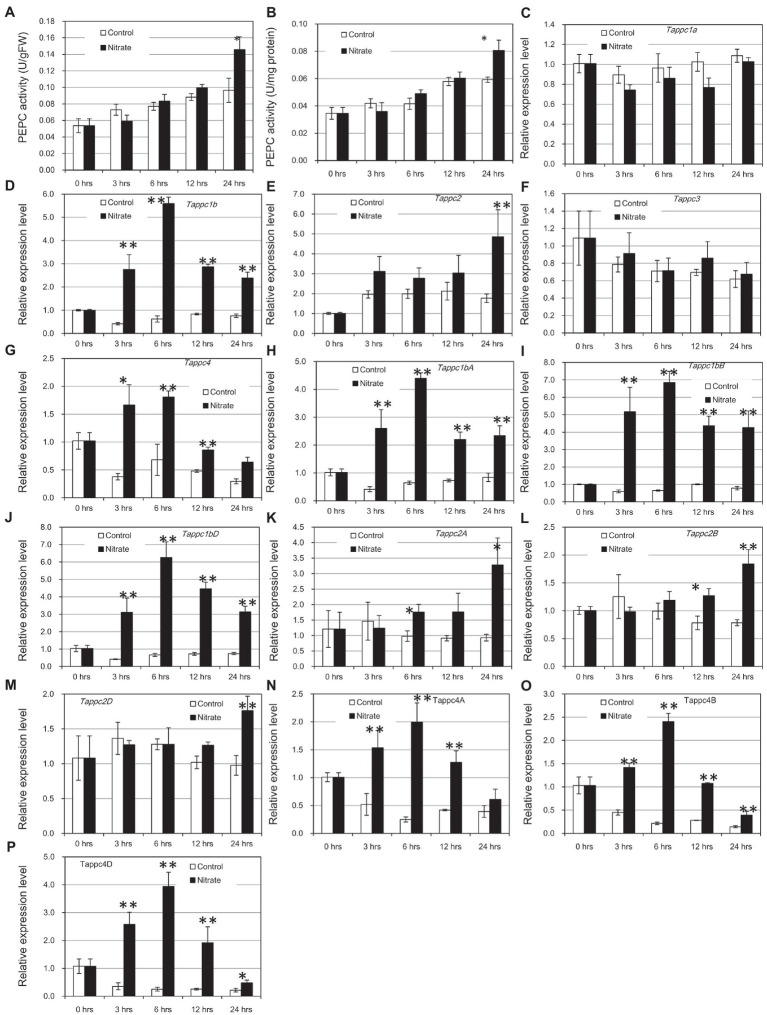
Enzyme activity and gene expressions of PEPCs in response to nitrate. Error bars indicate SEs. Double and single asterisks indicate statistical significance at 5 and 10% level, respectively. **(A)** PEPC activity per g fresh weight, **(B)** PEPC activity per mg protein, **(C–G)** Relative expression levels of wheat *PEPC* isogene groups, and **(H–P)** Relative expression levels of *Tappc1b*, *Tappc2*, and *Tappc4* isogenes in the A, B, and D genomes.

To identify the biologically significant response of PEPC to nitrogen supply, we prepared 2-week old wheat seedlings under a nitrogen-deficient condition and supplied 40 mM NO_3_^−^ to detached leaves from these plants. As expected, the supplement of NO_3_^−^ increased PEPC activity according to the incubation time. PEPC activity showed significant upregulation at 24 h after incubation compared to the mock condition (at 5% level in Student’s *t*-test; Figures 9A,B). The patterns of PEPC activity were very similar in two ways of measurements: fresh weight-basis and soluble protein amount-basis. The increases of PEPC activity after the treatment in control and NO_3_^−^-treated samples were observed due to higher strength of light provision after the detachment physical stress of leaf detachment. Thus, the up-regulation of PEPC activity in leaves under the supplement of NO_3_^−^ was not acute but not long-periodical in our condition. The concomitant increase of PEPC proteins at 24 h after the NO_3_^−^ treatment was confirmed by Western blotting (Supplementary Figure S14).

To check the transcriptional status of all the *PEPC* isogenic groups, we conducted qRT-PCR using a consensus primer set for each isogene group. The results indicated up-regulation of *Tappc1b*, *Tappc2*, and *Tappc4* groups by NO_3_^−^ (Figures 8C–G). Gene expression of the Tappc-b group was undetectable in our experimental condition. To verify NO_3_^−^ response at isogene level, we performed additional qRT-PCR assays using gene-specific primers designed on 3′UTR regions for *Tappc1b*, *Tappc2*, and *Tappc4* groups (Figures 8H–N). The most obvious response was observed for *Tappc1b* at 6 h after incubation, but significant upregulation at 3, 12, and 24 h after incubation was also observed. *Tappc4* exhibited upregulation with a lesser fold change than that of *Tappc1b*. In addition, *Tappc2* exhibited upregulation clearly after 24 h of induction, indicating the presence of an alternative mechanism of transcriptional upregulation of *PEPC*. The downregulation of *Tappc4* in the mock condition might be due to the effect of SO_4_^2−^ (Supplementary Figure S15).

### Candidate cis-Motifs for the Transcriptional Regulation of *Tappc1b* and *Tappc4* by NO_3_^−^

**Figure 10:**
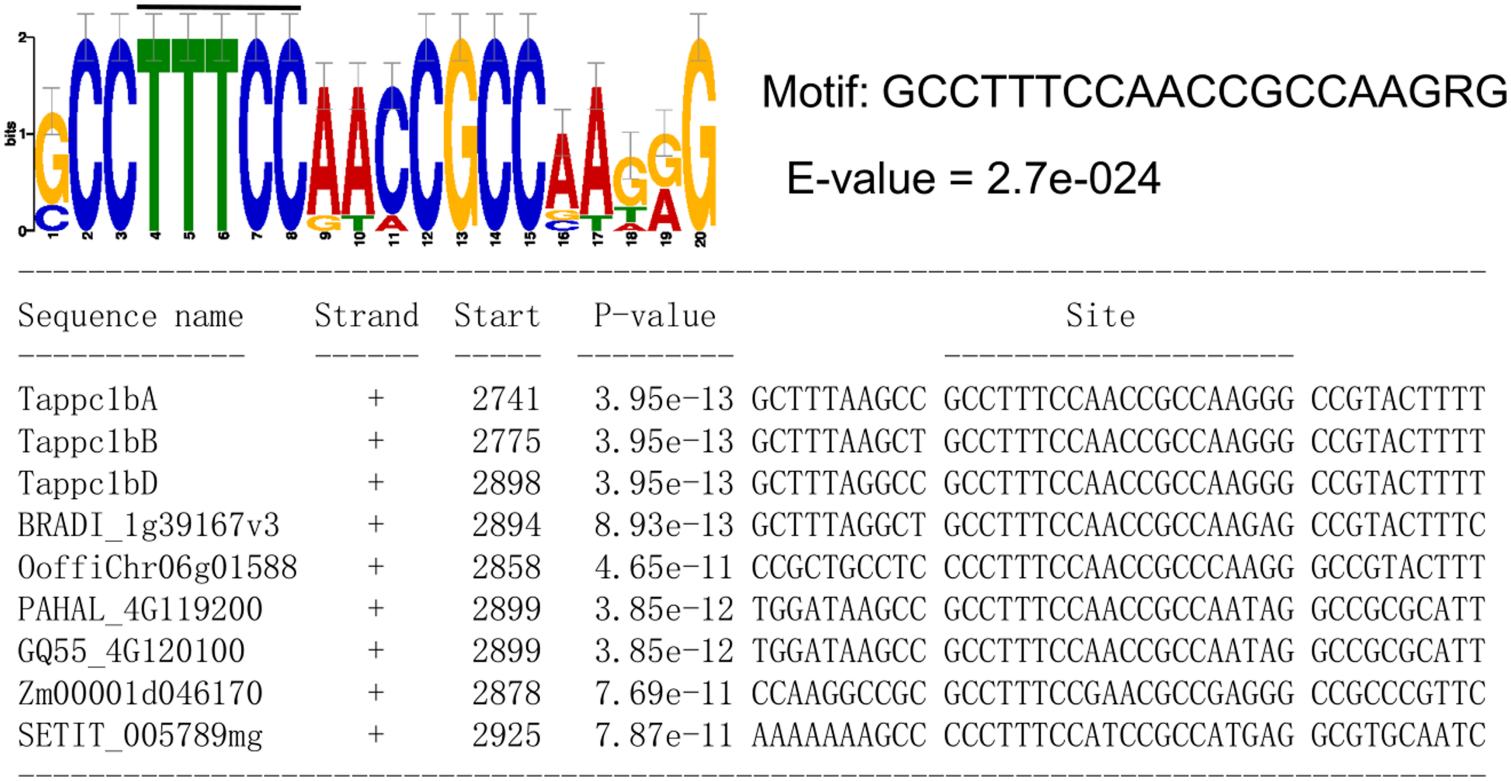
A predicted cis-motif associated with the nitrate responses of *Tappc1b* isogenes. The black bar above the motif logo represents the position of Dof1 binding core motif.

The NO_3_^−^ responses of *Tappc1b* and *Tappc4* in the exact timing imply that the same regulatory mechanism regulates these two *PEPC*s. To approach the regulatory mechanism of the transcriptional upregulation of *Tappc1b* and *Tappc4*, we conducted comparative motif searches in the promoter regions of these wheat *PEPC* isogenes and the orthologous isogenes with *Tappc1b*, including *ppcC_4_*. We could predict a cis-motif (GCCTTTCCAACCGCCAAGRG), which are from *Tappc1b* and *ppcC_4_* (Figure 10). Notably, the motif contained a similar sequence with the transcription factor Dof1 binding core motif “AAAGG.” This result was consistent with that Dof1 possibly regulates plant PEPCs (Yanagisawa, 2000). In addition, it contained a Dof1 core region bound to Dof1 protein in a gel-shift assay (Yanagisawa and Sheen, 1998), suggesting Dof1-mediated transcriptional regulation of wheat *PEPC* isogenes. In addition, seven cis-motif candidates were predicted (Supplementary Figure S16). The motif (SGGCTGKGGCCWGTGVTGRGSG) includes a candidate nitrate-response element found by Pathak et al. (2009).

## Discussion

To investigate the evolutionary processes of grass PEPCs, we analyzed *PEPC* isogene compositions in several grass species, including wheat, rice, maize, and sorghum. The plant-type PEPCs in grass species seem to be originated from one ancient lineage and formed five ancestral PEPCs, followed by dynamic molecular evolution with the whole-genome duplication in the early period of grass species divergence (around ~90 MYA) Huang et al. (2022). We discovered a new non-photosynthetic *PEPC* group ppc1b retained in particular grass genomes. It is likely to be the primary molecular origin of grass C_4_-photosynthetic PEPCs. The explicit designation of ppc1b allowed a clear illustration of the evolutionary processes of photosynthetic and non-photosynthetic PEPCs during evolution. Consistent with the phylogenetic analysis result using partial *PEPC* sequences by Christin et al. (2007), C_3_-photosynthetic *Pooideae*, including *Triticum* species, retain ppc1b. We detected *ppc1b* sequences in the genome assemblies of non-*Triticum* Pooids *Avena eriantha*, *Dactylis glomerata*, *Lolium multiflorium*, *L. perenne*, and *Puccinellia tenuiflora* in addition to *B. distachyon* (data not shown). By contrast, many species in the *Oryza* lineage have lost ppc1b during evolution. These facts support that PEPC is highly associated with grass species diversity.

Characterization of *ppc1b* revealed its molecular plasticity and similarity with C_4_-photosynthetic PEPCs. Namely, ppc1b was subjected to adaptive evolution after the divergence from ppcC_4_ and exhibited abiotic stress-relation in wheat and *B. distachyon* (Supplementary Figure S17). In addition, *ppc1b* in wheat maintains the regulatory characteristics of maize *ppcC_4_*: nitrogen-dependent transcription in photosynthetic organs (Sugiharto and Sugiyama, 1992; Suzuki et al., 1994) and abiotic stress responses (Supplementary Figures S17, S18). Our results indicated that wheat *ppc1b* is potentially regulated by Dof1, a nitrogen metabolism-associated transcription factor for *ppcC_4_* in maize. Meanwhile, the protein sequences of Tappc1b isoforms are likely to be of plant-type PEPC in C_3_-photosynthetic plants (Supplementary Figure S5). We suppose that *ppc1b* and *ppcC_4_* are representative genes that evolved with the functional diversification of grass species.

In the evolutionary story of grass C_4_-type PEPCs by Christin and Besnard (2009), the divergence of C_4_- photosynthetic PEPCs and non-photosynthetic PEPCs occurred before the divergence of C_4_ and C_3_ plants. Christin et al. (2007) indicated that C_4_- photosynthetic and non-photosynthetic PEPCs are distinguished based on the conserved motifs specific in C_4_-photosynthetic PEPC isoforms of a few distant polypeptide sites. Meanwhile, C_4_ plants had occurred in several evolutionary branches of monocot species. Hence, this story is hardly understood with ease unless convergent molecular evolution of PEPC or lateral transfer of C_4_-photosynthetic PEPC is accepted, even though their analysis suggests the PEPC class ppc-B2 is highly associated with the C_4_-photosynthetic PEPCs in grass species. Our integrative approach using grass genome data clarified that all the C_4_-photosynthetic PEPCs analyzed were derived from the common ancestor with ppc1b. The unique C_4_ grass species *E. coracana*, which has *ppc1b* and *ppcC_4_* on the two types of allotetraploid genomes, represented the direct link between ppc1 and ppcC_4_. According to Christin et al. (2007), there seem to be several *Chloridoideae* species that retain *ppc1b* and *ppcC_4_*. Further genome-level analyses should reveal other evidence for the origin of C_4_-photosynthetic PEPCs.

One of the most crucial functions of plant PEPCs is an adaptation to the environment. C_4_-photosynthetic PEPCs were probably born to adapt to high-temperature conditions. Plant cells require substantial energy and biosynthesis of osmolytes such as proline (Pan et al., 2021). However, this stress represses photosynthetic activities, resulting in high energy demand. Here, PEPC could act on re-fixing carbon released by respiration to complement the limited carbon source. This fact might be one of the reasons why C_4_-photosynthetic species generally exhibit high abiotic stress tolerance (Pardo and VanBuren, 2021). Hence, we hypothesize that ppc1b and ppcC_4_ have evolved under substantial environmental stress. Retention of *ppc1b* in wheat species suggests its physiological benefits under osmotic stress. Typically, the Triticum-Aegilops complex is capable of growing well under semi-arid conditions. By contrast, cultivated rice prefers more wet conditions. For example, one of the *Oryza* species retaining ppc1b, *O. coarctata*, exhibits high salt tolerance (Chowrasia et al., 2018), while cultivated rice is well-known for its less tolerance to salt stress.

Gene loss of *ppc1b* during the evolution of *Oryza* species implicates the functional neutrality of ppc1b for these *Oryza* species. Otherwise, ppc1b was lost in cultivated rice due to the functional redundancy with other *PEPC* isogene. The former possibility can be explained by an ecologically different background between the *Oryza* species and *Pooideae*. Pooid cereals typically exhibit superior abiotic stress resistance, especially salt and drought stress. The latter possibility can be explained by an additional copy of *ppc2* (*ppc2a*) in the *Oryza* species genomes. We observed selective drought stress response of *ppc2b* in rice public microarray data (Supplementary Figure S19). The presence of *ppc2a* in the *Oryza* genomes might have allowed the evolutionary adaptation of *ppc2b* to cope with the lack of *ppc1b*.

Response to nitrogen is one of the primary behaviors of plant PEPCs (Sugiharto et al., 1990; Zhang et al., 2012; Yamamoto et al., 2014b, 2017). Two proposed mechanisms explain the increased PEPC activity by nitrogen supply: transcriptional level and post-translational level regulations (Sugiharto et al., 1990; Sugiharto and Sugiyama, 1992; Wu and Wedding, 1992). Duff and Chollet (1995) applied a test system where the basal condition has a low concentration of NO_3_^−^ and observed post-translational regulation of PEPC by *in vivo* phosphorylation. To identify NO_3_^−^-dependent response, we employed a detached leaf test system, where a high concentration of NO_3_^−^ is supplied to a nitrogen-deficient condition. Since NO_3_^−^ could be a signal of gene transcription (Sugiharto and Sugiyama, 1992; Dechorgnat et al., 2011; Medici and Krouk, 2014; Vidal et al., 2020), we assumed that our system would be more suitable than other systems applied in the previous studies. Under the limited availability of nitrogen, the gene expressions of *Tappc1b* and *Tappc4* groups were maintained at a low level. Once NO_3_^−^ was given, the expressions of these genes were induced quickly, i.e., within 3 h. We assume that the transcriptional induction of *Tappc1b* and *Tappc4* orthologues in wheat would not be the indirect response to NO_3_^−^, while *Tappc2* orthologues made a late transcriptional response in concordant with the increased PEPC activity. These three *PEPC* isogene groups could underlie the increased PEPC activity in the detached leaves, but the different expression manners might imply different roles in nitrate-related metabolism. We wonder if *Tappc1b* acts on balancing nitrogen-carbon metabolism in *Pooideae*.

## Conclusion

We discovered a new PEPC isoform group ppc1b by the cross-species genome-wide analysis in Poaceae. PEPC isoforms belong to ppc1b would be the origin of grass C_4_-photosynthetic PEPCs. Those maintain similar characteristics to those of C_4_-photosynthetic PEPCs but are likely to play role in non-photosynthetic physiology, i.e., abiotic stress adaptation. Our results clearly indicated that C_4_-grass species genomes lack *ppc1b* due to the evolutionary change of ppc1b into C_4_-photosynthetic PEPC. The presence of *ppc1b* in the *Eleucine* genomes implicates tandem gene duplication of *PEPC* might affect physiological characters of cereal species. To see whether utilization of non-photosynthetic *PEPC* in plant biotechnology are useful for enhancing abiotic stress tolerance in cereal crops or not, experimental evaluation using transgenic techniques is required.

## Data Availability Statement

The cDNA sequence data that we obtained in this study can be found in the Genbank online repositories. The accession numbers are ON055387, ON055388, and ON055389.

## Author Contributions

NY conducted computations, assisted experiments, analyzed the data, and wrote the manuscript. BL and WT performed experiments to analyze experimental data. In addition, ZY assisted experiments and data analyses to improve the manuscript. ZP and ZY financially supported this study. All authors contributed to the article and approved the submitted version.

## Funding

This work is supported by the National Natural Science Foundation of China [Grant No. 32060456], the Natural Science Foundation of Sichuan Province [Grant No. 2022NSFSC0160], and the Science Foundation of China West Normal University (No. 19E060).

## Conflict of Interest

The authors declare that the research was conducted in the absence of any commercial or financial relationships that could be construed as a potential conflict of interest.

## Publisher’s Note

All claims expressed in this article are solely those of the authors and do not necessarily represent those of their affiliated organizations, or those of the publisher, the editors and the reviewers. Any product that may be evaluated in this article, or claim that may be made by its manufacturer, is not guaranteed or endorsed by the publisher.

## Footnotes

^1^ http://www.genome.jp/kegg/

^2^ http://www.ncbi.nlm.nih.gov/sra

^3^ https://www.r-project.org/

## Supplementary Material

Supplementary Figure S1 Photographs of wheat seedlings used in the nitrate induction experiment. **(A)** Two-week old wheat seedlings grown in a laboratory. **(B)** Detached wheat leaves under incubation with K_2_SO_4_ solution and KNO_3_ solution at 0 h.

Supplementary Figure S2 Prokaryotic expression of a Tappc1b fragment in *E. coli*. **(A)** A partial sequence alignment of Tappc1b in the wheat genome (100% identical among each other). The expressed polypeptide fragment was shown. One of the conserved domain, which is important for PEPC enzyme catalysis, was underlined. **(B)** PEPC activity in *E. coli* BL21 (DE3) harboring pET28a and pET28a with the Tappc1bD fragment insert. Overnight culture of transformed *E. coli* were inoculated onto fresh LB medium, and IPTG was added when OD600 reached onto 0.5. Then, the bacterial cultures were incubated at 25°C for 5 h. *E. coli* cells were harvested to extract soluble proteins, and PEPC activities were measured. Asterisks indicate statistical significance between “Tappc1bD IPTG” and other samples at 5% level.

Supplementary Figure S3 A multiple sequence alignment of wheat PEPC proteins. Known functional motifs were marked.

Supplementary Figure S4 Distributions of branch lengths for wheat PEPCs. Rice PEPCs were analyzed as references.

Supplementary Figure S5 Phylogenetic trees using the ML and neighbor-joining method with different parameters. **(A,B)** The ML method, **(C–J)** the neighbor-joining method.

Supplementary Figure S6 Comparison of the ppc1b locus between *Oryza officinalis* and *Oryza punctata*. **(A)** Genomic structure of the ppc1b locus in *Oryza officinalis* and *O. punctata*. Genomic positions of these species were shown above genes, and identifiers of corresponding genes in *O. sativa* were shown under genes. **(B)** TBLASTN search of Tappc1bD against the *O. punctata* genome. The gene loss region in *O. punctata* was highlighted.

Supplementary Figure S7 Chromosomal localization of *ppc1b* on the wheat, and other related genomes.

Supplementary Figure S8 Chromosomal localization of *ppc1b* on the *Brachypodium* genome.

Supplementary Figure S9 An evolutionary chart of wild rice and cultivated rice represents the presence and absence of *ppc1b*.

Supplementary Figure S10 Amino acid sequence variation sites that are fixed in each PEPC group. The positions were referred to the maize C_4_-photosynthetic PEPC.

Supplementary Figure S11 List of amino acid substitution sites that are related to the molecular evolution of plant-type PEPC.

Supplementary Figure S12 Spatial gene expression patterns of wheat PEPC isogenes. **(A)** A bar chat represents estimated gene expression levels in various organs. **(B)** PCA biplot showing the interrelationship of *Tappc* isogenes at gene expression patterns. **(C)** Variance of principal components.

Supplementary Figure S13 A bar chart showing the compositions of quantitative gene expression levels of *PEPC* isogenes in wheat. The horizontal axis represents TPM values.

Supplementary Figure S14 Western blot analysis of PEPC in wheat detached leaves. The sample lane 1: Before detachment, lane 2: 24 h with K_2_SO_4_, lane 3: 24 h with KNO_3_. Polypeptides representing nearby apparent size of PEPCs were designated as PEPC proteins. Signal intensities of PEPC proteins were calculated using ImageJ. The asterisk indicates statistical significance between “Mock” and “KNO_3_“samples at 10% level.

Supplementary Figure S15 The effect of K_2_SO_4_ to gene expression of *Tappc4* in wheat detached leaves. Error bars indicate standard errors. The asterisk represents statistical significance at 5% level in Student’s *t*-test.

Supplementary Figure S16 Other potential cis-motif candidates that are associated with nitrate response.

Supplementary Figure S17 Gene expression patterns of maize C_4_-photosynthetic *PEPC* under abiotic stress conditions. The data were retrieved from qTeller (Woodhouse et al., 2021).

Supplementary Figure S18 Selective up-regulation of ppc1b in leaves of three-week-old *B. distachyon* seedlings under a salt stress condition by 200 mM NaCl for 24 h (GenBank SRA BioProject accession: PRJNA636626). Error bars indicate standard errors. The asterisk and double asterisk represent statistical significance at 10 and 5%, respectively.

Supplementary Figure S19 Rice microarray data represents selective transcriptional response of *Osppc2b* under drought stress conditions. The data were retrieved from OryzaExpress (Hamada et al., 2011). Error bars indicate standard errors.
