# Supplementary material for: The Original Form of C4-Photosynthetic Phosphoenolpyruvate Carboxylase Is Retained in Pooids but Lost in Rice
Source: Front Plant Sci. 2022 Jul 25;13:905894. doi: 10.3389/fpls.2022.905894 (PMC9358456; doi:10.3389/fpls.2022.905894)
Supplement: Supplementary Figure S1 — Photographs of wheat seedlings used in the nitrate induction experiment. (A) Two-week old wheat seedlings grown in a laboratory. (B) Detached wheat leaves under incubation with K2SO4 solution and KNO3 solution at 0 h. [file Presentation_1.pdf]

A

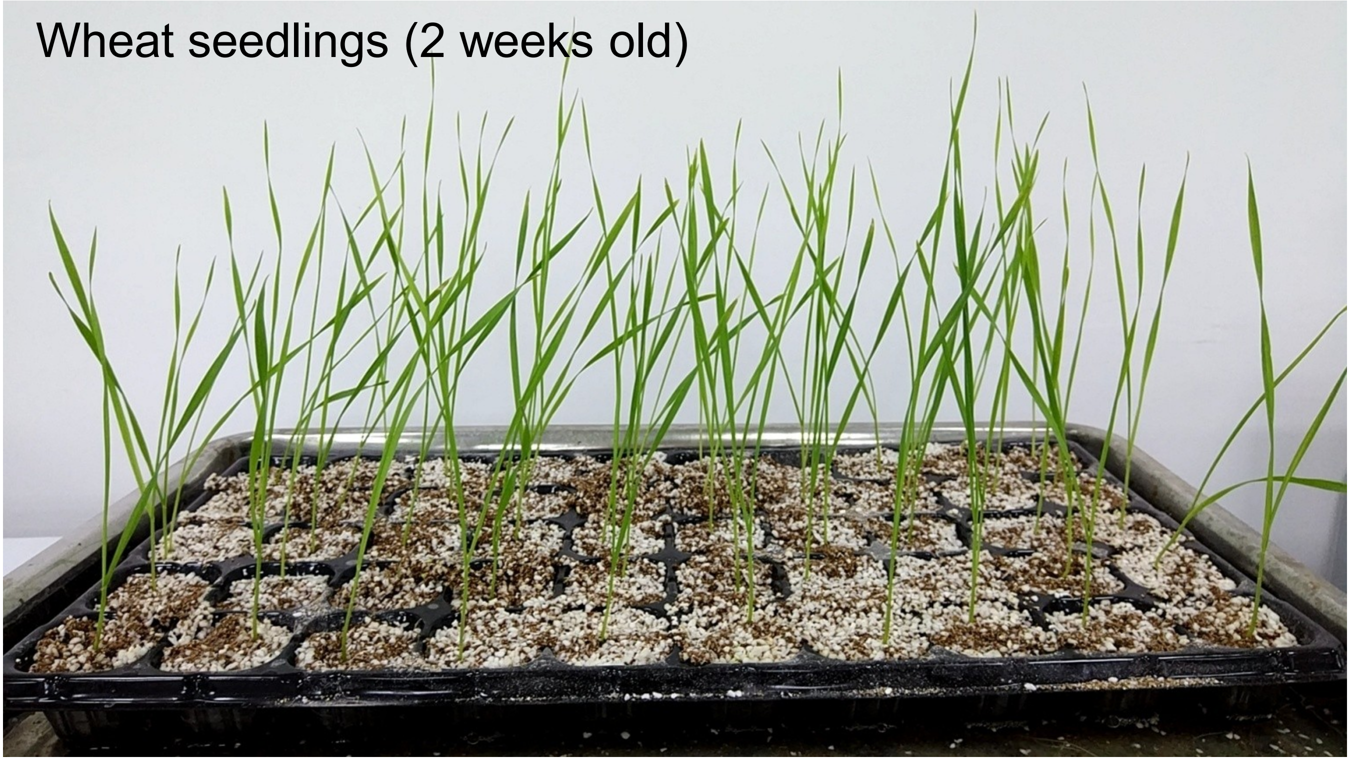

B

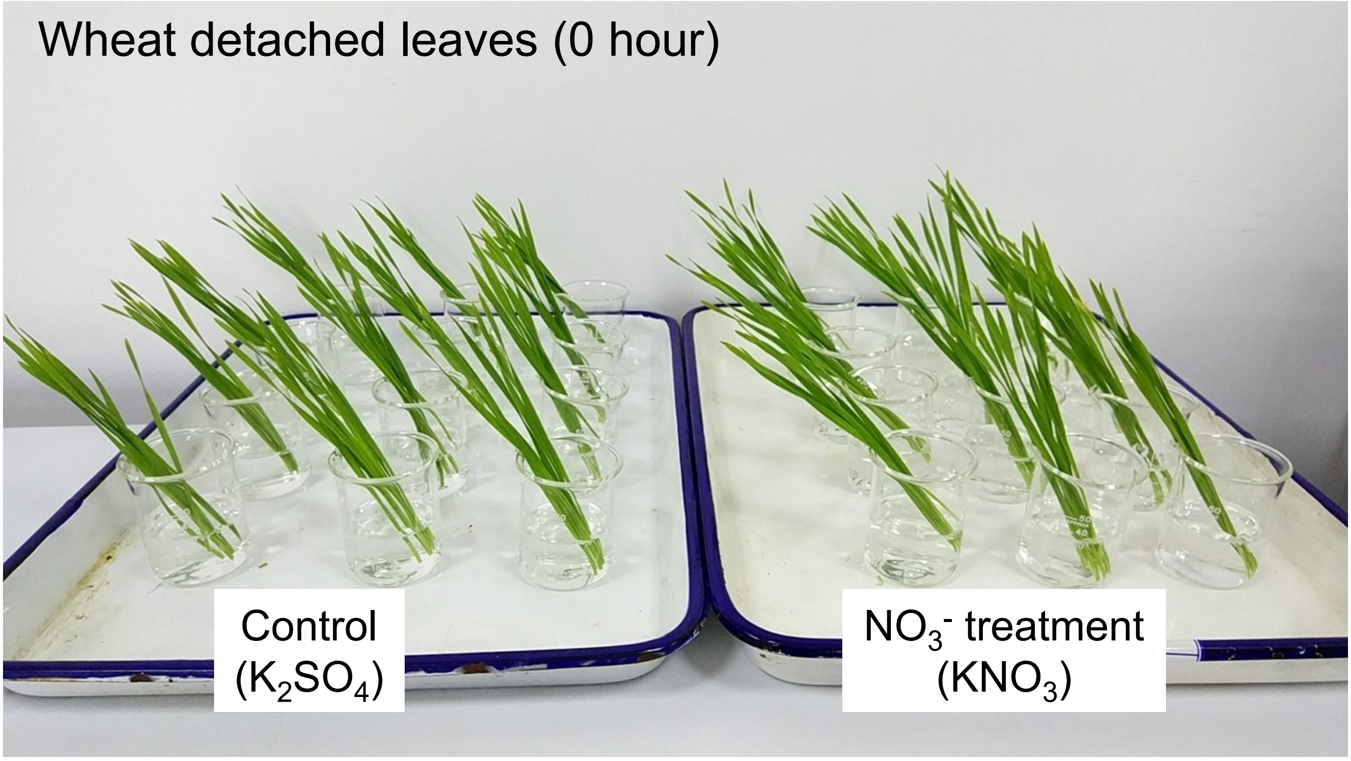

Supplementary Figure S1. Photographs of wheat seedlings used in the nitrate induction experiment. (A) Two-week old wheat seedlings grown in a laboratory. (B) Detached wheat leaves under incubation with K_2SO_4 solution and KNO_3 solution at 0 hour.

A

```
Tappc1bA    MSSSSAAPMERHHSIDAQLRLLAPGKVSEDDKLVEYDALLVDRFLDILQDLHGPHLREFVQDCYEVAAEYEGDRDAARLD   80
Tappc1bB    MSSSSAAPMERHHSIDAQLRLLAPGKVSEDDKLVEYDALLVDRFLDILQDLHGPHLREFVQDCYEVAAEYEGDRDAARLD   80
Tappc1bD    MSSSSAAPMERHHSIDAQLRLLAPGKVSEDDKLVEYDALLVDRFLDILQDLHGPHLREFVQDCYEVAAEYEGDRDAARLD   80

Tappc1bA    ELGGRLTGLAPADSIVVASSFSHMLNLANLAEEVQIANRRRNKLKRGDFADEASATTESDIEETLKRLVSDLGKTREEVF  160
Tappc1bB    ELGGRLTGLAPADSIVVASSFSHMLNLANLAEEVQIANRRRNKLKRGDFADEASATTESDIEETLKRLVSDLGKTREEVF  160
Tappc1bD    ELGGRLTGLAPADSIVVASSFSHMLNLANLAEEVQIANRRRNKLKRGDFADEASATTESDIEETLKRLVSDLGKTREEVF  160

Tappc1bA    DALKNQTVDLVFTAHPTQSIRRSLLQKHGRIRNCLRQLYAKDITADDKQELDEALQREIQAAFRTD                226
Tappc1bB    DALKNQTVDLVFTAHPTQSIRRSLLQKHGRIRNCLRQLYAKDITADDKQELDEALQREIQAAFRTD                226
Tappc1bD    DALKNQTVDLVFTAHPTQSIRRSLLQKHGRIRNCLRQLYAKDITADDKQELDEALQREIQAAFRTD                226
                      -------
```

B

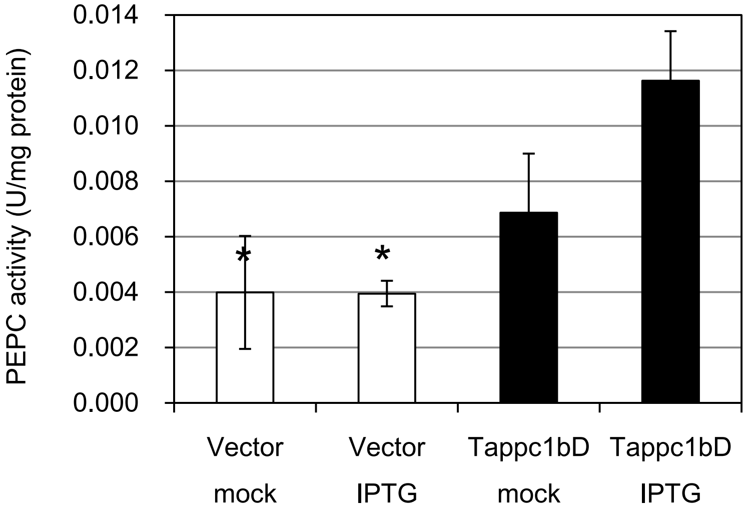

Supplementary Figure S2. Prokaryotic expression of a Tappc1b fragment in *E. coli*. (A) A partial sequence alignment of Tappc1b in the wheat genome (100% identical among each other). The expressed polypeptide fragment was shown. One of the conserved domain, which is important for PEPC enzyme catalysis, was underlined. (B) PEPC activity in *E. coli* BL21 (DE3) harboring pET28a and pET28a with the Tappc1bD fragment insert. Overnight culture of transformed *E. coli* were inoculated onto fresh LB medium, and IPTG was added when OD600 reached onto 0.5. Then, the bacterial cultures were incubated at 25° C for five hours. *E. coli* cells were harvested to extract soluble proteins, and PEPC activities were measured. Asterisks indicate statistical significance between "Tappc1bD IPTG" and other samples at 5% level.

Supplementary Figure 3. A multiple sequence alignment of wheat PEPC proteins.

```
                                  plastid transit peptide+++++++++++++++++++++++++++
Tappc4B_TraesCS3B02G168000_       -----------------------MISLHTSPFTLLIKLEPGRRRLALP-Q 26
Tappc4D_TraesCS3D02G150500_       -----------------------MISLHTSPFTLLIKLEPGRRRLALP-Q 26
Tappc4A_TraesCS3A02G134200_       -----------------------MISLHTSPFTLLIKLEPGRRRLALPPQ 27
Tappc1aA_TraesCS6A02G195600_      --------------------------------------------------
Tappc1aD_TraesCS6D02G183200_      --------------------------------------------------
Tappc1aB_TraesCS6B02G223100_      --------------------------------------------------
Tappc1bA_TraesCS7A02G345400_      --------------------------------------------------
Tappc1bD_TraesCS7D02G333900_      --------------------------------------------------
Tappc2B_TraesCS5B02G179800_       --------------------------------------------------
Tappc2D_TraesCS5D02G186200_       --------------------------------------------------
Tappc2A_TraesCS5A02G181800_       --------------------------------------------------
Tappc3A_TraesCS3A02G306700_       --------------------------------------------------
Tappc3D_TraesCS3D02G295200_       --------------------------------------------------
Tappc3B_TraesCS3B02G329800_       --------------------------------------------------
Tappc-bB_TraesCS3B02G008500_      --------------------------------------------------
Tappc-bD_TraesCS3D02G005000_      --------------------------------------------------

                                  +++++++----++++++++ plastid transit peptide
                                                          *(phosphorylation)
Tappc4B_TraesCS3B02G168000_       YAVLRRP----APRS-VRAAAVAES-------VDAQLRQFARG-GATEDD 63
Tappc4D_TraesCS3D02G150500_       YAVLRRP----APRSGVRAAAVAES-------VDAQLRQFARG-GATEDD 64
Tappc4A_TraesCS3A02G134200_       YAALRRP----APRS-VRAAAVAES-------VDAQLRQFARG-GATEDD 64
Tappc1aA_TraesCS6A02G195600_      ----------MAAPSGKAAMERHQS-------IDAQLRLLVPG--KVSED 31
Tappc1aD_TraesCS6D02G183200_      ----------MAAPSGKAAMERHQS-------IDAQLRLLVPG--KVSED 31
Tappc1aB_TraesCS6B02G223100_      ----------MAAPSGKAAMERHQS-------IDAQLRLLVPG--KVSED 31
Tappc1bA_TraesCS7A02G345400_      -----------MSSSSAAPMERHHS-------IDAQLRLLAPG--KVSED 30
Tappc1bD_TraesCS7D02G333900_      -----------MSSSSAAPMERHHS-------IDAQLRLLAPG--KVSED 30
Tappc2B_TraesCS5B02G179800_       -------MASSAPGGGSGKIERLSS-------IDAQLRLLVPA--KVSED 34
Tappc2D_TraesCS5D02G186200_       -------MASSAPGGGSGKIERLSS-------IDAQLRLLVPA--KVSED 34
Tappc2A_TraesCS5A02G181800_       -------MALSAPGGGSGKIERLSS-------IDAQLRLLVPA--KVSED 34
Tappc3A_TraesCS3A02G306700_       ----------MARN----AADKATS-------IDAQLRMLAPK--KLSDD 27
Tappc3D_TraesCS3D02G295200_       ----------MARN----AADKATS-------IDAQLRMLAPK--KLSED 27
Tappc3B_TraesCS3B02G329800_       ----------MARN----AADKATS-------IDAQLRMLAPK--KLSED 27
Tappc-bB_TraesCS3B02G008500_      --------------------------------MPDTTDDVAEGISFQAFE 18
Tappc-bD_TraesCS3D02G005000_      --------------------------------MPDTTDDVAEGISFQAFE 18
```

```
Tappc4B_TraesCS3B02G168000_       DRLQNYETLLVTRFLDIIQD-LHGSNFRRVVEECLRVSAEYQRDLGDRAA 112
Tappc4D_TraesCS3D02G150500_       DRLQNYEALLVTRFLDIIQD-LHGSNFRRVVEECLRVSAEYQRDAGDRAA 113
Tappc4A_TraesCS3A02G134200_       DRLQNYEALLVTRFLDIIQD-LHGSNFRRVVEECLRVSGEYQRDVGDRAA 113
Tappc1aA_TraesCS6A02G195600_      DKLVEYDALLVDRFLDILQD-LHGPHLREFVQECYELSAEYETDRDE--A 78
Tappc1aD_TraesCS6D02G183200_      DKLVEYDALLVDRFLDILQD-LHGPHLREFVQECYELSAEYETDRDE--A 78
Tappc1aB_TraesCS6B02G223100_      DKLVEYDALLVDRFLDILQD-LHGPHLREFVQECYELSAEYETDRDE--A 78
Tappc1bA_TraesCS7A02G345400_      DKLVEYDALLVDRFLDILQD-LHGPHLREFVQDCYEVAAEYEGDRDA--A 77
Tappc1bD_TraesCS7D02G333900_      DKLVEYDALLVDRFLDILQD-LHGPHLREFVQDCYEVAAEYEGDRDA--A 77
Tappc2B_TraesCS5B02G179800_       DKLIEYDALLLDRFLDVLQG-LHGDDLREMVQECYEVAAEYETKHDL--E 81
Tappc2D_TraesCS5D02G186200_       DKLIEYDALLLDRFLDVLQG-LHGDDLREMVQECYEVAAEYETKHDL--E 81
Tappc2A_TraesCS5A02G181800_       DKLIEYDALLLDRFLDVLQG-LHGDDLREMVQECYEVAAEYETKHDL--E 81
Tappc3A_TraesCS3A02G306700_       DKLVEYDALLIERFLCILQG-LQGDKIRKTVQECYELAAEYERTLDP--K 74
Tappc3D_TraesCS3D02G295200_       DELVEYDALLIERFLCILQG-LQGDKIRETVQECYELAAEYERTLDP--K 74
Tappc3B_TraesCS3B02G329800_       DKLVEYDALLIERFLCILQG-LQGDKIRETVQECYELAAEYERTLDP--K 74
Tappc-bB_TraesCS3B02G008500_      DDCRLLGSLLHEVLLRELGPGFVKLFERIRILAQSAMTMRGAEMEDTATV 68
Tappc-bD_TraesCS3D02G005000_      DDCRLLGSLLHEVLLRELGPGFVKLFERIRILAQSAMTMRGAGMEDTAAV 68

Tappc4B_TraesCS3B02G168000_       KLGELGALFTSLDVGDAIMVSSSLSHMLNLANLAEEIQMVYQKKMETSRR 162
Tappc4D_TraesCS3D02G150500_       KLGELGALFTSLDVGDAIMVSSSLSHMLNLANLAEEIQMVYQKKMETSRR 163
Tappc4A_TraesCS3A02G134200_       KLGELGALFTSLDVGDAIMVSSSLSHMLNLANLAEEIQMVYQKKMETSRR 163
Tappc1aA_TraesCS6A02G195600_      RIAELGSKLTSLSPADSIVVSSSFSHMLNLANLAEEVQIAFR-RRSKLKR 127
Tappc1aD_TraesCS6D02G183200_      RIAELGSKLTSLSPADSIVVSSSFSHMLNLANLAEEVQIAFR-RRSKLKR 127
Tappc1aB_TraesCS6B02G223100_      RIGELGGKLTSLSPADSIVVSSSFSHMLNLANLAEEVQIAFR-RRSKLKR 127
Tappc1bA_TraesCS7A02G345400_      RLDELGGRLTGLAPADSIVVASSFSHMLNLANLAEEVQIANRRRNKL-KR 126
Tappc1bD_TraesCS7D02G333900_      RLDELGGRLTGLAPADSIVVASSFSHMLNLANLAEEVQIANRRRNKL-KR 126
Tappc2B_TraesCS5B02G179800_       KLDELGEMITSLDPGDSIVIAKAFSHMLNLANLAEEVQIAYR-RRVKLKK 130
Tappc2D_TraesCS5D02G186200_       KLDELGEMITSLDPGDSIVIAKAFSHMLNLANLAEEVQIAYR-RRVKLKK 130
Tappc2A_TraesCS5A02G181800_       KLDELGEMITSLDPGDSIVIAKAFSHMLNLANLAEEVQIAYR-RRVKLKK 130
Tappc3A_TraesCS3A02G306700_       QLDEIGNLLARLDPEDSIVTTKSLSHMLILANLAEEVQIAYR-RRQKLKS 123
Tappc3D_TraesCS3D02G295200_       QLDEIGNLLARLDPEDSIVTTKSLSHMLILANLAEEVQIAYR-RRQKLKS 123
Tappc3B_TraesCS3B02G329800_       QLDEIGNLLARLDPEDSIVTTKSLSHMLILANLAEEVQIAYR-RRQKLKS 123
Tappc-bB_TraesCS3B02G008500_      VERQLEAELSAMSLEDSLSLARAFAHHLNLMGIAERHHRVRK-------- 110
Tappc-bD_TraesCS3D02G005000_      VERQLEAELSAMSLEDSLSLARAFAHHLNLMGIAERHHRVRK-------- 110

                                                             Catalytic base*
                                                                      ++++++ subdomain I
Tappc4B_TraesCS3B02G168000_       GGFADEALAPTESDIDETFQRIVGGLGKTPQEVFDALRSQTIDLVFTAHP 212
Tappc4D_TraesCS3D02G150500_       GGFADEALAPTESDIDETFQRMVGGLGKTPQEVFDALRSQTIDLVFTAHP 213
```

```
Tappc4A_TraesCS3A02G134200_       GGFADEALAPTESDIDETFQRMVGGLGKTPQEVFDALRAQTTDLVFTAHP 213
Tappc1aA_TraesCS6A02G195600_      GDFGDEASAPTESDIEETLKRLVSELGKSREEVFDALKNQTVDLVFTAHP 177
Tappc1aD_TraesCS6D02G183200_      GDFGDEASAPTESDIEETLKRLVSELGKSREEVFDALKNQTVDLVFTAHP 177
Tappc1aB_TraesCS6B02G223100_      GDLGDEASAPTESDIEETLKRLVSELGKSREEVFDSLKNQTVDLVFTAHP 177
Tappc1bA_TraesCS7A02G345400_      GDFADEASATTESDIEETLKRLVSDLGKTREEVFDALKNQTVDLVFTAHP 176
Tappc1bD_TraesCS7D02G333900_      GDFADEASATTESDIEETLKRLVSDLGKTREEVFDALKNQTVDLVFTAHP 176
Tappc2B_TraesCS5B02G179800_       GDFADENSAITESDIEETLKRLVFDMKKSPAEVFDALKNQTVDLVLTAHP 180
Tappc2D_TraesCS5D02G186200_       GDFADENSAITESDIEETLKRLVFDMKKSPAEVFDALKNQTVDLVLTAHP 180
Tappc2A_TraesCS5A02G181800_       GDFADENSAITESDIEETLKRLVFDMKKSPAEVFDALKNQTVDLVLTAHP 180
Tappc3A_TraesCS3A02G306700_       GDFADENSATTESDIEETLKRLVCQLNKSPLEVFDALKNQTVDLVLTAHP 173
Tappc3D_TraesCS3D02G295200_       GDFADENSATTESDIEETLKRLVCQLNKSPLEVFDALKNQTVDLVLTAHP 173
Tappc3B_TraesCS3B02G329800_       GDFADENSATTESDIEETLKRLVCQLKKSPLEVFDALKNQTVDLVLTAHP 173
Tappc-bB_TraesCS3B02G008500_      ----SRSEVHLSKSCHDIFDKLIK-GGVPPEQLYDTVCKQGVEIVLTAHP 155
Tappc-bD_TraesCS3D02G005000_      ----SRSEVHLSKSCHDIFDKLIQ-GGVPPEQLYDTVCKQGVEIVLTAHP 155

                                      **Catalytic base
Tappc4B_TraesCS3B02G168000_       TQSIRRSLLEKHASIRTCLTQLCVEGVSDNEKQEIDEALQREILAAFRTD 262
Tappc4D_TraesCS3D02G150500_       TQSIRRSLLEKHASIRTCLTQLCVEGVSENEKQEIDEALQREILAAFRTD 263
Tappc4A_TraesCS3A02G134200_       TQSIRRSLLEKHASIRTCLTQLCVEGVSENEKQEIDEALQREILAAFRTD 263
Tappc1aA_TraesCS6A02G195600_      TQSVRRSLLQKHGRIRNCLRQLYAKDITADDKQELDEALQREIQAAFRTD 227
Tappc1aD_TraesCS6D02G183200_      TQSVRRSLLQKHGRIRNCLRQLYAKDITADDKQELDEALQREIQAAFRTD 227
Tappc1aB_TraesCS6B02G223100_      TQSVRRSLLQKHGRIRNCLRQLYAKDITADDKQELDEALQREIQAAFRTD 227
Tappc1bA_TraesCS7A02G345400_      TQSIRRSLLQKHGRIRNCLRQLYAKDITADDKQELDEALQREIQAAFRTD 226
Tappc1bD_TraesCS7D02G333900_      TQSIRRSLLQKHGRIRNCLRQLYAKDITADDKQELDEALQREIQAAFRTD 226
Tappc2B_TraesCS5B02G179800_       TQSVRRSLLQKHSRIRNCLVQLYSKDITPDDKQELDEALQREIQAAFRTD 230
Tappc2D_TraesCS5D02G186200_       TQSVRRSLLQKHSRIRNCLVQLYSKDITPDDKQELDEALQREIQAAFRTD 230
Tappc2A_TraesCS5A02G181800_       TQSVRRSLLQKHSRIRNCLVQLYSKDITPDDKQELDEALQREIQAAFRTD 230
Tappc3A_TraesCS3A02G306700_       TQSVRRSLLQKHGRIRSCLTQLYAKDITPDEKQELDEALQREIQAAFRTD 223
Tappc3D_TraesCS3D02G295200_       TQSVRRSLLQKHGRIRNCLTQLYAKDITPDEKQELDEALQREIQAAFRTD 223
Tappc3B_TraesCS3B02G329800_       TQSVRRSLLQKHGRIRNCLTQLYAKDITPDEKQELDEALQREIQAAFRTD 223
Tappc-bB_TraesCS3B02G008500_      TQINRRTLQYKHLRIAHLLEFNGRRDLNYEDREMLIEDLVREITALWQTD 205
Tappc-bD_TraesCS3D02G005000_      TQINRRTLQYKHLRIAHLLEFNGRRDLNYEDREMLIEDLVREITALWQTD 205

                                     *G6P binding site
Tappc4B_TraesCS3B02G168000_       EIRRTPPTPQDEMRAGMSYFHDTIWNGVPKFLRRVDTALKNIGIDERLPY 312
Tappc4D_TraesCS3D02G150500_       EIRRTPPTPQDEMRAGMSYFHDTIWNGVPKFLRRVDTALKNIGIDERLPY 313
Tappc4A_TraesCS3A02G134200_       EIRRTPPTPQDEMRAGMSYFHDTIWNGVPKFLRRVDTALRNIGIEERLPY 313
Tappc1aA_TraesCS6A02G195600_      EIRRTPPTPQDEMRAGMSYFHETIWKGVPKFLRRIDTALKNIGINERLPY 277
```

```
Tappc1aD_TraesCS6D02G183200_      EIRRTPPTPQDEMRAGMSYFHETIWKGVPKFLRRIDTALKNIGINERLPY 277
Tappc1aB_TraesCS6B02G223100_      EIRRTPPTPQDEMRAGMSYFHETIWKGVPKFLRRIDTALKNIGINERLPY 277
Tappc1bA_TraesCS7A02G345400_      EIRRTQPTPQDEMRAGMSYFHETIWKGVPKFLRRVDTALKNIGIDERLPY 276
Tappc1bD_TraesCS7D02G333900_      EIRRTQPTPQDEMRAGMSYFHETIWKGVPKFLRRVDTALKNIGIDERLPY 276
Tappc2B_TraesCS5B02G179800_       EIRRTQPTPQDEMRAGMSYFHETIWKGVPKFLRRVDTALKNIGINERVPY 280
Tappc2D_TraesCS5D02G186200_       EIRRTQPTPQDEMRAGMSYFHETIWKGVPKFLRRVDTALKNIGINERVPY 280
Tappc2A_TraesCS5A02G181800_       EIRRTQPTPQDEMRAGMSYFHETIWKGVPKFLRRVDTALKNIGINERVPY 280
Tappc3D_TraesCS3D02G295200_       EIRRTPPTPQDEMRAGMSYFHETIWKGVPKFLRRVDTALKNIGIKERVPY 273
Tappc3B_TraesCS3B02G329800_       EIRRAPPTPQDEMRAGMSYFHETIWKGVPKFLRRVDTALKNIGIKERVPY 273
Tappc-bB_TraesCS3B02G008500_      ELRRHKPTPVDEARAGLHIVEQSLWKSIPRYLRRVSNALKKH-TGKPLPL 254
Tappc-bD_TraesCS3D02G005000_      ELRRHKPTPVDEARAGLHIVEQSLWKSIPRYLRRVSNALKKH-TGKPLPL 254

                                          *PEP binding site
Tappc4B_TraesCS3B02G168000_       DVPLIQFSSWMGGDRDGNPRVTPEVTRDVCLLARMMAANMYFSKMGSLMF 362
Tappc4D_TraesCS3D02G150500_       DVPLIQFSSWMGGDRDGNPRVTPEVTRDVCLLARMMAANMYFSKMGSLMF 363
Tappc4A_TraesCS3A02G134200_       DVPLIQFSSWMGGDRDGNPRVTPEVTRDVCLLARMMAANMYFSKMGSLMF 363
Tappc1aA_TraesCS6A02G195600_      NAPLIQFSSWMGGDRDGNPRVTPEVTRDVCLLARMMAANLYFSQIEDLMF 327
Tappc1aD_TraesCS6D02G183200_      NAPLIQFSSWMGGDRDGNPRVTPEVTRDVCLLARMMAANLYFSQIEDLMF 327
Tappc1aB_TraesCS6B02G223100_      NAPLIQFSSWMGGDRDGNPRVTPEVTRDVCLLARMMAANLYFSQIEDLMF 327
Tappc1bA_TraesCS7A02G345400_      NAPLIQFSSWMGGDRDGNPRVTPEVTRDVCLLARMMAANLHFSQIEELMF 326
Tappc1bD_TraesCS7D02G333900_      NAPLIQFSSWMGGDRDGNPRVTPEVTRDVCLLARMMAANLHFSQIEELMF 326
Tappc2B_TraesCS5B02G179800_       NAPLIQFSSWMGGDRDGNPRVTPEVTRDVCLLARMMAANLYCAQIEDLMF 330
Tappc2D_TraesCS5D02G186200_       NAPLIQFSSWMGGDRDGNPRVTPEVTRDVCLLARMMAANLYCAQIEDLMF 330
Tappc2A_TraesCS5A02G181800_       NAPLIQFSSWMGGDRDGNPRVTPEVTRDVCLLARMMAANLYCAQIEDLMF 330
Tappc3A_TraesCS3A02G306700_       NAPLIQFSSWMGGDRDGNPRVTPEVTRDVCLLARMMAANLYYAQIEDLMF 323
Tappc3D_TraesCS3D02G295200_       NAPLIQFSSWMGGDRDGNPRVTPEVTRDVCLLARMMAANLYYAQIEDLMF 323
Tappc3B_TraesCS3B02G329800_       NAPLIQFSSWMGGDRDGNPRVTPEVTRDVCLLARMMAANLYYAQIEDLMF 323
Tappc-bB_TraesCS3B02G008500_      TCTPIKFGSWMGGDRDGNPNVTSKVTRDVSLMARWMAIDLYIRQLDSLSF 304
Tappc-bD_TraesCS3D02G005000_      TCTPIKFGSWMGGDRDGNPNVTSKVTRDVSLMARWMAIDLYIRQLDSLSF 304

Tappc4B_TraesCS3B02G168000_       ELSMWRCNDELRARADELHRL----------------------------- 383
Tappc4D_TraesCS3D02G150500_       ELSMWRCNDELRARADELHRL----------------------------- 384
Tappc4A_TraesCS3A02G134200_       ELSMWRCNDELRARADELHRL----------------------------- 384
Tappc1aA_TraesCS6A02G195600_      ELSMWRCSDELRVRADELHC------------------------------ 347
Tappc1aD_TraesCS6D02G183200_      ELSMWRCSDELRVRADELHC------------------------------ 347
Tappc1aB_TraesCS6B02G223100_      ELSMWRCSDELRVRADELHR------------------------------ 347
Tappc1bA_TraesCS7A02G345400_      ELSMWRCNDELRVRAEELHR------------------------------ 346
Tappc1bD_TraesCS7D02G333900_      ELSMWRCNDELRVRAEELHR------------------------------ 346
```

```
Tappc2B_TraesCS5B02G179800_      ELSMWRCNDELRSRADELHQS----------------------------- 351
Tappc2D_TraesCS5D02G186200_      ELSMWRCSDELRSRADELHRS----------------------------- 351
Tappc2A_TraesCS5A02G181800_      ELSMWRCNDELRSRADELHRS----------------------------- 351
Tappc3A_TraesCS3A02G306700_      ELSMWRCSDELRVKADQLHRA----------------------------- 344
Tappc3D_TraesCS3D02G295200_      ELSMWRCSDELRIKADQLHRA----------------------------- 344
Tappc3B_TraesCS3B02G329800_      ELSMWRCSDELRVKADQLHRA----------------------------- 344
Tappc-bB_TraesCS3B02G008500_     ELSIKKCSDKLATLANEILLK--ESTSEEKKANPWTKTGPENNLKRPPRM 352
Tappc-bD_TraesCS3D02G005000_     ELSIKKCSDKLATLANEILLKDSESTSEEKKANPWTKTGPENNLKRPPRM 354

Tappc4B_TraesCS3B02G168000_      --------------------------------------------------
Tappc4D_TraesCS3D02G150500_      --------------------------------------------------
Tappc4A_TraesCS3A02G134200_      --------------------------------------------------
Tappc1aA_TraesCS6A02G195600_     --------------------------------------------------
Tappc1aD_TraesCS6D02G183200_     --------------------------------------------------
Tappc1aB_TraesCS6B02G223100_     --------------------------------------------------
Tappc1bA_TraesCS7A02G345400_     --------------------------------------------------
Tappc1bD_TraesCS7D02G333900_     --------------------------------------------------
Tappc2B_TraesCS5B02G179800_      --------------------------------------------------
Tappc2D_TraesCS5D02G186200_      --------------------------------------------------
Tappc2A_TraesCS5A02G181800_      --------------------------------------------------
Tappc3A_TraesCS3A02G306700_      --------------------------------------------------
Tappc3D_TraesCS3D02G295200_      --------------------------------------------------
Tappc3B_TraesCS3B02G329800_      --------------------------------------------------
Tappc-bB_TraesCS3B02G008500_     GMPAQLPSGADLPSLTECSDRESQFRIVKLPCNPKHQGIQIPTERSEESH 402
Tappc-bD_TraesCS3D02G005000_     GMPAQLPSGADLPSLTECSDSESQFRIVKLPCNPKHQGIQIPTERSEESH 404

Tappc4B_TraesCS3B02G168000_      ------------------------------SSRKYAKYYIEFWKQISPRE 403
Tappc4D_TraesCS3D02G150500_      ------------------------------SSRKYAKYYIEFWKQISPRE 404
Tappc4A_TraesCS3A02G134200_      ------------------------------SSRKYAKYYIEFWKQISPRE 404
Tappc1aA_TraesCS6A02G195600_     ------------------------------SSKKSAKHYIEFWKQVPSNE 367
Tappc1aD_TraesCS6D02G183200_     ------------------------------SSKKSAKHYIEFWKQVPSNE 367
Tappc1aB_TraesCS6B02G223100_     ------------------------------SSKKSAKHYIEFWKQVPSNE 367
Tappc1bA_TraesCS7A02G345400_     ------------------------------ASKKAEKHYIEFWKQVPPTE 366
Tappc1bD_TraesCS7D02G333900_     ------------------------------ASRKAEKHYIEFWKQVPPTE 366
Tappc2B_TraesCS5B02G179800_      ------------------------------SKKD-AKHYIEFWKKVPPNE 370
Tappc2D_TraesCS5D02G186200_      ------------------------------SKKD-AKHYIEFWKKVPPNE 370
Tappc2A_TraesCS5A02G181800_      ------------------------------SKKD-AKHYIEFWKKVPPNE 370
Tappc3A_TraesCS3A02G306700_      ------------------------------SKKDTTKHYIEFWKQVPPSE 364
```

```
Tappc3D_TraesCS3D02G295200_     ------------------------------SKKDTTKHYIEFWKQVPPSE 364
Tappc3B_TraesCS3B02G329800_     ------------------------------SKKDTTKHYIEFWKQVPPSE 364
Tappc-bB_TraesCS3B02G008500_    SPVQSPGRRPAS--SHMSRTPSGSQLRKMLFTESKIGRSS-FRKLLEPSL 449
Tappc-bD_TraesCS3D02G005000_    SPVQSPGRRPSS--SHMSRTPSGSQLRKMLFTESKIGRSS-FRKLLEPSL 451

                                         *PEP binding site(G6P binding?)
Tappc4B_TraesCS3B02G168000_     -------PYRIILGDVRDKLYNTCERAREILSNGESSIPEEDTYTRVE-- 444
Tappc4D_TraesCS3D02G150500_     -------PYRIILGDVRDKLYNTCERAREILSNGESSIPEEDTYTRVE-- 445
Tappc4A_TraesCS3A02G134200_     -------PYRIILGDVRDKLYNTCERAREILSSGESSIPEEDTYTRVE-- 445
Tappc1aA_TraesCS6A02G195600_    -------PYRVILGDVRDKLYYTRERSRHILTTGVSDIPEESTFTNVE-- 408
Tappc1aD_TraesCS6D02G183200_    -------PYRVILGDVRDKLYYTRERSRHILTTGVSDIPEESTFTNVE-- 408
Tappc1aB_TraesCS6B02G223100_    -------PYRVILGDVRDKLYYTRERSRHILTTGVSDIPEESTFTNVE-- 408
Tappc1bA_TraesCS7A02G345400_    -------PYRVILGYVRDKLYYTRERSRHLLTSGTSDIPADSTFTDVE-- 407
Tappc1bD_TraesCS7D02G333900_    -------PYRVILGYVRDKLYYTRERSRHLLTSGSSDIPADSTFTDVE-- 407
Tappc2B_TraesCS5B02G179800_     -------PYRVILGDVRDNLYNTRERSRELLSSGHSDIPEEATLTNLE-- 411
Tappc2D_TraesCS5D02G186200_     -------PYRVILGDVRDNLYNTRERSRELLSSGHSDIPEEATLTNLE-- 411
Tappc2A_TraesCS5A02G181800_     -------PYRVILGDVRDNLYNTRERSRELLSSGHSDIPEEATLTNLE-- 411
Tappc3A_TraesCS3A02G306700_     -------PYRVILSDVRDKLYNTRERSRHLLASGFSEIPDDAIFTDVE-- 405
Tappc3D_TraesCS3D02G295200_     -------PYRVILSDVRDKLYNTRERSRHLLASGFSEIPDDAIFTDVE-- 405
Tappc3B_TraesCS3B02G329800_     -------PYRVILSDVRDKLYNTRERSRHLLASGFSEIPDDAIFTDVE-- 405
Tappc-bB_TraesCS3B02G008500_    SDKPGITPYRIVLGNVKEKMMKTRRRLEHLLEDLPCDSDPAEYYETPD-- 497
Tappc-bD_TraesCS3D02G005000_    SDKPGITPYRIVLGNVKEKMMKTRRRLEHLLEDLPCDSDPAEYYETPD-- 499

Tappc4B_TraesCS3B02G168000_     -----------------------------EFLEPLELCYRSLCDCGDKLI 465
Tappc4D_TraesCS3D02G150500_     -----------------------------EFLEPLELCYRSLCDCGDKLI 466
Tappc4A_TraesCS3A02G134200_     -----------------------------EFLEPLELCYRSLCDCGDKLI 466
Tappc1aA_TraesCS6A02G195600_    -----------------------------MFLEPLELCYRSLCACGDKPI 429
Tappc1aD_TraesCS6D02G183200_    -----------------------------MFLEPLELCYRSLCACGDKPI 429
Tappc1aB_TraesCS6B02G223100_    -----------------------------MFLEPLELCYRSLCACGDKPI 429
Tappc1bA_TraesCS7A02G345400_    -----------------------------QFLEPLELCYRSLCDCGDKTV 428
Tappc1bD_TraesCS7D02G333900_    -----------------------------QFLEPLELCYRSLCDCGDKTV 428
Tappc2B_TraesCS5B02G179800_     -----------------------------QLLEPLELCYRSLCACGDRVI 432
Tappc2D_TraesCS5D02G186200_     -----------------------------QLLEPLELCYRSLCACGDRVI 432
Tappc2A_TraesCS5A02G181800_     -----------------------------QLLEPLELCYRSLCACGDRVI 432
Tappc3A_TraesCS3A02G306700_     -----------------------------QFLEPLELCYRSLCACGDHTI 426
Tappc3D_TraesCS3D02G295200_     -----------------------------QFLEPLELCYRSLCACGDHTI 426
Tappc3B_TraesCS3B02G329800_     -----------------------------QFLEPLELCYRSLCACGDHTI 426
Tappc-bB_TraesCS3B02G008500_    -----------------------------QLLEPLLLCYDSLQSCGSSIL 518
```

Tappc-bD_TraesCS3D02G005000_ ----------------------------QLLEPLLLCYDSLQSCGSSIL 520

*PEP binding site

Tappc4B_TraesCS3B02G168000_ ADGSLLDFMRQVSTFGLCLLKLDIRQESDRHIDAVDAITTHLGIGSYRDW 515
Tappc4D_TraesCS3D02G150500_ ADGSLLDFMRQVSTFGLCLLKLDIRQESDRHIDAVDAITTHLGIGSYRDW 516
Tappc4A_TraesCS3A02G134200_ ADGSLLDFMRQVSTFGLCLLKLDIRQESDRHIDAVDAITTHLGIGSYRDW 516
Tappc1aA_TraesCS6A02G195600_ ADGSLLDFLRQVSTFGLALVKLDIRQESDRHTDVLDTITTHLGIGSYAEW 479
Tappc1aD_TraesCS6D02G183200_ ADGSLLDFLRQVSTFGLALVKLDIRQESDRHTDVLDTITTHLGIGSYAEW 479
Tappc1aB_TraesCS6B02G223100_ ADGSLLDFLRQVSTFGLALVKLDIRQESDRHTDVLDTITTHLGIGSYAEW 479
Tappc1bA_TraesCS7A02G345400_ ADGSLLDFLRQVSTFGLSLVKLDIRQESDRHTDALDAITAHLGIGSYRSW 478
Tappc1bD_TraesCS7D02G333900_ ADGSLLDFLRQVSTFGLSLVKLDIRQESDRHTDALDAITAHLGIGSYRSW 478
Tappc2B_TraesCS5B02G179800_ ADGTLLDFLRQVSTFGLSLVKLDIRQESDRHTDALDAITSYLGIGSYREW 482
Tappc2D_TraesCS5D02G186200_ ADGTLLDFLRQVSTFGLSLVKLDIRQESDRHTDALDAITSYLGIGSYREW 482
Tappc2A_TraesCS5A02G181800_ ADGTLLDFLRQVSTFGLSLVKLDIRQESDRHTDALDAITSYLGIGSYREW 482
Tappc3A_TraesCS3A02G306700_ ADGNLLDFLRQVSTFGLSLVRLDIRQESERHTDVMDVITKYLGVGSYREW 476
Tappc3D_TraesCS3D02G295200_ ADGNLLDFLRQVSTFGLSLVRLDIRQESERHTDVMDAITKYLGVGSYREW 476
Tappc3B_TraesCS3B02G329800_ ADGNLLDFLRQVSTFGLSLVRLDIRQESERHTDVMDAITKYLGVGSYRKW 476
Tappc-bB_TraesCS3B02G008500_ ADGRLADLIRRVATFGMVLMKLDVRQESGRHTEALDAITSYLDLGVYSEW 568
Tappc-bD_TraesCS3D02G005000_ ADGRLADLIRRVATFGMVLMKLDVRQESGRHTEALDAITSYLDLGVYSEW 570

*Tetramer formation

*Tetramer formation

Tappc4B_TraesCS3B02G168000_ PEEQRQEWLVNELRGNRPLFG--PDLP-QSDEVADVLGTFRVISELPADS 562
Tappc4D_TraesCS3D02G150500_ PEEQRQEWLVNELRGNRPLFG--PDLP-QSDEVADVLGTFRVISELPADS 563
Tappc4A_TraesCS3A02G134200_ PEEQRQDWLVNELRGNRPLFG--PDLP-QSDEVADVLGTFRVIAELPADS 563
Tappc1aA_TraesCS6A02G195600_ SEEKRQEWLLSELRGKRPLFG--SDLP-QTEEVADVLSTFHILAELPADC 526
Tappc1aD_TraesCS6D02G183200_ SEEKRQEWLLSELRGKRPLFG--SDLP-QTEEVADVLGTFHILAELPADC 526
Tappc1aB_TraesCS6B02G223100_ SEEKRQEWLLSELRGKRPLFG--SDLP-QTEEVADVLGTFHILAELPADC 526
Tappc1bA_TraesCS7A02G345400_ PEEKRQEWLLSELRGRRPLFG--GDLP-MTEEVADVLGTFRVLAELPPDC 525
Tappc1bD_TraesCS7D02G333900_ PEEKRQEWLLSELRGRRPLFG--GDLP-MTEEVADVLGTFRVLAELPPDC 525
Tappc2B_TraesCS5B02G179800_ SEERRQEWLLSELNGKRPLFG--ADLP-MTEEVADVMGAFQVIAELPGDN 529
Tappc2D_TraesCS5D02G186200_ SEERRQEWLLSELNGKRPLFG--ADLP-MTEEVADVMGAFQVIAELPGDN 529
Tappc2A_TraesCS5A02G181800_ SEEHRQEWLLSELNGKRPLFG--ADLP-MTEEVADVMGAFQVIAELPGDN 529
Tappc3A_TraesCS3A02G306700_ SEEKRQEWLLFELNGKRPLFG--PDLP-KTNEVAEVLDTFHVLAELPSDS 523
Tappc3D_TraesCS3D02G295200_ SEEKRQEWLLFELNGKRPLFG--PDLP-KTNEVAEVVDTFHVLAELPSDS 523
Tappc3B_TraesCS3B02G329800_ SEEKRQEWLLFELNGKRPLFG--PDLP-KTNEVAEVLDTFHVLAELPSDS 523
Tappc-bB_TraesCS3B02G008500_ DEEKKLDFLTRELKGKRPLVP--PYIE-VNADVKEVLDTFRVAAELGSDS 615
Tappc-bD_TraesCS3D02G005000_ DEEKKLDFLTRELKGKRPLVP--PYIE-VNADVKEVLDTFRVAAELGSDS 617

```
                                                                    Mg2+ binding*
                                                              Hydrophobic poket*
Tappc4B_TraesCS3B02G168000_       FGAYVISMATAPSDVLAVELLQRECGVK-----------KPMRVVPLFEK 601
Tappc4D_TraesCS3D02G150500_       FGAYVISMATAPSDVLAVELLQRECGVK-----------KPMRVVPLFEK 602
Tappc4A_TraesCS3A02G134200_       FGAYVISMATAPSDVLAVELLQRECGVK-----------TPMRVVPLFEK 602
Tappc1aA_TraesCS6A02G195600_      FGAYIISMATAPSDVLAVELLQRECHIK-----------KPLRVVPLFEK 565
Tappc1aD_TraesCS6D02G183200_      FGAYIISMATAPSDVLAVELLQRECHVK-----------KPLRVVPLFEK 565
Tappc1aB_TraesCS6B02G223100_      FGAYIISMATAPSDVLAVELLQRECHIK-----------KPLRVVPLFEK 565
Tappc1bA_TraesCS7A02G345400_      FGAYIISMATAPSDVLAVELLQRECHVG-----------HPLRVVPLFEK 564
Tappc1bD_TraesCS7D02G333900_      FGAYIISMATAPSDVLAVELLQRECHVG-----------HPLRVVPLFEK 564
Tappc2B_TraesCS5B02G179800_       FGAYVISMATSPSDVLAVELLQRECHIK-----------TPLRVVPLFEK 568
Tappc2D_TraesCS5D02G186200_       FGAYVISMATSPSDVLAVELLQRECHIK-----------TPLRVVPLFEK 568
Tappc2A_TraesCS5A02G181800_       FGAYVISMATSPSDVLAVELLQRECHIK-----------TPLRVVPLFEK 568
Tappc3A_TraesCS3A02G306700_       FGAYVISMATAPSDVLAVELLQRECHVK-----------KPLRVVPLFEK 562
Tappc3D_TraesCS3D02G295200_       FGAYVISMATAPSDVLAVELLQRECHVK-----------KPLRVVPLFEK 562
Tappc3B_TraesCS3B02G329800_       FGAYVISMATAPSDVLAVELLQRECHVK-----------KPLRVVPLFEK 562
Tappc-bB_TraesCS3B02G008500_      LGAYVISMASNASDVLAVELLQKDARLTVSGDLGRECPGGTLRVVPLFET 665
Tappc-bD_TraesCS3D02G005000_      LGAYVISMASNASDVLAVELLQKDARLTVSGDLGRECPGGTLRVVPLFET 667

                                                                        *Mg2+ binding
                                                   Monoubiqutination       *HCO3 binding
                                                                 *     +++++++++ subdomain II
Tappc4B_TraesCS3B02G168000_       LADLQQARATMELLFSIDWYKERIN----GKQEIMIGYSDSGKDAGRLSA 647
Tappc4D_TraesCS3D02G150500_       LADLQQARATMELLFSIDWYKERIN----GKQEIMIGYSDSGKDAGRLSA 648
Tappc4A_TraesCS3A02G134200_       LADLQQARATMELLFSIDWYKERID----GKQEIMIGYSDSGKDAGRLSA 648
Tappc1aA_TraesCS6A02G195600_      LADLEAAPAAVARLFSIDWYMDRIN----GKQEVMIGYSDSGKDAGRLSA 611
Tappc1aD_TraesCS6D02G183200_      LADLEAAPAAVARLFSIDWYMDRIN----GKQEVMIGYSDSGKDAGRLSA 611
Tappc1aB_TraesCS6B02G223100_      LADLEAAPAAVARLFSVDWYMDRIN----GKQEVMIGYSDSGKDAGRLSA 611
Tappc1bA_TraesCS7A02G345400_      LADLEAAPAAVARLFSIDWYMDRIG----GKQEVMIGYSDSGKDAGRLSA 610
Tappc1bD_TraesCS7D02G333900_      LADLEAAPAAVARLFSIDWYMDRIG----GKQEVMIGYSDSGKDAGRLSA 610
Tappc2B_TraesCS5B02G179800_       LADLEAAPAALARLFSIDWYRERIN----GKQEVMIGYSDSGKDAGRLSA 614
Tappc2D_TraesCS5D02G186200_       LADLEAAPAALARLFSIDWYRERIN----GKQEVMIGYSDSGKDAGRLSA 614
Tappc2A_TraesCS5A02G181800_       LADLEAAPAALARLFSIDWYRERIN----GKQEVMIGYSDSGKDAGRLSA 614
Tappc3A_TraesCS3A02G306700_       LADLEAAPAALARLFSVEWYRNRIN----GKQEVMIGYSDSGKDAGRFSA 608
Tappc3D_TraesCS3D02G295200_       LADLEAAPAALARLFSVEWYRNRIN----GKQEVMIGYSDSGKDAGRFSA 608
Tappc3B_TraesCS3B02G329800_       LADLEAAPAALARLFSVEWYRNRIN----GKQEVMIGYSDSGKDAGRFSA 608
Tappc-bB_TraesCS3B02G008500_      VKDLREAGSAIRKLLAIDWYREHIIKNHNGHQEVMVGYSDSGKDAGRFTA 715
```

```
Tappc-bD_TraesCS3D02G005000_      VKDLREAGSAIWKLLAIDWYREHIIKNHNGHQEVMVGYSDSGKDAGRFTA 717

                                                                *(PEP/Asp binding)
                                                    ************ subdomain III
Tappc4B_TraesCS3B02G168000_       AWYLYKAQEEIVDVAEQHGVKLTIFHGRGGTVGRGGGPSHLAILSQPPNT 697
Tappc4D_TraesCS3D02G150500_       AWYLYKAQEEIVDVAEQHGVKLTIFHGRGGTVGRGGGPSHLAILSQPPNT 698
Tappc4A_TraesCS3A02G134200_       AWYLYKAQEEIVDVAERHGVKLTIFHGRGGTVGRGGGPSHLAILSQPPNT 698
Tappc1aA_TraesCS6A02G195600_      AWQMYKAQEELIKVAKHYGVKLTMFHGRGGTVGRGGGPSHLAILSQPPDT 661
Tappc1aD_TraesCS6D02G183200_      AWQMYKAQEELIKVAKHYEVKLTMFHGRGGTVGRGGGPSHLAILSQPPDT 661
Tappc1aB_TraesCS6B02G223100_      AWQMYKAQEELIKVAKHYGVKLTMFHGRGGTVGRGGGPSHLAILSQPPDT 661
Tappc1bA_TraesCS7A02G345400_      AWQLYKAQEELVKVAKQYGVKLTMFHGRGGTVGRGGGPTHLAILSQPPET 660
Tappc1bD_TraesCS7D02G333900_      AWQLYKAQEELVKVAKQYGVKLTMFHGRGGTVGRGGGPTHLAILSQPPET 660
Tappc2B_TraesCS5B02G179800_       AWQMYKAQEDLVKVAKQFGVKLTMFHGRGGTVGRGGGPTHLAILSQPPDT 664
Tappc2D_TraesCS5D02G186200_       AWQMYKAQEDLVKVAKQFGVKLTMFHGRGGTVGRGGGPTHLAILSQPPDT 664
Tappc2A_TraesCS5A02G181800_       AWQMYKAQEDLVKVAKQFGVKLTMFHGRGGTVGRGGGPTHLAILSQPPDT 664
Tappc3A_TraesCS3A02G306700_       GWQLYKAQEELIKVAKAFGIKLTMFHGRGGTVGRGGGPTHLAILSQPPET 658
Tappc3D_TraesCS3D02G295200_       GWQLYKAQEELIKVAKAFGIKLTMFHGRGGTVGRGGGPTHLAILSQPPET 658
Tappc3B_TraesCS3B02G329800_       GWQLYKAQEELIKVAKAFGIKLTMFHGRGGTVGRGGGPTHLAILSQPPET 658
Tappc-bB_TraesCS3B02G008500_      AWELYKAQEDVVAACNEHGIKVTLFHGRGGSIGRGGGPTYLAIQSXPPGS 765
Tappc-bD_TraesCS3D02G005000_      AWELYKAQEDVVAACNEHGIKVTLFHGRGGSIGRGGGPTYLAIQSQPPGS 767

Tappc4B_TraesCS3B02G168000_       VNGSLRVTVQGEVIEKSFGEDNLCFRTLQRFTAATLEHGMNPPVSPK-PE 746
Tappc4D_TraesCS3D02G150500_       VNGSLRVTVQGEVIEKSFGEDNLCFRTLQRFTAATLEHGMNPPVSPK-PE 747
Tappc4A_TraesCS3A02G134200_       VNGSLRVTVQGEVIEKSFGEDNLCFRTLQRFTAATLEHGMNPPVSPK-PE 747
Tappc1aA_TraesCS6A02G195600_      IHGSLRVTVQGEVIEHSFGEEHLCFRTLQRFTAATLEHGMHPPISPK-PE 710
Tappc1aD_TraesCS6D02G183200_      IHGSLRVTVQGEVIEHSFGEEHLCFRTLQRFTAATLEHGMHPPISPK-PE 710
Tappc1aB_TraesCS6B02G223100_      IHGSLRVTVQGEVIEHSFGEEHLCFRTLQRFTAATLEHGMHPPISPK-PE 710
Tappc1bA_TraesCS7A02G345400_      VNGSLRVTVQGEVIEHSFGEEHLCFRTLQRFTAATLEHGMHPPVSPK-PE 709
Tappc1bD_TraesCS7D02G333900_      VNGSLRVTVQGEVIEHSFGEEHLCFRTLQRFTAATLEHGMHPPVSPK-PE 709
Tappc2B_TraesCS5B02G179800_       INGSLRVTVQGEVIEQSFGEEHLCFRTLQRFTAATLEHGMRPPISPK-PE 713
Tappc2D_TraesCS5D02G186200_       INGSLRVTVQGEVIEQSFGEEHLCFRTLQRFTAATLEHGMRPPISPK-PE 713
Tappc2A_TraesCS5A02G181800_       INGSLRVTVQGEVIEQSFGEEHLCFRTLQRFTAATLEHGMRPPISPK-PE 713
Tappc3A_TraesCS3A02G306700_       IHGSLRVTVQGEVIEQSFGEEHLCFRTLQRFTAATLEHGMHPPIAPK-PE 707
Tappc3D_TraesCS3D02G295200_       IHGSLRVTVQGEVIEQSFGEEHLCFRMLQRFTAATLEHGMHPPIAPK-PE 707
Tappc3B_TraesCS3B02G329800_       IHGSLRVTVQGEVIEQSFGEEHLCFRTLQRFTAATLEHGMHPPIAPK-PE 707
Tappc-bB_TraesCS3B02G008500_      VMGTLRSTEQGEMVQAKFGLPQTAVRQLEIYTTAVLLATLRPPQPPRDPN 815
Tappc-bD_TraesCS3D02G005000_      VMGTLRSTEQGEMVQAKFGLPQTAVRQLEIYTTAVLLATLRPPQPPRDPN 817
```

```
                                                                                  +++
Tappc4B_TraesCS3B02G168000_        WRALLDDMATVATEEYRSIVFQEPRFVEYFRSATPETEYGRMNIGSRPSK 796
Tappc4D_TraesCS3D02G150500_        WRALLDDMATVATEEYRSIVFQEPRFVEYFRSATPETEYGRMNIGSRPSK 797
Tappc4A_TraesCS3A02G134200_        WRALLDDMATVATEEYRAIVFQEPRFVEYFRCATPETEYGRMNIGSRPSK 797
Tappc1aA_TraesCS6A02G195600_       WRALMDEMAVVATKEYRSIVFQEPRFVEYFRSATPETEYGRMNIGSRPSK 760
Tappc1aD_TraesCS6D02G183200_       WRALMDEMAVVATKEYRSIVFQEPRFVEYFRSATPETEYGRMNIGSRPSK 760
Tappc1aB_TraesCS6B02G223100_       WRALMDEMAVVATKEYRSIVFQEPRFVEYFRSATPETEYGRMNIGSRPSK 760
Tappc1bA_TraesCS7A02G345400_       WRALMDEMAVVATEEYRAMVFKEPRFVEYFRSATPETEYGRMNIGSRPSK 759
Tappc1bD_TraesCS7D02G333900_       WRALMDEMAVVATEEYRAMVFKEPRFVEYFRSATPETEYGRMNIGSRPSK 759
Tappc2B_TraesCS5B02G179800_        WRALLDEMAVVATEEYRSIVFQEPRFVEYFRLATPETEYGRMNIGSRPSK 763
Tappc2D_TraesCS5D02G186200_        WRALLDEMAVVATEEYRSIVFQEPRFVEYFRLATPETEYGRMNIGSRPSK 763
Tappc2A_TraesCS5A02G181800_        WRALLDEMAVVATEEYRSIVFQEPRFVEYFRLATPETEYGRMNIGSRPSK 763
Tappc3A_TraesCS3A02G306700_        WRALMDEMAVVATEEYRSIVFQEPRFVEYFRLATPELEYGRMNIGSRPSK 757
Tappc3D_TraesCS3D02G295200_        WRALMDEMAVVATEEYRSIVFQEPRFVEYFRLATPELEYGRMNIGSRPSK 757
Tappc3B_TraesCS3B02G329800_        WRALMDEMAVVATEEYRSIVFQEPRFVEYFRLATPELEYGRMNIGSRPSK 757
Tappc-bB_TraesCS3B02G008500_       WRHVMEEISRASCAHYRRTVYEDPAFVTYFQEATPQAELGYLNIGSRPAK 865
Tappc-bD_TraesCS3D02G005000_       WRHVMEEISRASCAHYRRTVYEDPAFVTYFQEATPQAELGYLNIGSRPAK 867

                                   ++    ++++ (bicarbonate-binding)
                                                *(PEP binding)
                                                        *(S/A755)
Tappc4B_TraesCS3B02G168000_        RK----PGGGIESLRAIPWIFAWTQTRFHLPVWLGFGAAFRHAMDK---P 839
Tappc4D_TraesCS3D02G150500_        RK----PGGGIESLRAIPWIFAWTQTRFHLPVWLGFGAAFRHAMDK---P 840
Tappc4A_TraesCS3A02G134200_        RK----PGGGIESLRAIPWIFAWTQTRFHLPVWLGFGAAFRHAMDR---P 840
Tappc1aA_TraesCS6A02G195600_       RK----PSGGIESLRAIPWIFAWTQTRFHLPVWLGFGAAFKHIIQKD--I 804
Tappc1aD_TraesCS6D02G183200_       RK----PSGGIESLRAIPWIFAWTQTRFHLPVWLGFGAAFKHIIQKD--I 804
Tappc1aB_TraesCS6B02G223100_       RK----PSGGIESLRAIPWIFAWTQTRFHLPVWLGFGAAFKHIIQKD--I 804
Tappc1bA_TraesCS7A02G345400_       RK----PSGGIETLRAIPWIFAWTQTRFHLPVWLGFGAAFKHIMQKD--I 803
Tappc1bD_TraesCS7D02G333900_       RK----PSGGIETLRAIPWIFAWTQTRFHLPVWLGFGAAFKHIMQKD--I 803
Tappc2B_TraesCS5B02G179800_        RK----PSGGIESLRAIPWIFAWTQTRFHLPVWLGFGGAFKHILKKD--I 807
Tappc2D_TraesCS5D02G186200_        RK----PSGGIESLRAIPWIFAWTQTRFHLPVWLGFGGAFKHILKKD--I 807
Tappc2A_TraesCS5A02G181800_        RK----PSGGIESLRAIPWIFAWTQTRFHLPVWLGFGGAFKHILKKD--I 807
Tappc3A_TraesCS3A02G306700_        RK----PSGGIESLRAIPWIFAWTQTRFHLPVWLGFGAAFKHVLQKD--I 801
Tappc3D_TraesCS3D02G295200_        RK----PSGGIESLRAIPWIFAWTQTRFHLPVWLGFGAAFKHVLQKD--I 801
Tappc3B_TraesCS3B02G329800_        RK----PSGGIESLRAIPWIFAWTQTRFHLPVWLGFGAAFKHVLQKD--I 801
Tappc-bB_TraesCS3B02G008500_       RKAAITAAGGIASLRAIPWVFAWTQTRLALPAWLGVGTGLQDARDKG--- 912
Tappc-bD_TraesCS3D02G005000_       RKAAITAAGGIASLRAIPWVFAWTQTRLALPAWLGVGTGLQDARDKG--- 914
```

```
                                         (Asp binidng site)*
Tappc4B_TraesCS3B02G168000_     GGLATLREMYDEWPFFRVTIDLLEMVFAKGDPGIAALYDKLLVPQD-LWP 888
Tappc4D_TraesCS3D02G150500_     GGLATLREMYDEWPFFRVTIDLLEMVFAKGDPGIAALYDKLLVPQD-LWP 889
Tappc4A_TraesCS3A02G134200_     GGLATLREMYDEWPFFRVTIDLLEMVFAKGDPGIAALYDKLLVPED-LWP 889
Tappc1aA_TraesCS6A02G195600_    RNIHTLKEMYNEWPFFRVTLDLLEMVFAKGDPGIAALYDKLLVAED-LQS 853
Tappc1aD_TraesCS6D02G183200_    RNIHTLKEMYNEWPFFRVTLDLLEMVFAKGDPGIAALYDKLLVAED-LQS 853
Tappc1aB_TraesCS6B02G223100_    RNIHTLKEMYNEWPFFRVTLDLLEMVFAKGDPGIAALYDKLLVAED-LQS 853
Tappc1bA_TraesCS7A02G345400_    RNVQALREMYNEWPFFRVTLDLLEMVFAKGDPGIAALYDELLVADE-LKP 852
Tappc1bD_TraesCS7D02G333900_    RNVQALREMYNEWPFFRVTLDLLEMVFAKGDPGIAALYDELLVADE-LKP 852
Tappc2B_TraesCS5B02G179800_     RNFHMLQEMYNEWPFFRVTIDLVEMVFAKGNPGIAALYDRLLVSEG-LQP 856
Tappc2D_TraesCS5D02G186200_     RNFHMLQEMYNEWPFFRVTIDLVEMVFAKGNPGIAALYNRLLVSEG-LQP 856
Tappc2A_TraesCS5A02G181800_     RNFHMLQEMYNEWPFFRVTIDLVEMVFAKGNPGIAALYDRLLVSEG-LQP 856
Tappc3A_TraesCS3A02G306700_     RNLQALKEMYNEWPFFRVTIDLVEMVFAKGDPGIAALYDKLLVSDD-LWP 850
Tappc3D_TraesCS3D02G295200_     RNLQALKEMYNEWPFFRVTIDLVEMVFAKGDPGIAALYDKLLVSDD-LWP 850
Tappc3B_TraesCS3B02G329800_     RNLQTLKEMYNEWPFFRVTIDLVEMVFAKGDPGIAALYDKLLVSDD-LWP 850
Tappc-bB_TraesCS3B02G008500_    -RTEDLRAMYEEWPFFQSTLDLIEMVVAKADAPMAKHYDDVLVLSPERRA 961
Tappc-bD_TraesCS3D02G005000_    -RTEDLRAMYEEWPFFQSTLDLIEMVVAKADAPMAKHYDDVLVLSPERRA 963

                                                       (Asp binidng site)*
Tappc4B_TraesCS3B02G168000_     FGEQLRANYAETQSLLLKVAGHEDLLESDPYLRQRLLLRDSYITALNVCQ 938
Tappc4D_TraesCS3D02G150500_     FGEQLRANYAETQSLLLKVAGHEDLLESDPYLRQRLLLRDSYITALNVCQ 939
Tappc4A_TraesCS3A02G134200_     FGEQLRANYAETQSLLLKVAGHEDLLESDPYLRQRLLLRDSYITALNVCQ 939
Tappc1aA_TraesCS6A02G195600_    FGEQLRQNFEETKQLLLQVAGHKDVLEGDPYLKQRLRLRESYITTLNVCQ 903
Tappc1aD_TraesCS6D02G183200_    FGEQLRQNFEETKRLLLQVAGHKDVLEGDPYLKQRLRLRESYITTLNVCQ 903
Tappc1aB_TraesCS6B02G223100_    FGEQLRQNFEETKQLLLQVAGHKDVLEGDPYLKQRLRLRESYITTLNVCQ 903
Tappc1bA_TraesCS7A02G345400_    LGEQLRSNFEDTKKLLVQVAGHRDVLEDDPYLKQRLRLRDPYITTLNVCQ 902
Tappc1bD_TraesCS7D02G333900_    LGEQLRSNFEDTKKLLVQVAGHRDVLEDDPYLKQRLRLRDPYITTLNVCQ 902
Tappc2B_TraesCS5B02G179800_     LGEKLRANYEETQKLLLQVAGHKDLLEGDPYLKQRLRLRDAYITTMNVCQ 906
Tappc2D_TraesCS5D02G186200_     LGEKLRANYEETQKLLLQVAGHKDLLEGDPYLKQRLRLRDAYITTMNVCQ 906
Tappc2A_TraesCS5A02G181800_     LGEKLRANYEETQKLLLQVAGHKDLLEGDPYLKQRLRLRDAYITTMNVCQ 906
Tappc3A_TraesCS3A02G306700_     FGERLRANYEETKQLLLQVAGHKDLLEGDPYLRQSLRLRDSYITTLNVCQ 900
Tappc3D_TraesCS3D02G295200_     FGERLRANYEETKQLLLQVAGHKDLLEGDPYLRQSLRLRDSYITTLNVCQ 900
Tappc3B_TraesCS3B02G329800_     FGERLRANYEETKQLLLQVAGHKDLLEGDPYLRQSLRLRDSYITTLNVCQ 900
Tappc-bB_TraesCS3B02G008500_    LGEELRRELARAGSCVLAVSGHTKLSANNRSLRRLIESRLPYLNPMNMLQ 1011
Tappc-bD_TraesCS3D02G005000_    LGEELRRELARAGSCVLAVSGHTKLSANNRSLRRLIESRLPYLNPMNMLQ 1013

Tappc4B_TraesCS3B02G168000_     AYTLKRIRDGEFRPAT-RPPLSKEFIDET---AESLMELNPSSEYDPGLE 984
Tappc4D_TraesCS3D02G150500_     AYTLKRIRDGEFRPAT-RPPLSKEFIDET---AESLMELNPSSEYDPGLE 985
```

```
Tappc4A_TraesCS3A02G134200_       AYTLKRIRDGEFRPAT-RPPLSKEFIDET---AESLMELNPSSEYDPGLE 985
Tappc1aA_TraesCS6A02G195600_      AYTLKRIRDPSFEVTPQQPPLSKEFSD-KEP--AELVQLNRGSEYAPGLE 950
Tappc1aD_TraesCS6D02G183200_      AYTLKRIRDPSFEVTPQQPPLSKEFSD-KEP--AELVQLNRGSEYAPGLE 950
Tappc1aB_TraesCS6B02G223100_      AYTLKRIRDPSFEVTPQQPPLSKEFCD-KEP--AELVQLNRGSEYAPGLE 950
Tappc1bA_TraesCS7A02G345400_      AYTLKRIRDPSFQVTA-QPPLSKEFADENQP--ASLVKLNAASEYAPGLE 949
Tappc1bD_TraesCS7D02G333900_      AYTLKRIRDPSFQVTA-QPPLSKEFADENQP--ASLVKLNAASEYAPGLE 949
Tappc2B_TraesCS5B02G179800_       AYTLKRIRDPDYHVAL-RPHLSKEVMDTSKP-AAELVTLNPASEYAPGLE 954
Tappc2D_TraesCS5D02G186200_       AYTLKRIRDPDYHVAL-RPHLSKEVMDTSKP-AAELVTLNPASEYAPGLE 954
Tappc2A_TraesCS5A02G181800_       AYTLKRIRDPDYHVAL-RPHLSKEVMDTSKP-AAELVTLNPASEYAPGLE 954
Tappc3A_TraesCS3A02G306700_       AYTLKRIRDPSFHSQP-GPHLSKEIMESGKL-AAELLKLNPTSEYAPGLE 948
Tappc3D_TraesCS3D02G295200_       AYTLKRIRDPSFHSQP-GPHLSKEIMESGKL-AAELLKLNPTSEYAPGLE 948
Tappc3B_TraesCS3B02G329800_       AYTLKRIRDPSFHSQS-GPHLSKEIMESGKS-AAELVKLNPTSEYAPGLE 948
Tappc-bB_TraesCS3B02G008500_      VEVLRRLRRD--------DDNHK-------------------------LR 1028
Tappc-bD_TraesCS3D02G005000_      VEVLRRLRRD--------DDNHK-------------------------LR 1030

                                                *(Asp binidng site)
Tappc4B_TraesCS3B02G168000_       DTLILTMKGIAAGMQNTG 1002
Tappc4D_TraesCS3D02G150500_       DTLILTMKGIAAGMQNTG 1003
Tappc4A_TraesCS3A02G134200_       DTLILTMKGIAAGMQNTG 1003
Tappc1aA_TraesCS6A02G195600_      DTLILTMKGIAAGMQNTG 968
Tappc1aD_TraesCS6D02G183200_      DTLILTMKGIAAGMQNTG 968
Tappc1aB_TraesCS6B02G223100_      DTLILTMKGIAAGMQNTG 968
Tappc1bA_TraesCS7A02G345400_      DTLILTMKGIAAGMQNTG 967
Tappc1bD_TraesCS7D02G333900_      DTLILTMKGIAAGMQNTG 967
Tappc2B_TraesCS5B02G179800_       DTLILTMKGIAAGLQNTG 972
Tappc2D_TraesCS5D02G186200_       DTLILTMKGIAAGLQNTG 972
Tappc2A_TraesCS5A02G181800_       DTLILTMKGIAAGLQNTG 972
Tappc3A_TraesCS3A02G306700_       DTLILTMKGIAAGMQNTG 966
Tappc3D_TraesCS3D02G295200_       DTLILTMKGIAAGMQNTG 966
Tappc3B_TraesCS3B02G329800_       DTLILTMKGIAAGMQNTG 966
Tappc-bB_TraesCS3B02G008500_      DVLLITINGIAAGMRNTG 1046
Tappc-bD_TraesCS3D02G005000_      DVLLITINGIAAGMRNTG 1048
```

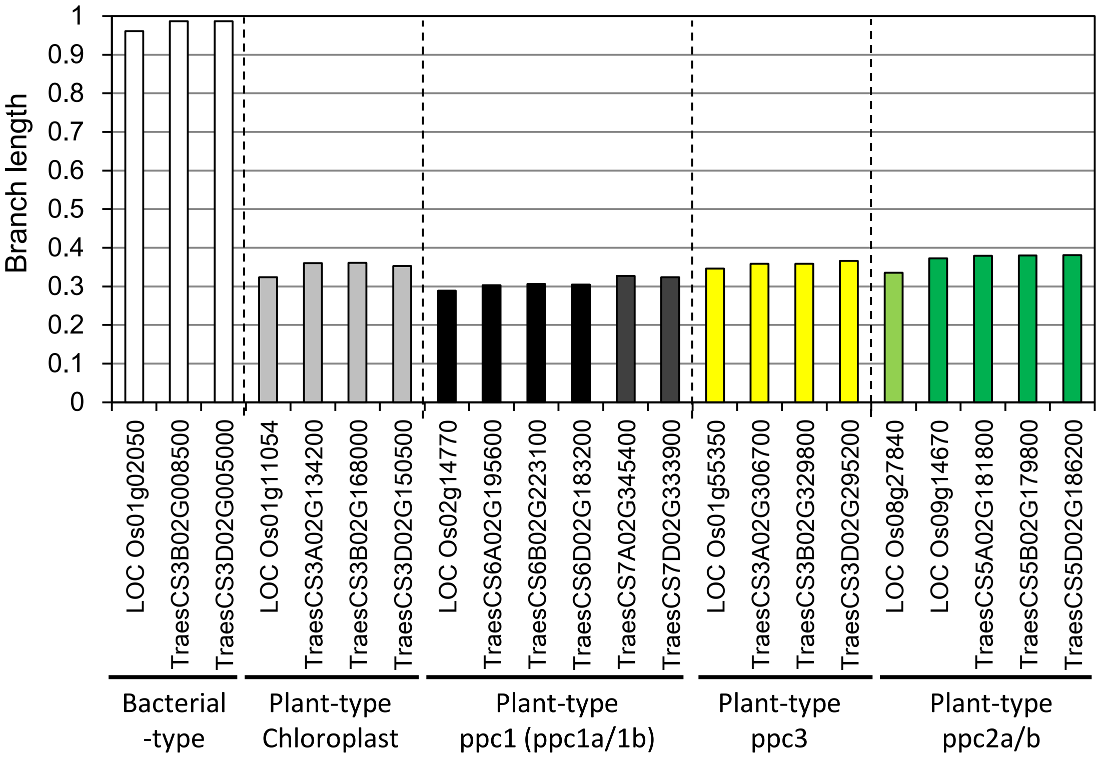

Supplementary Figure S4. Distributions of branch lengths for wheat PEPCs. Rice PEPCs were analyzed as references.

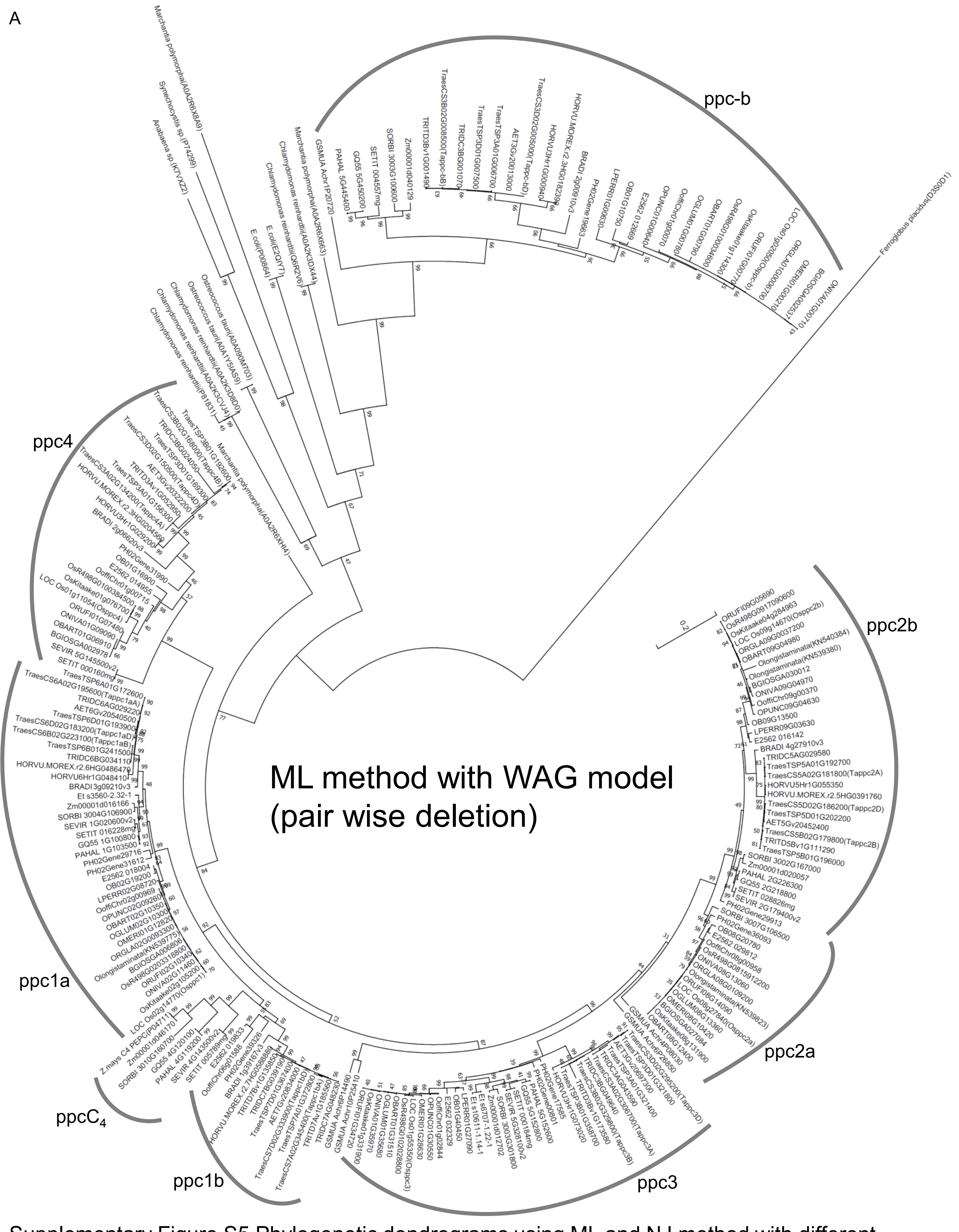

Supplementary Figure S5 Phylogenetic dendrograms using ML and NJ method with different parameters. (A-B) ML method, (C-J) Neighbor-joining method.

B

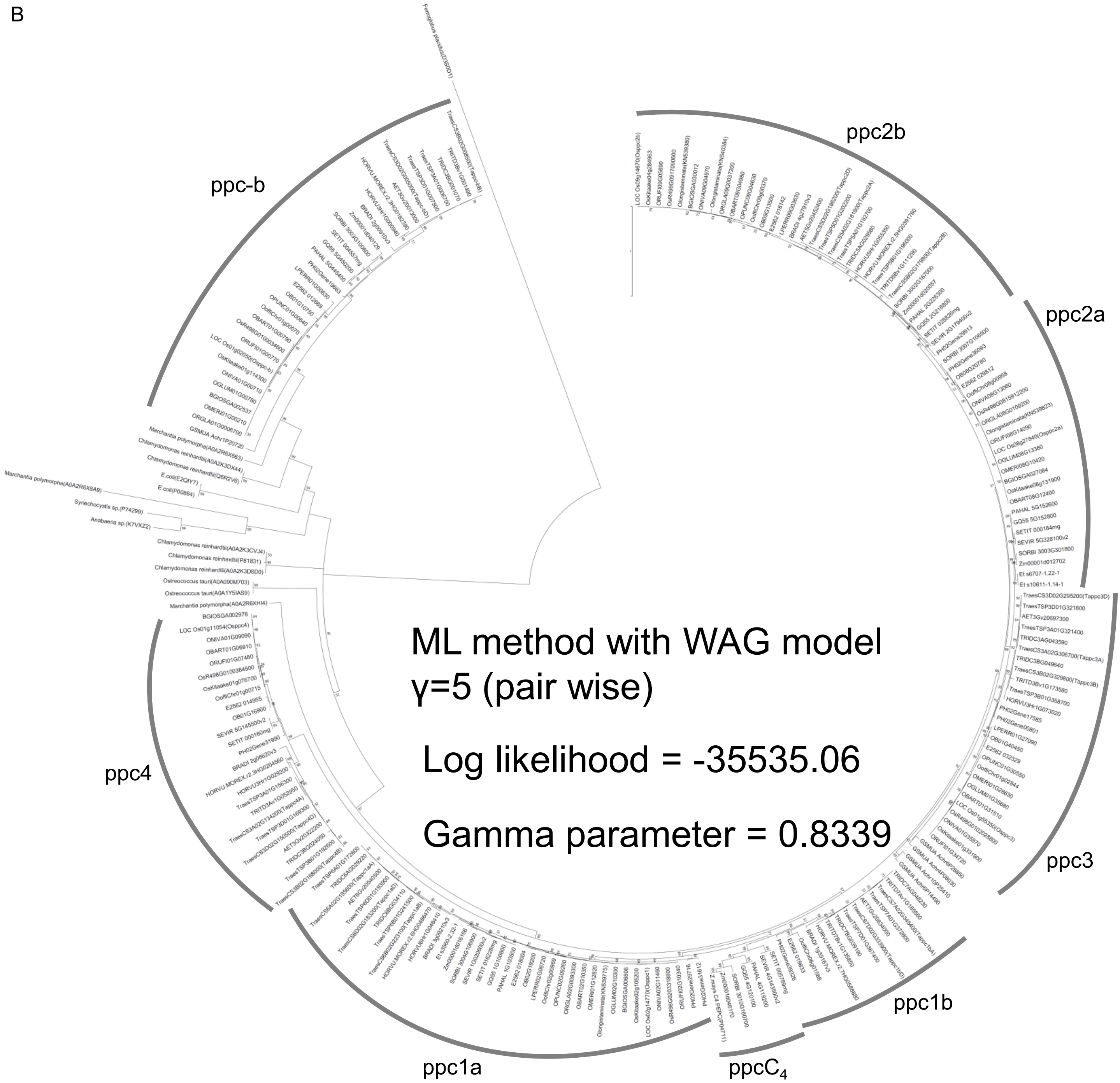

Supplementary Figure S5. Phylogenetic dendrograms using ML and NJ method with different parameters. (A-B) ML method, (C-J) Neighbor-joining method.

C

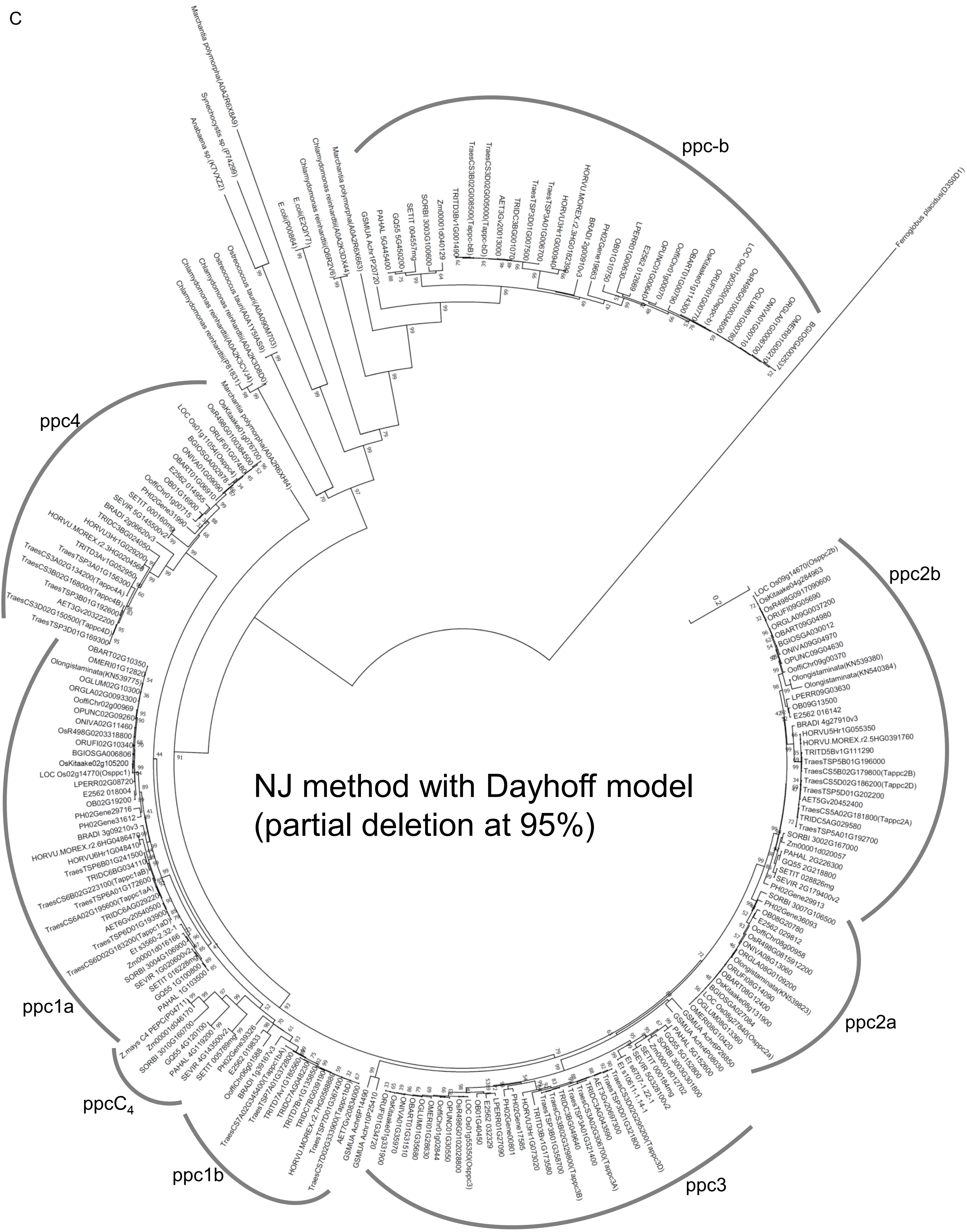

Supplementary Figure S5. Phylogenetic dendrograms using ML and NJ method with different parameters. (A-B) ML method, (C-J) Neighbor-joining method.

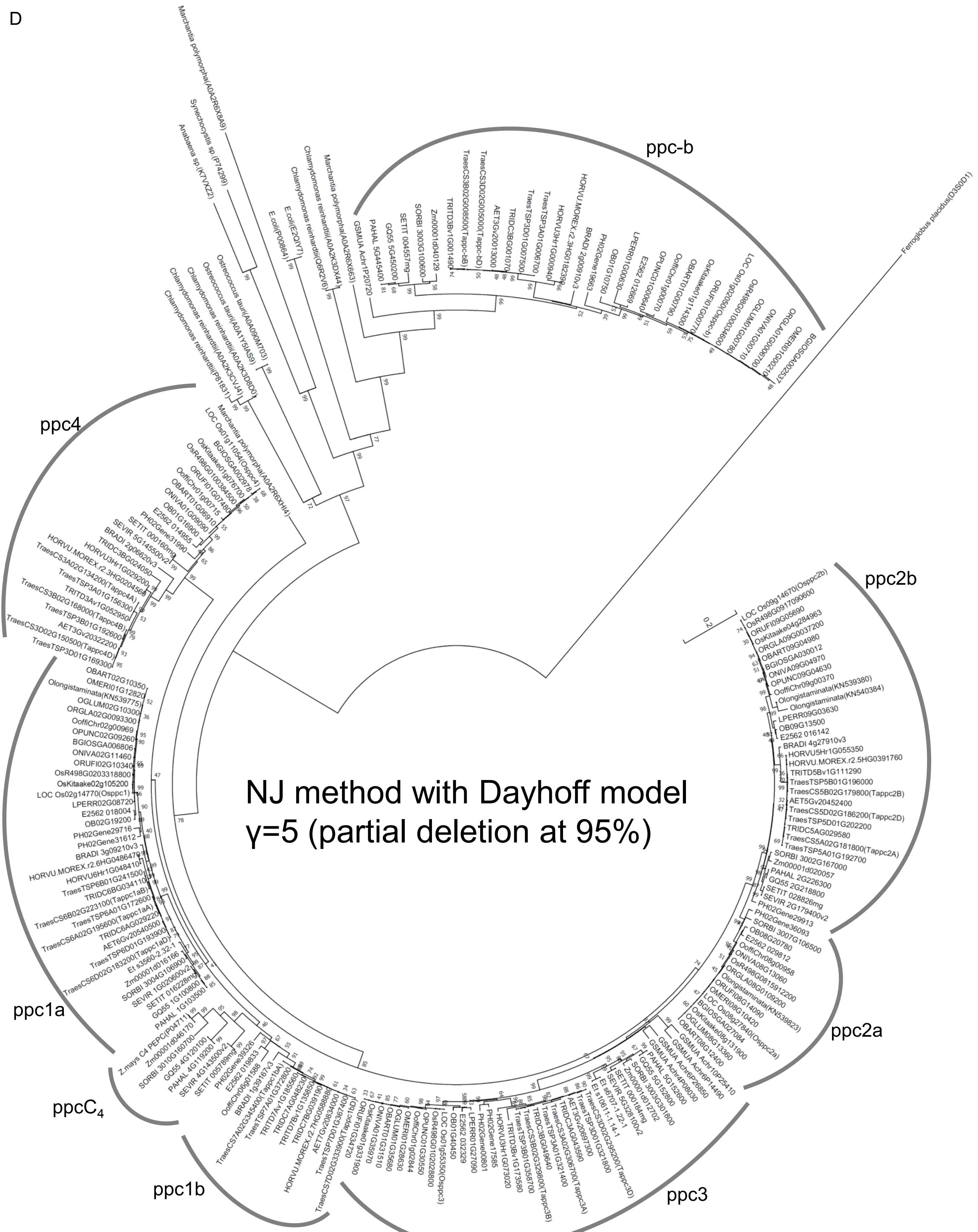

Supplementary Figure S5. Phylogenetic dendrograms using ML and NJ method with different parameters. (A-B) ML method, (C-J) Neighbor-joining method.

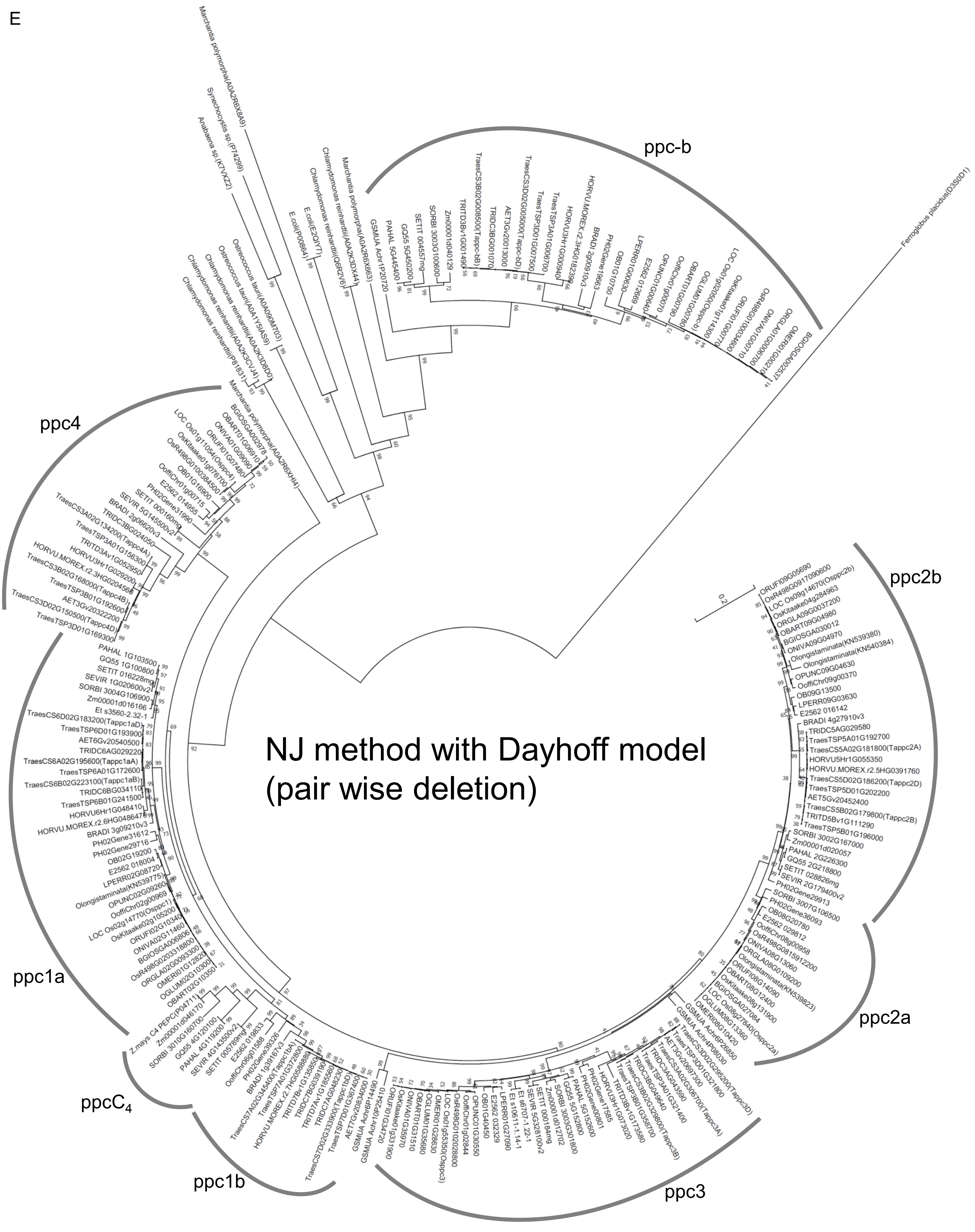

Supplementary Figure S5. Phylogenetic dendrograms using ML and NJ method with different parameters. (A-B) ML method, (C-J) Neighbor-joining method.

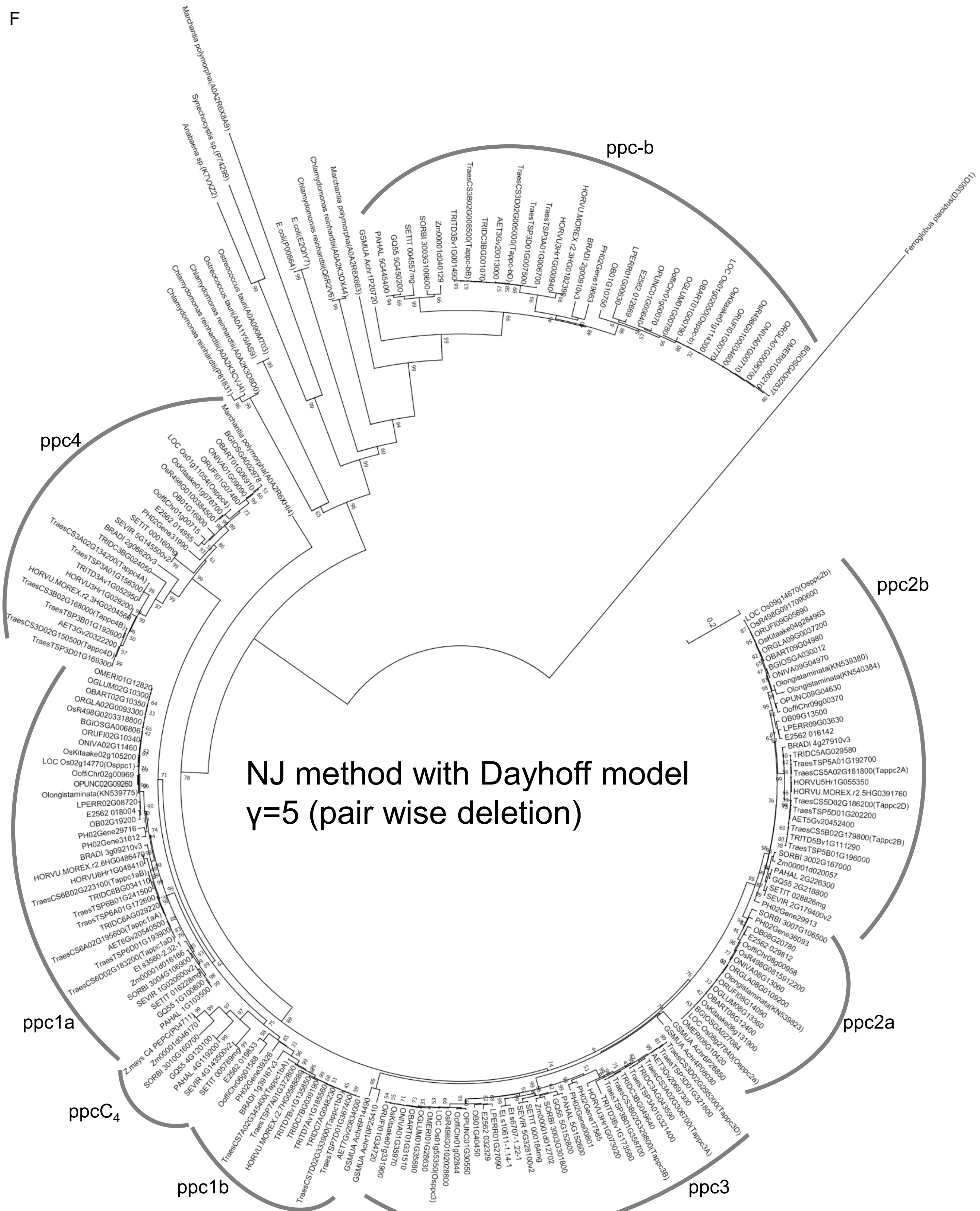

Supplementary Figure S5. Phylogenetic dendrograms using ML and NJ method with different parameters. (A-B) ML method, (C-J) Neighbor-joining method.

G

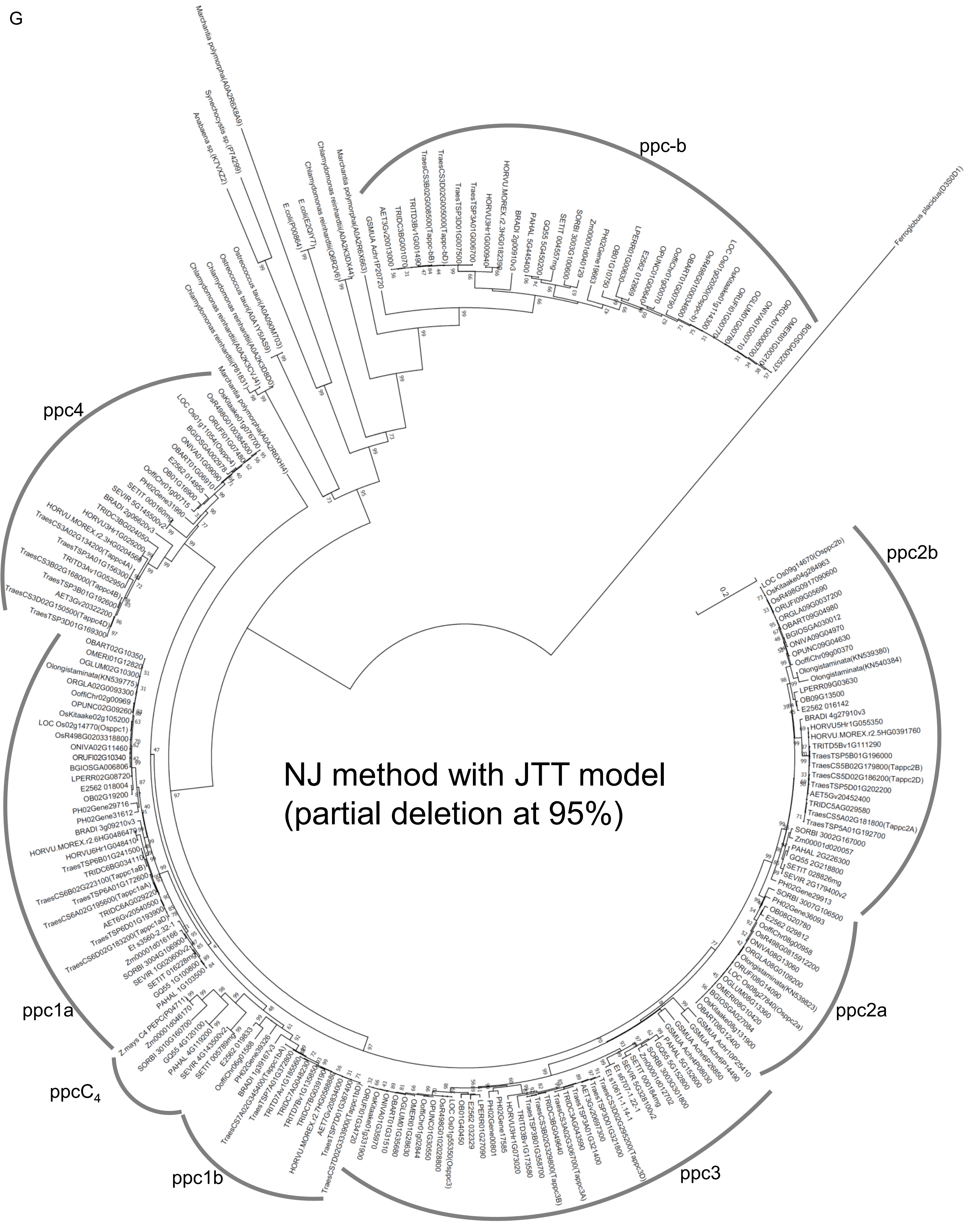

Supplementary Figure S5. Phylogenetic dendrograms using ML and NJ method with different parameters. (A-B) ML method, (C-J) Neighbor-joining method.

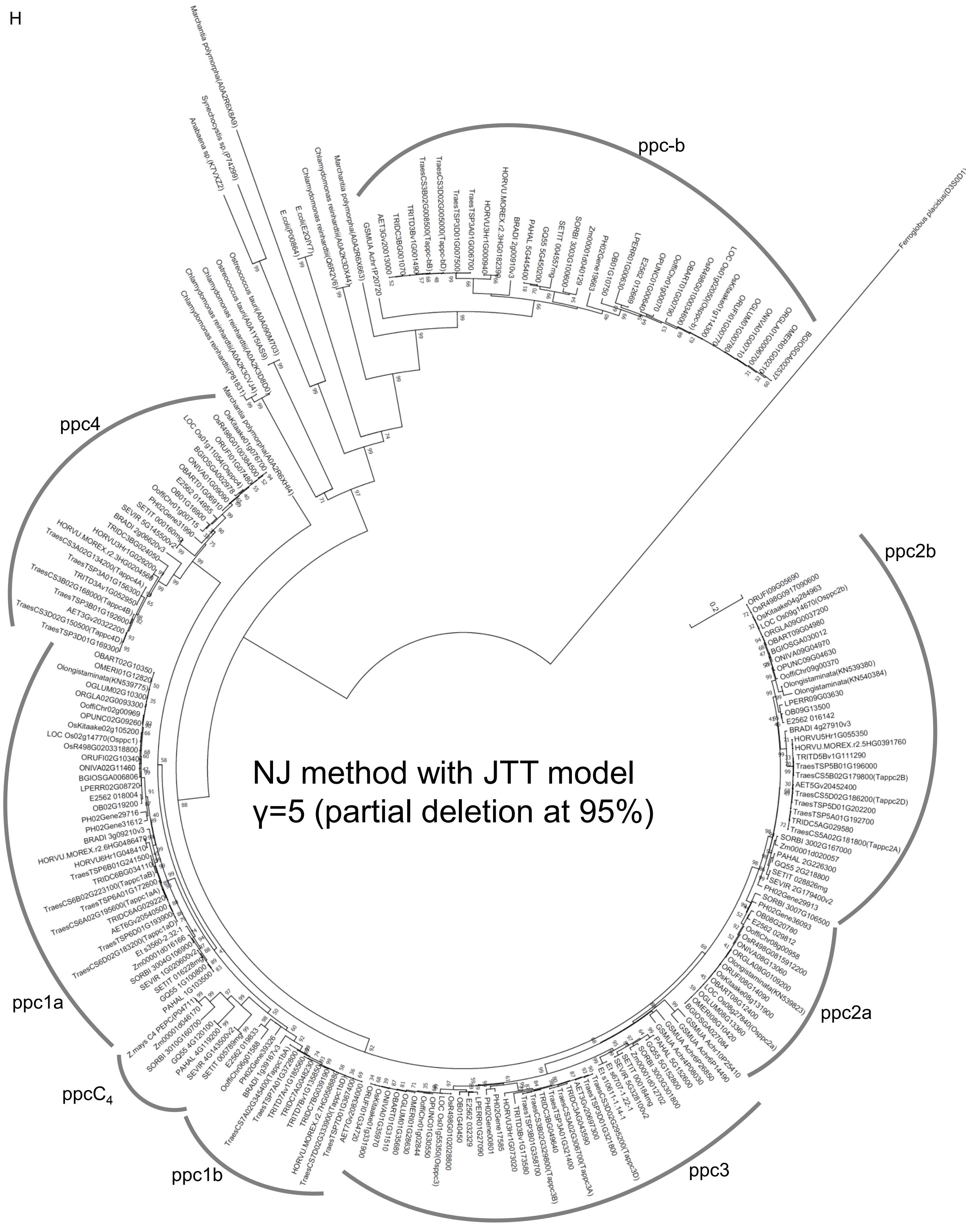

Supplementary Figure S5. Phylogenetic dendrograms using ML and NJ method with different parameters. (A-B) ML method, (C-J) Neighbor-joining method.

I

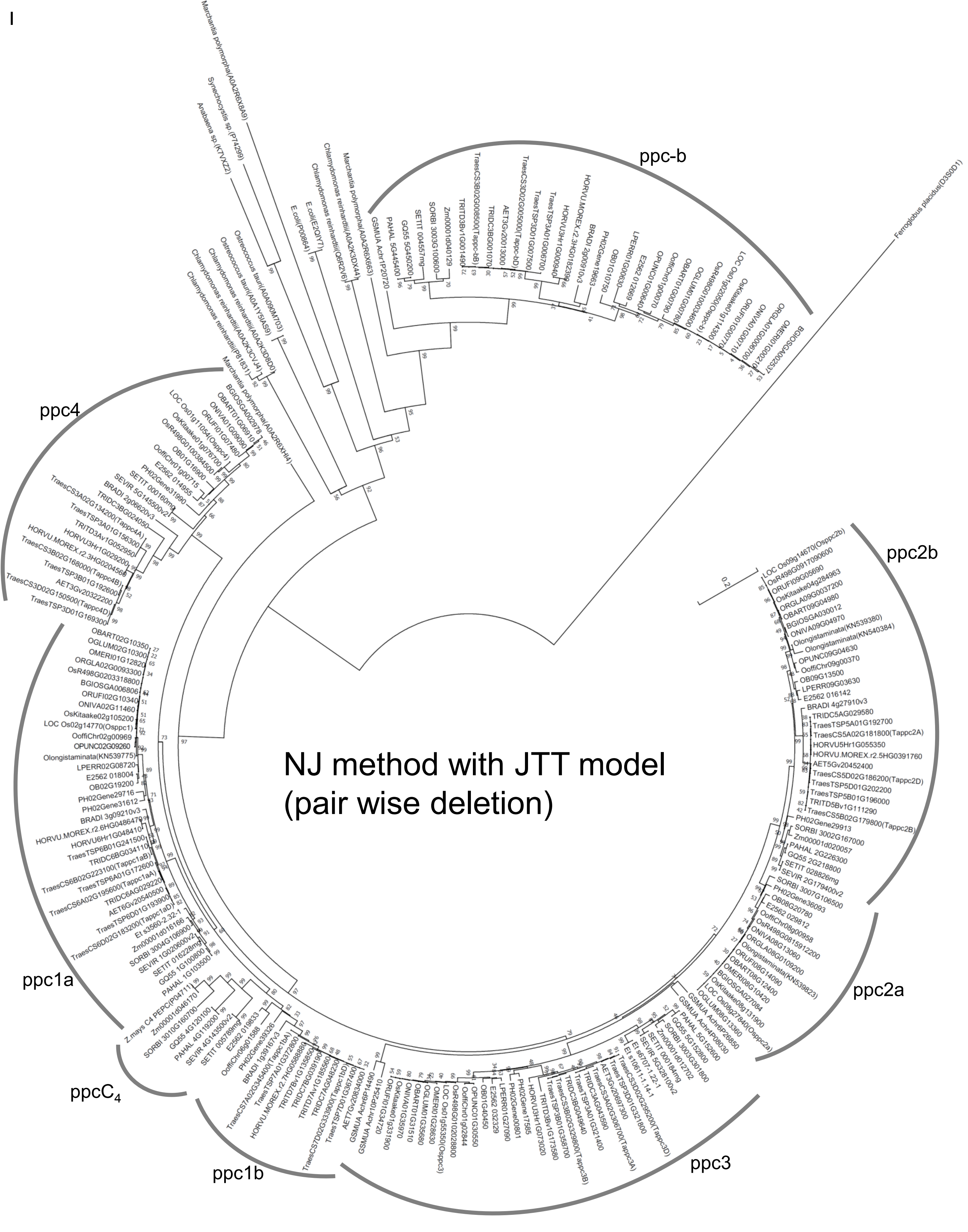

Supplementary Figure S5. Phylogenetic dendrograms using ML and NJ method with different parameters. (A-B) ML method, (C-J) Neighbor-joining method.

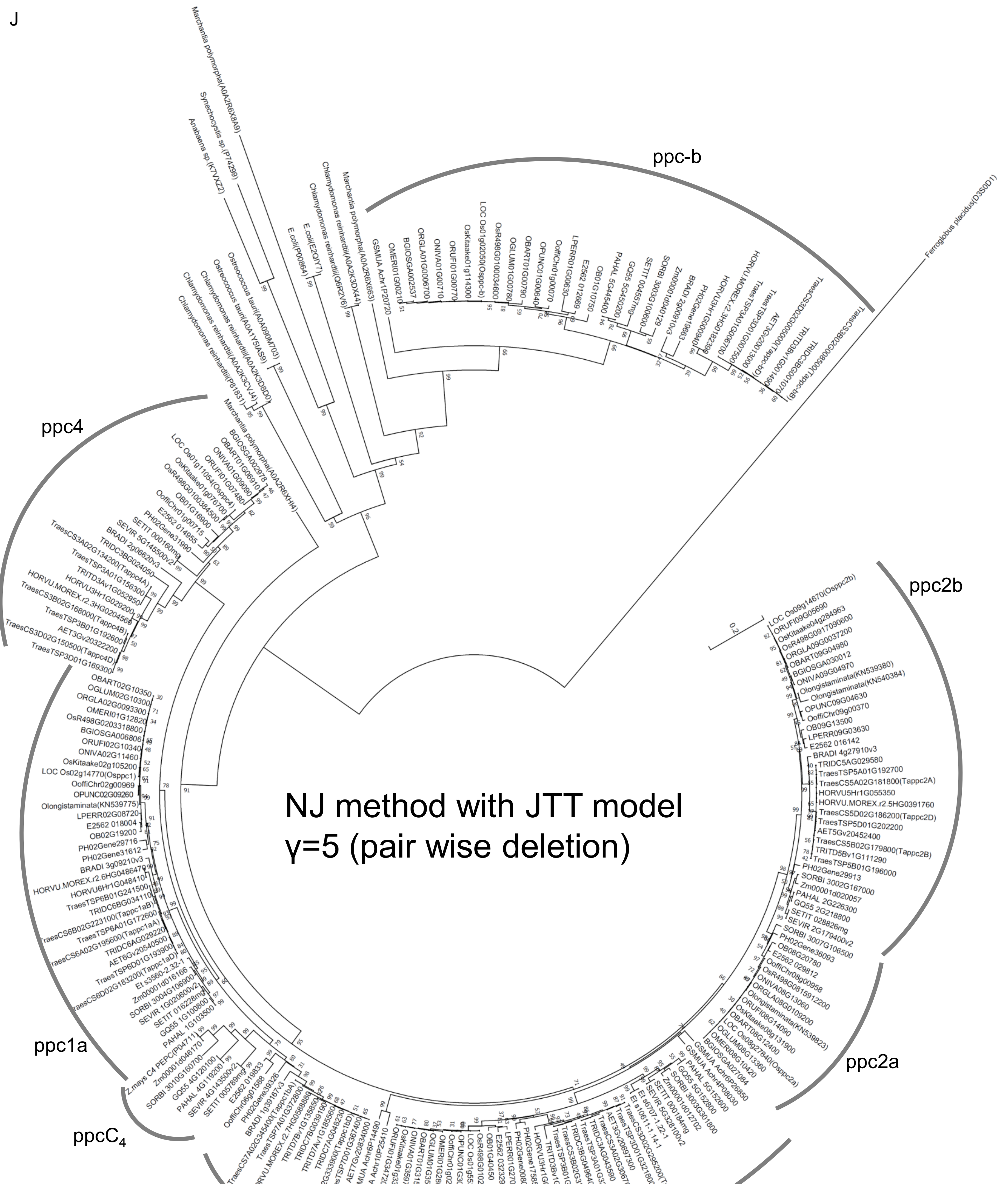

Supplementary Figure S5. Phylogenetic dendrograms using ML and NJ method with different parameters. (A-B) ML method, (C-J) Neighbor-joining method.

Genomic structure of ppc1b locus in *Oryza officinalis* and *Oryza punctata*

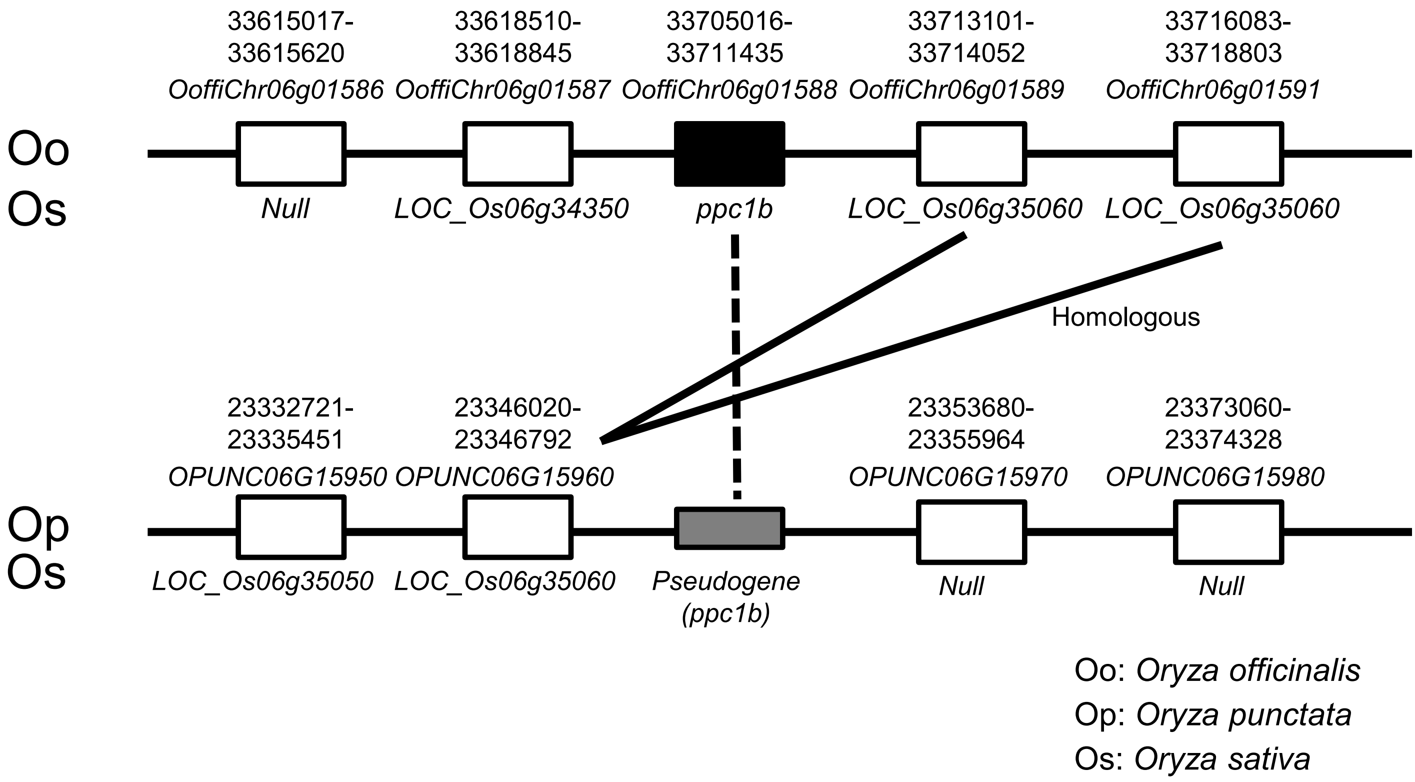

Sequence alignment of a Tappc1bD and the gene loss region in *O. punctata*

```
Query  98        ASSFSHMLNLANLAEEVQIANRRRNKLKRGDFADEASATTESDIEETLKRLV--------  149
                 A S SHMLNLANLAE                    ASATTESDIEETL+R
Sbjct  23350624  APSLSHMLNLANLAE--------------------ASATTESDIEETLRRRGVRRPRLHR  23350505

Query  150       ------------SDLGKTREEVFDALKNQTVDLVFTAHPTQSIRRS------LLQKHGR  190
                             S  G  R      L++   D +        S   S      + +K  R
Sbjct  23350504  PPPPSTSGGPSSRSTPGHCR-----FLQSINCDQI*NPFGCISFEFSAAFDAHMERKVCR  23350340
                                                                  Mutated region
Query  191       IRNCLRQLYAKDITADDKQELDEALQREIQAAF  223
                 IRNCL QLY KDITADDKQELDE LQRE+Q  F
Sbjct  23350339  IRNCLTQLYTKDITADDKQELDEPLQREVQFKF  23350241
```

Supplementary Figure S6. Genomic structure of ppc1b locus in *Oryza officinalis* and *Oryza punctata*.

Chromosomal localization of *ppc1b* on wheat and barley genomes

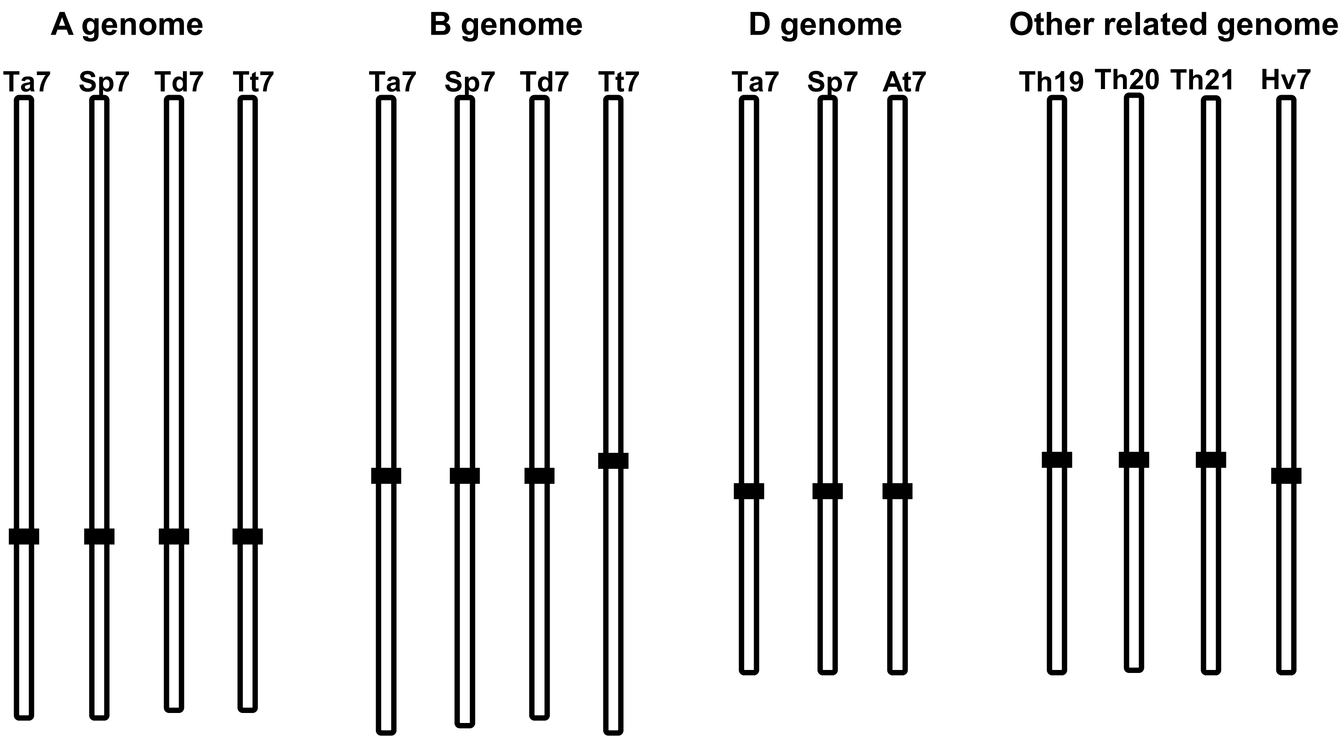

Ta: *Triticum aestivum*

Sp: *Triticum spelta*

Td: *Triticum dicoccoides*

Tt: *Triticum turgidum*

At: *Aegilops tauschii*

Th: *Thinopyrum intermedium*

Hv: *Hordeum vulgare*

ppc1b

Supplementary Figure S7. Chromosomal localization of ppc1b on wheat and barley genomes.

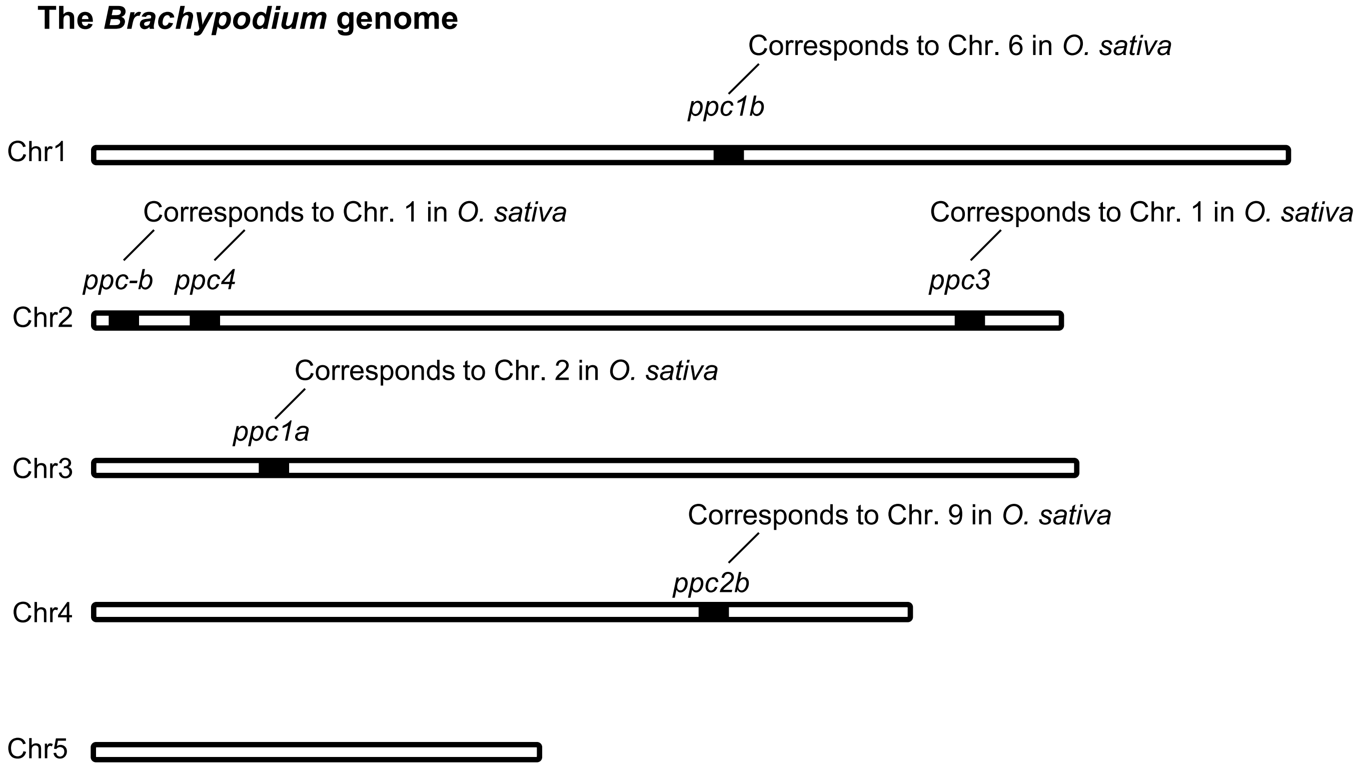

Supplementary Figure S8. Chromosomal localization of ppc1b on the *Brachypodium* genome.

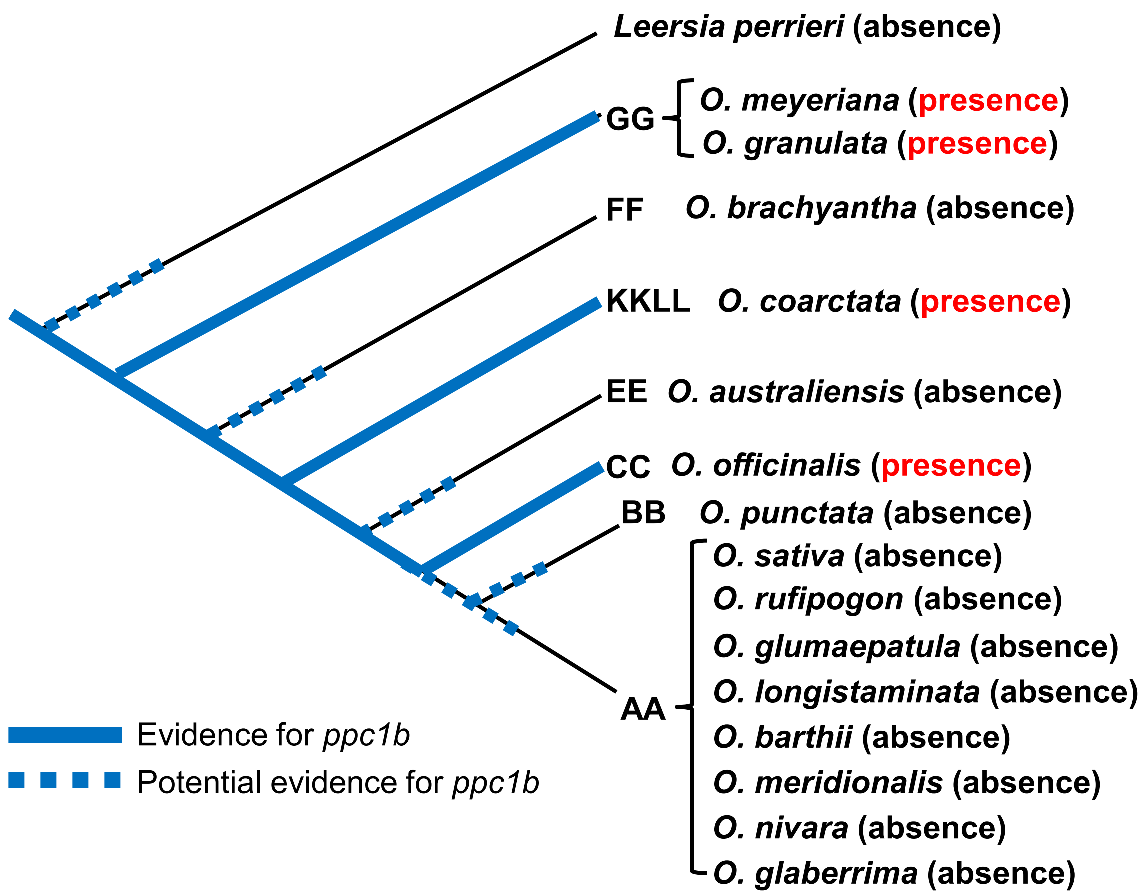

Supplementary Figure S9. An evolutionary chart of wild rice and cultivated rice represents the presence and absence of *ppc1b*.

posi	ppc-b	ppc4	ppc1a	ppc1b	ppcC_4	ppc3	ppc2a	ppc2b
61	A	V	V	V	V	V	V	V
62	Q	E	Q	Q	Q	Q	Q	Q
122	R	K	R	R	R	R	R	R
140	S	E	E	E	E	E	E	E
141	K	S	S	S	S	S	S	S
162	L	V	V	V	V	V	V	V
200	N	L	L	L	L	L	L	L
222	T	L	Q	Q	Q	Q	Q	Q
238	V	Q	Q	Q	Q	Q	Q	Q
245	L	M	M	M	M	M	M	M
247	I	Y	Y	Y	Y	Y	Y	Y
251	S	T	T	T	T	T	T	T
259	Y	F	F	F	F	F	F	F
264	S	D	D	D	D	D	D	D
278	L	Y	Y	Y	Y	Y	Y	Y
442	R	Q	Q	Q	Q	Q	Q	Q
486	K	R	R	R	R	R	R	R
525	G	P	P	P	P	P	P	P
529	L	F	F	F	F	F	F	F
531	A	A	A	A	P	A	A	A
551	K	R	R	R	R	R	R	R
553	A	C	C	C	C	C	C	C
582	L	F	F	F	F	F	F	F
627	A	V	V	V	V	V	V	V
628	C	A	A	A	A	A	A	A
657	Q	L	L	L	L	L	L	L
663	S	T	T	T	T	T	T	T
676	M	V	V	V	V	V	V	V
683	L	E	E	E	E	E	E	E
688	V	F	F	F	F	F	F	F
693	I	R	R	R	R	R	R	R
700	L	E	E	E	E	E	E	E
702	T	G	G	G	G	G	G	G
725	C	T	T	T	T	T	T	T
780	A	A	A	A	S	A	A	A
797	G	A	A	A	A	A	A	A
824	S	V	V	V	V	V	V	V
837	A	G	G	G	G	G	G	G
875	S	A	A	A	A	A	A	A
891	I	L	L	L	L	L	L	L
952	R	E	E	E	E	E	E	E
956	L	I	I	I	I	I	I	I
960	N	K	K	K	K	K	K	K
967	R	Q	Q	Q	Q	Q	Q	Q

Supplementary Figure S10. Amino acid sequence variations conserved in each PEPC group. The positions were referred to the maize C_4-photosynthetic PEPC.

Posi.	Substitution	Remarks
42	$V_{42}L$	Substitutions from ppc4/ppc1a/ppc1b to ppc3/ppc2a/ppc2b
100	$S_{100}K$	Substitutions from ppc4/ppc1a/ppc1b to ppc3/ppc2a/ppc2b
122	$R_{122}K$	Unique to ppc4
155	$G_{155}K$	Substitutions from ppc4/ppc1a/ppc1b to ppc3/ppc2a/ppc2b
185	$E_{185}Q$	Unique to ppc4
222	$Q_{222}L$	Unique to ppc4
364	$Q_{364}K$	Substitutions from ppc4/ppc1a/ppc1b to ppc3/ppc2a/ppc2b
495	$R_{495}N$	Substitutions from ppc4/ppc1a/ppc1b to ppc3/ppc2a/ppc2b
531	$A_{531}P$	Unique to $ppcC_4$
717	$M_{717}L$	Substitutions from ppc4/ppc1a/ppc1b to ppc3/ppc2a/ppc2b
753	$S_{753}L$	Substitutions from ppc4/ppc1a/ppc1b to ppc3/ppc2a/ppc2b
780	$A_{780}S$	Unique to $ppcC_4$

Supplementary Figure S11. List of amino acid substitution sites that are related to the molecular evolution of plant-type PEPC.

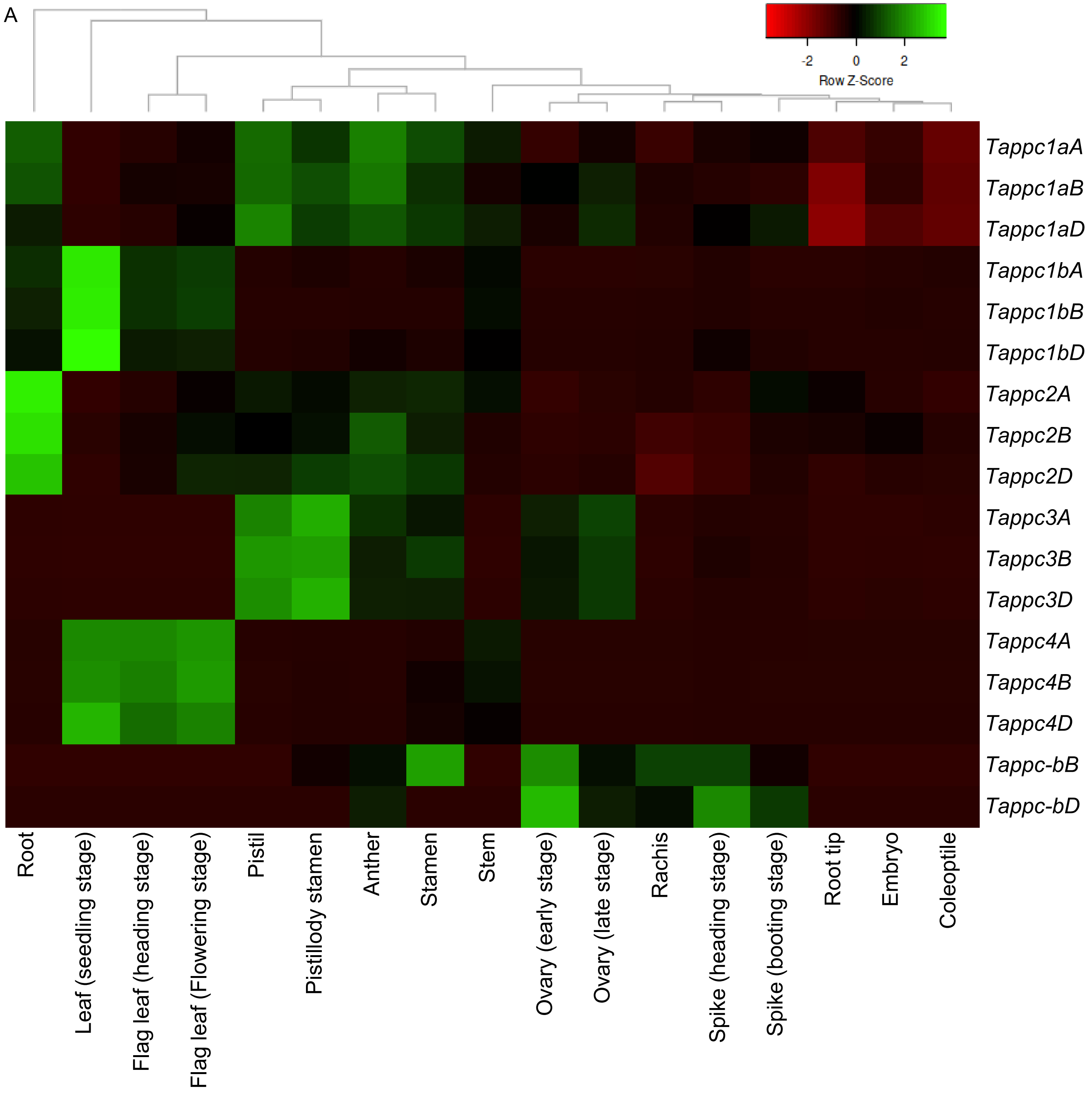

Supplementary Figure 12. Spatial gene expression patterns of wheat PEPC isogenes. (A) A heatmap represents gene expression patterns in various organs. (B) PCA biplot showing the interrelationship of *Tappc* isogenes for gene expression patterns. (C) PCA score plot represents the variations of RNA-Seq profiles analyzed.

B

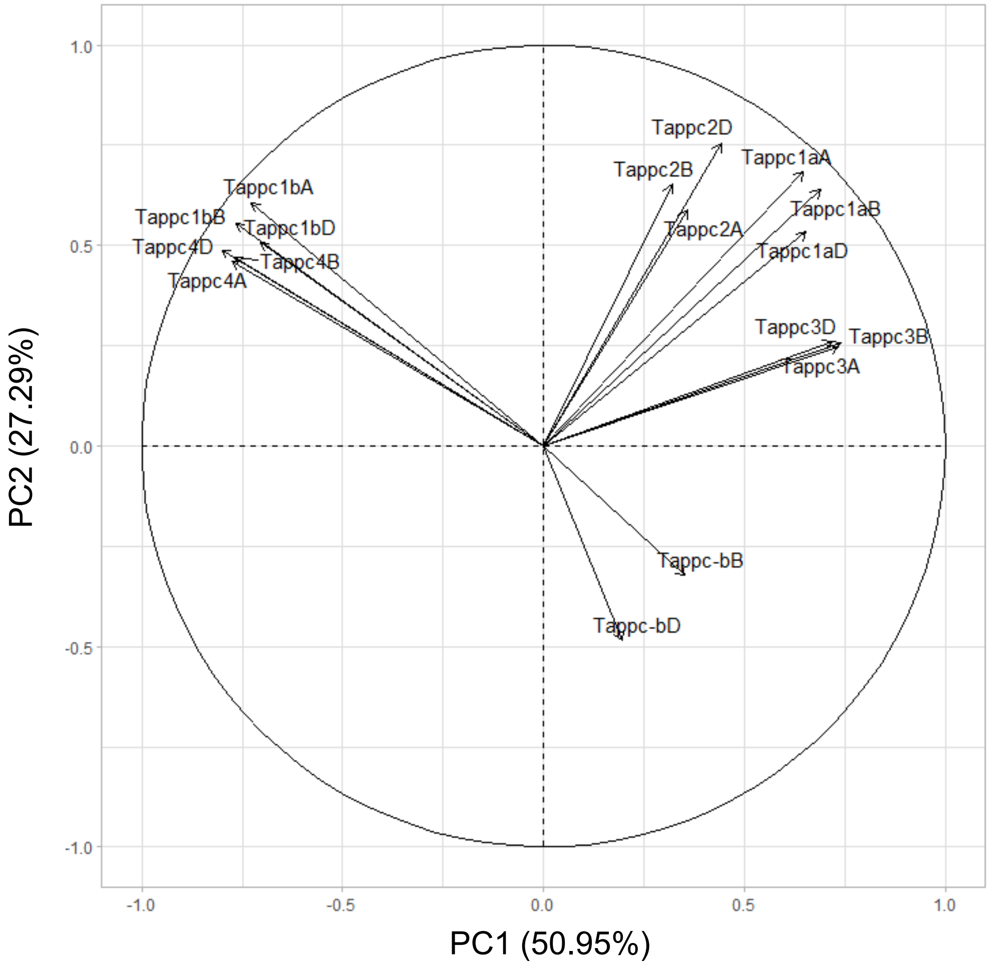

C

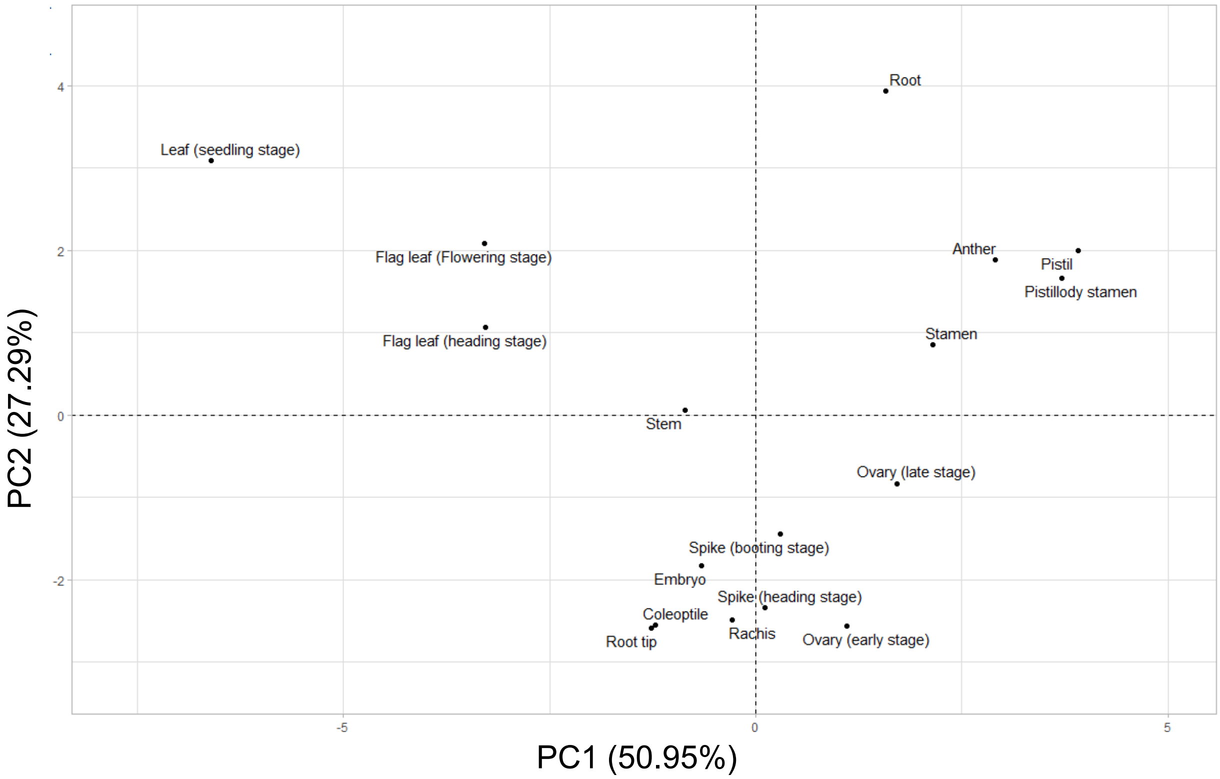

Supplementary Figure 12. Spatial gene expression patterns of wheat PEPC isogenes. (A) A heatmap represents gene expression patterns in various organs. (B) PCA biplot showing the interrelationship of *Tappc* isogenes for gene expression patterns. (C) PCA score plot represents the variations of RNA-Seq profiles analyzed.

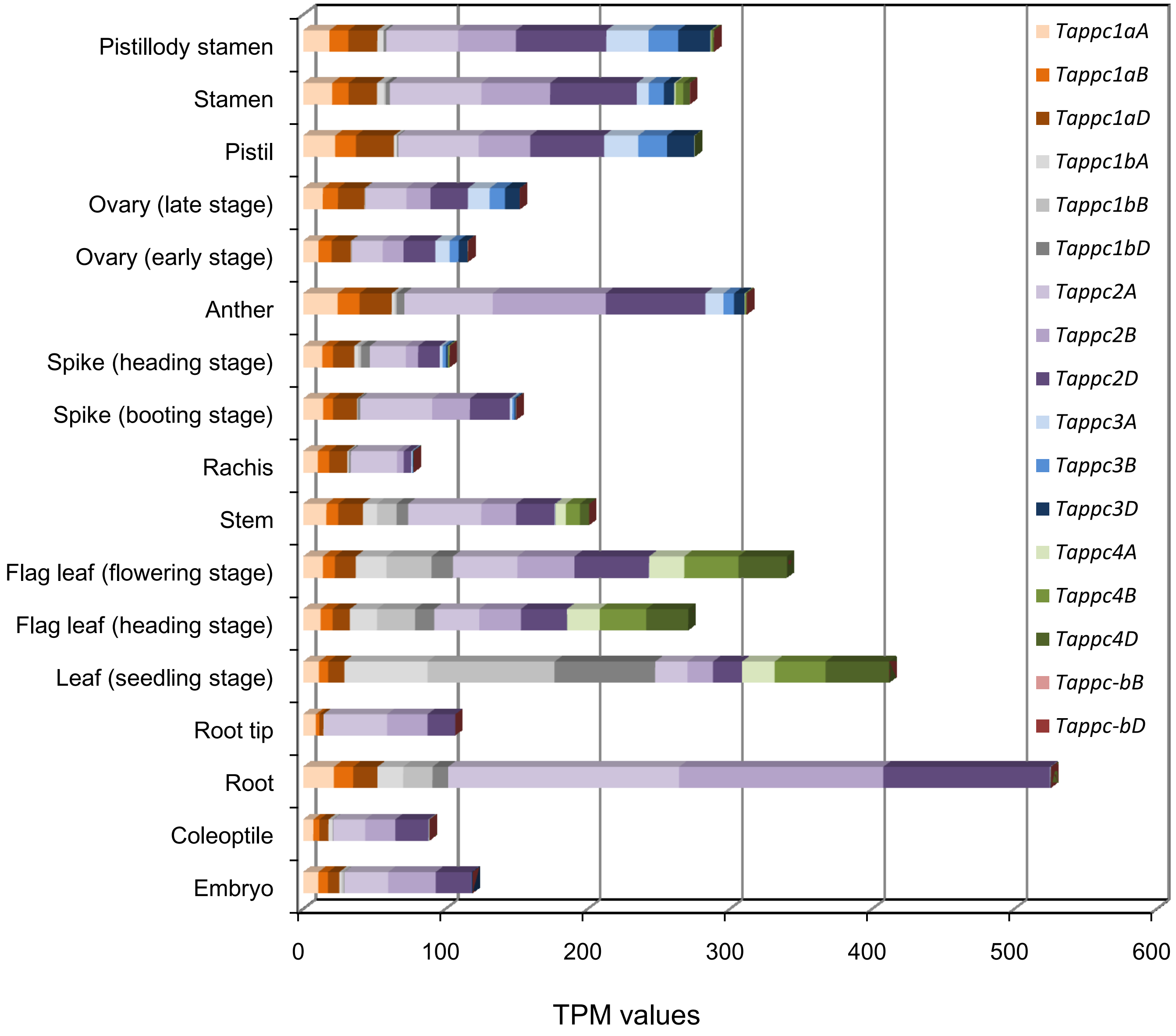

Supplementary Figure 13. A bar chart showing the compositions of quantitative gene expression levels of *PEPC* isogenes in wheat. The horizontal axis represents TPM values.

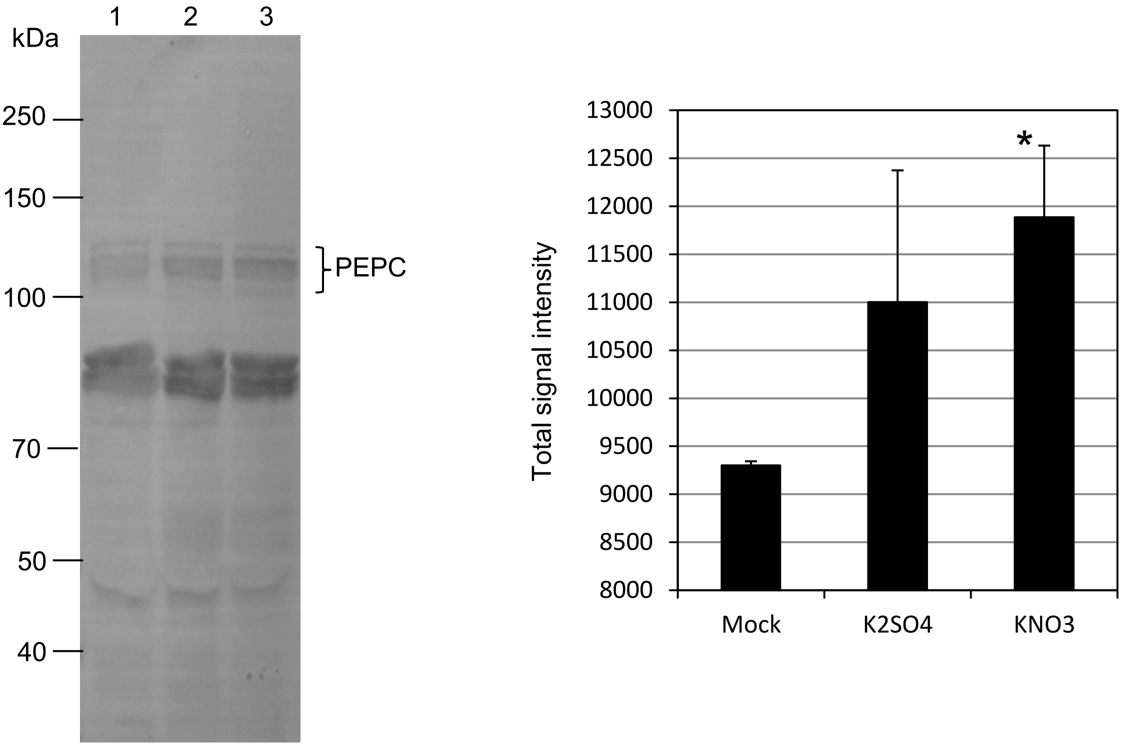

Supplementary Figure S14. Western blot analysis of PEPC in wheat detached leaves. The sample lane 1: Before detachment, lane 2: 24 hour with K_2SO_4, lane 3: 24 hours with KNO_3. Polypeptides representing nearby apparent size of PEPCs were designated as PEPC proteins. Signal intensities of PEPC proteins were calculated using ImageJ. The astarisk indicates statistical significance between “Mock” and “KNO_3“ samples at 10% level.

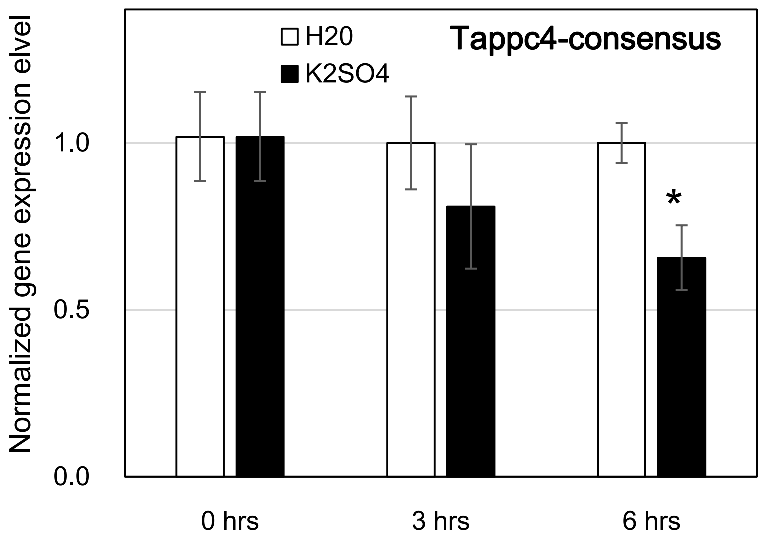

Figure S15. The effect of K_2SO_4 to gene expression of *Tappc4* in wheat detached leaves. Error bars indicate standard errors. The asterisk represents statistical significance at 5% level in Student's t-test.

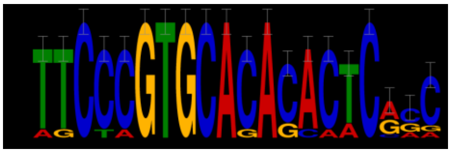

Motif TTCCCGTGCACACACTCRSC

E-value = 1.4e-023

Sequence name	Strand	Start	P-value		Site	
PAHAL_4G119200	+	2927	8.93e-13	AGGCCGCGCA	TTCCCGTGCACACACTCGCC	CGCTCCCCAT
GQ55_4G120100	+	2927	8.93e-13	AGGCCGCGCA	TTCCCGTGCACACACTCGCC	CGCTCCCCAT
Tappc1bD	+	2926	1.79e-12	GGCCGTACTT	TTCCCGTGCACACACTCAGC	AGTCACACAC
Tappc1bB	+	2803	1.79e-12	GGCCGTACTT	TTCCCGTGCACACACTCAGC	AGTCACATAC
BRADI_1g39167v3	+	2921	2.91e-12	GAGCCGTACT	TTCCCGTGCACACACTCAAC	CGTCACGTAC
Tappc1bA	+	2769	6.38e-12	GGCCGTACTT	TTCCCGTGCACACACTCCGC	AGTCACACAC
SETIT_005789mg	+	2952	1.14e-11	GAGGCGTGCA	ATCCCGTGCACACACTCGCC	GACTCCCCAT
OoffiChr06g01588	+	2888	3.15e-10	GCCGTACTTT	TTCTCGTGCACACACACGCG	CGCTATATCC
Zm00001d046170	+	2905	6.19e-10	GGGCCGCCCG	TTCCCGTGCACAGCCACACA	CACACCCGCC
Tappc4A	-	1370	5.35e-09	TGTGCTGTGG	TGCCAGTGCAGAGAATCAAC	AAATCTCAAA

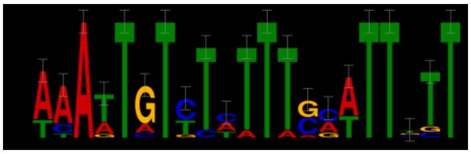

Motif AAATTGTYTHTTTSMWTTBTT

E-value = 1.9e-005

Sequence name	Strand	Start	P-value		Site	
Tappc4D	+	187	3.90e-12	ATTGCACTAA	AAATTGTCTCTTTGCATTGTT	AATAAGATGG
Tappc4B	+	1547	3.90e-12	CTTTGTACTA	AAATTGTCTCTTTGCATTGTT	AATAAGATGG
Tappc4A	+	1972	5.94e-11	ATTGTACTAA	AAAATGTCTCTTTGCATTGTT	AATAAGATTG
PAHAL_4G119200	+	149	2.14e-10	CTTTTTCTTA	AAAATGTCTATTTCAATTCTT	CTCATATTCT
GQ55_4G120100	+	145	2.14e-10	CTTTTTCTTA	AAAATGTCTATTTCAATTCTT	TTCATATTCC
Tappc1bB	-	362	1.78e-08	TTCTCTATTT	TTATTGTTTTTTTGATTTCTT	TCTTTTTCCT
Tappc1bD	-	506	1.91e-08	ATTTGGTTTT	TCATTATTTCTTTCATTTTTT	ATCATTTTTT
SORBI_3010G160700	-	2850	2.17e-08	AATAAGAAAG	AAATTGTTTATTTAGTTTATT	AAGTTAGACT
Zm00001d046170	+	1137	7.07e-08	TACAAATAAA	TCATTGTTTAATTCCATTTGT	AATAAGTTTT
Tappc1bA	-	2684	9.71e-08	AAAACTAATC	AAATTCTCCTTTACGATTTTT	TTTCAATCTA
SETIT_005789mg	-	235	1.20e-07	AAAAAGTATA	AAAGTATGTTTTTGCTTTTCT	GTGTTTTTGT

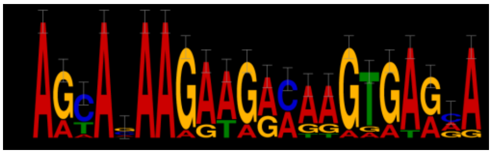

Motif AGCAVAAGAAGRMAAGTGARVA

E-value = 6.4e-010

Sequence name	Strand	Start	P-value		Site	
PAHAL_4G119200	+	1727	4.04e-13	ACACAACTGC	AGCAGAAGAAGACAAGTGAGGA	GTGACAGGTA
GQ55_4G120100	+	1727	4.04e-13	ACACAACTGC	AGCAGAAGAAGACAAGTGAGGA	GTGACAGGTA
SETIT_005789mg	+	1498	8.60e-12	GATTCGAGGA	AGCAGAAGGAGGCAAGTGAGCA	GTGACAAGTA
SORBI_3010G160700	+	2671	4.65e-11	CCAACCGAAT	AACACAAGAAGGCAGGTGAGCA	GTGACAAAGC
Zm00001d046170	+	2629	4.65e-11	CCAACGGAAT	AACACAAGAAGGCAGGTGAGCA	GTGACAAAGC
Tappc4B	+	1309	6.38e-10	ATATGCAACT	AGTACAAGATGAAGAGTGAAAA	TGTGAATAAC
Tappc4A	+	1736	6.38e-10	ATATGCAACT	AGTACAAGATGAAGAGTGAAAA	TGTGAATAAC
Tappc4D	-	521	1.89e-08	TAAATTAACC	AACATAAGAAGAATAGTGTAGA	TACTCCCTCC
Tappc1bB	+	59	3.02e-08	GAAGAATGAA	AGCAAAAGGAAACAAATAAAAA	AAGAAGAGAT
Tappc1bD	+	1640	4.63e-08	ATAGAAAACT	AGTAAAAAATGAAAAGAAAGAA	AAACCCAGGA
Tappc1bA	+	1539	6.29e-08	GATTGGGAGA	AGAAGAAGAAAGAATGGGAGCG	CACGGGG

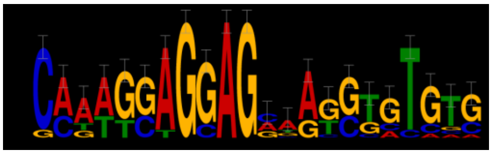

Motif CMAWGSAGGAGVAAKSTGTGTG

E-value = 3.0e-004

Sequence name	Strand	Start	P-value		Site	
Tappc1bB	-	2949	3.66e-12	CCGCGCCGAG	CAAAGGAGGAGGAAGCGGTGTG	TGCCTTCGCT
Tappc1bA	-	2937	3.66e-12	TCGCGCCGAG	CAAAGGAGGAGGAAGCGGTGTG	TGCCTCTCGC
Tappc4B	+	869	5.19e-10	GTTGCTTCAG	CAAATCAGGAGCAAGGTATGTC	AAATATATGA
Tappc4A	+	1293	5.19e-10	CTTGCTTCAG	CAAATCAGGAGCAAGGTATGTC	AAATATATGA
SETIT_005789mg	+	1852	1.73e-09	TTGCCAACGG	CAAAGGAGCAGCAGTGTGTGTA	GTTCGGCTAT
PAHAL_4G119200	+	2645	2.83e-09	AGTCCTGACT	CCTTGCAGGAGATGTGTGTGTG	AGCTAATAAG
GQ55_4G120100	+	2645	2.83e-09	AGTCCTGACT	CCTTGCAGGAGATGTGTGTGTG	AGCTAATAAG
Tappc4D_3kb	+	2492	3.47e-08	AAGTGGGCGT	CCAAGGAGGAGGAATGCCTCGC	CGAGGCATGG
Zm00001d046170	+	2081	5.31e-08	CGGTCGGCGC	CAATGGAGGAGACACCTGTACG	GTGCCCTTGG
SORBI_3010G160700	-	2549	8.68e-08	TTGCTTGCAC	GCGATGTGGAGAGAGGTGTCTG	TTAGCAACGG
Tappc1bD	+	1047	1.47e-07	TGTGCCTGTC	CAGTGGAGCAGCGAGCGCCGAG	CAAACTATAC

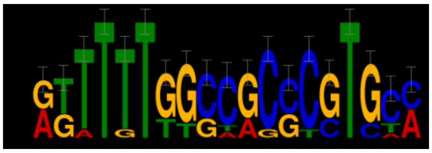

Motif RKTTTTGGCCGCSCGTGCM

E-value = 7.7e-007

Sequence name	Strand	Start	P-value		Site	
Tappc1bD	+	2866	3.91e-11	ATTTTTATTA	GTTTTTGGGCGCCCGTGCA	TCCGCTTTAG
Tappc1bB	+	2743	3.91e-11	ATTTTAATTA	GTTTTTGGGCGCCCGTGCA	TCCGCTTTAA
Tappc1bA	+	2709	8.78e-11	GAATTTGATT	AGTTTTGGGCGCCCGTGCA	CCCGCTTTAA
Tappc4D	-	1328	6.25e-10	GTGGCTAACG	ATTTTTTGCCACGCGTGCC	ATACGATTTT
Tappc4B	-	2962	6.25e-10	GTGGCTAACG	ATTTTTTGCCACGCGTGCC	ATCGATTTTT
Tappc4A	-	2960	6.25e-10	GTGGCTAACG	ATTTTTTGCCACGCGTGCC	ATACGATTTT
PAHAL_4G119200	+	2830	1.08e-08	CGACGGGTAT	GGTTTTGTCCGCCCCTCTC	CTCGCGGTGG
GQ55_4G120100	+	2830	1.08e-08	CGACGGGTAT	GGTTTTGTCCGCCCCTCTC	CTCGCGGTGG
SORBI_3010G160700	+	2213	7.70e-08	TGTGCGCGCT	AGATTTGGCAGCGCCTGAA	TGCTGTGCAG
SETIT_005789mg	+	1933	9.80e-08	AGGAGAGGGG	GTTTGTGGCTGGCTGTGCA	TCCATCCGTG

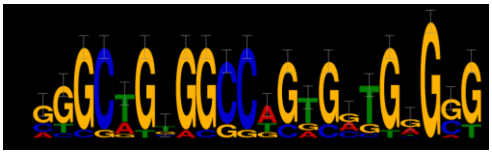

Motif SGGCTGKGGCCWGTGVTGRGSG

E-value = 6.1e-009

Sequence name	Strand	Start	P-value		Site	
Tappc4B	+	1217	3.96e-12	CCATGTTATC	AGGCTGTGGCCAGTGATGAGGG	AGGTGATGAT
Tappc1bB	-	2853	3.97e-11	TGGGATGATG	GGGCAGTGGCCTGTCGTGGGCG	TCTAATAGGC
Tappc1bA	-	2825	3.97e-11	TGGGATGATG	GGGCAGTGGCCTGTCGTGGGCG	TATAATAGGC
Tappc4A	+	1644	7.40e-11	CCATGTTATC	AGGCTGTGGCCAGTGACGAGGG	AGGTGATGAT
PAHAL_4G119200	-	1814	3.14e-10	GATATAACTC	CTGCTGCGGCCAGAGCTGAGGG	CTGATGTGCT
GQ55_4G120100	-	1814	3.14e-10	GATATAACTC	CTGCTGCGGCCAGAGCTGAGGG	CTGATGTGCT
Tappc1bD	-	2979	2.62e-09	.	GGGCAGTGGCCTGTGGGTGGGT	GTCTAAATAA
Tappc4D	+	2811	8.29e-09	GGGGGAGGGG	GGCCTGGGGCCGCGGCTGGGAG	CTAGGCCGTC
SETIT_005789mg	-	2190	5.51e-08	ACTACGATTG	GCGCTGAGGGCACTGATGCGCT	CGTTTTCGTA
SORBI_3010G160700	+	2187	5.83e-08	AAATCTGTGT	CGGCTTGACCCAGTGATGTGCG	CGCTAGATTT
Zm00001d046170	+	1479	6.50e-08	AGTGGATAGG	GGGGGGGGGGGGGGGGGGGGGG	GAGGGTCGTC

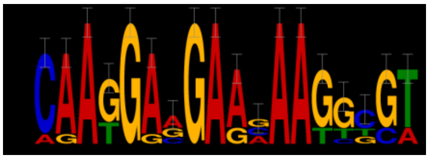

Motif: CAAKGAVGAAVAAGGCGT

E-value = 3.2e-003

Sequence name	Strand	Start	P-value		Site	
Tappc1bA	+	155	2.24e-09	ATGGATAGGC	AAAGGAGGAAGAAGGCGT	AGGTAAAAAG
Tappc1bB	+	2116	3.91e-09	TGTCCTTGTT	CAAGGACGAACAAGTGGT	CCACATCATC
Tappc1bD	+	2223	3.91e-09	TGTCCTTGTT	CAAGGACGAACAAGTGGT	CCACATCATC
Tappc4A	-	1766	2.24e-09	AATCTAATTA	CAATGAAGAAAAAGGTGT	TATTCACATT
Tappc4B	-	1339	2.24e-09	TATCTAATTA	CAATGAAGAAAAAGGTGT	TATTCACATT
Tappc4D	+	2493	1.23e-08	AGTGGGCGTC	CAAGGAGGAGGAATGCCT	CGCCGAGGCA
OoffiChr06g01588	-	324	2.24e-08	AGGAAGAAAG	CAATGGAGAAAAATGCGT	TGCCTTTCAA
SETIT_005789mg	+	2760	5.59e-09	GAGAAGGATT	CGAGGAAGAAGAAGGCGA	TTGAGCAGTG
Zm00001d046170	+	2694	4.21e-08	CAAAAAGGAG	CAAGGAGGAGCAAGCCCA	AGCCGCAGCC

Figure S16. Other potential cis-motif candidates that are associated with nitrate response.

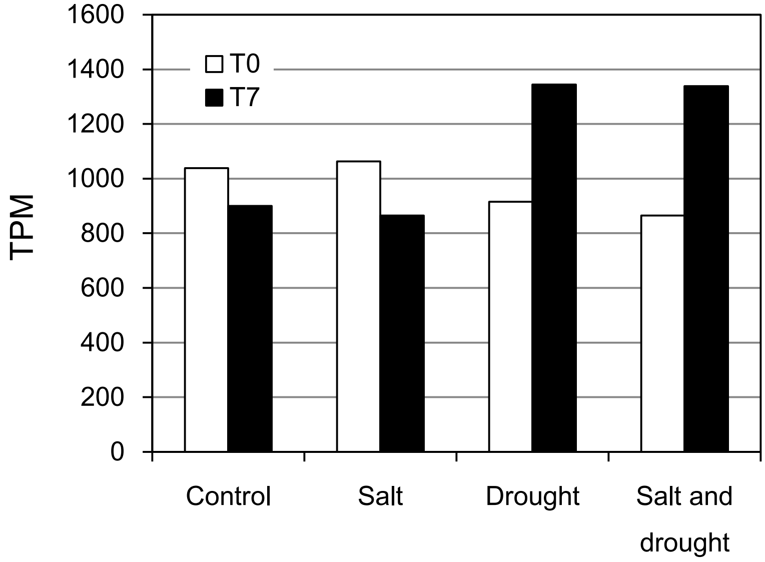

Figure S17. Gene expression patterns of maize C_4-photosynthetic *PEPC* under *a*biotic stress conditions. The data were retrieved from qTeller (Woodhouse et al. 2021).

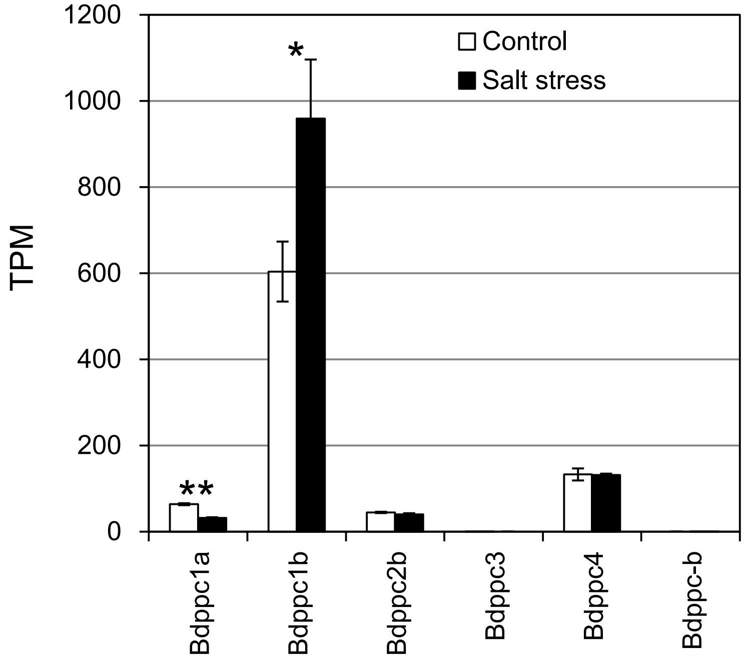

Figure S18. Selective up-regulation of *ppc1b* in leaves of three-week-old *B. distachyon* seedlings under a salt stress condition by 200 mM NaCl for 24 hours (Genbank SRA BioProject accession: PRJNA636626). Error bars indicate standard errors. The asterisk and double asterisk represent statistical significance at 10% and 5%, respectively.

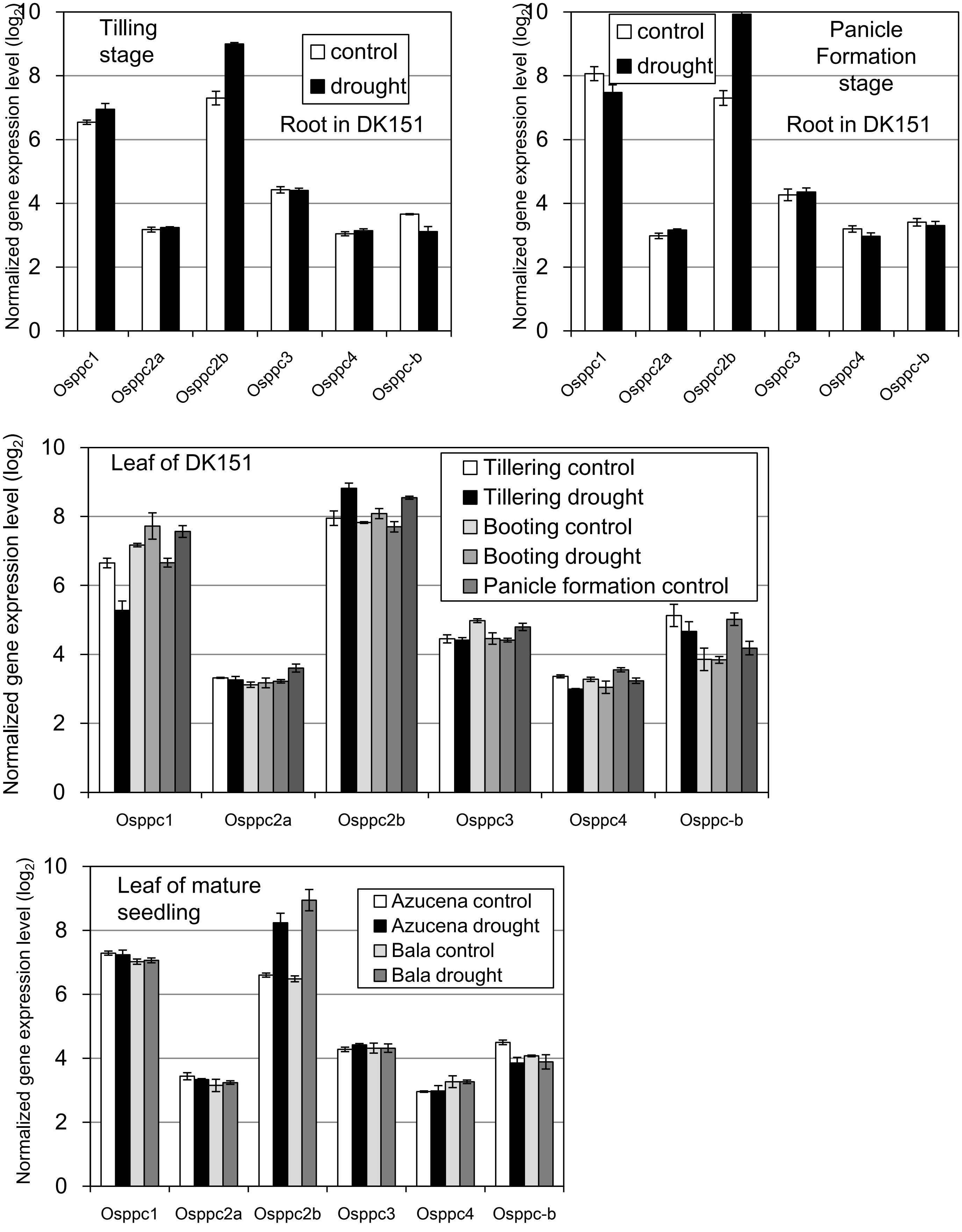

Figure S19. Rice microarray data represents selective transcriptional response of *Osppc2b* under drought stress conditions. The data were retrieved from OryzaExpress (Hamada et al. 2011). Error bars indicate standard errors.
